# Safety and efficacy analysis of in vivo lentiviral gene therapy in pre-clinical ARC syndrome models

**DOI:** 10.1038/s41467-026-73631-x

**Published:** 2026-06-19

**Authors:** Claudiu A. Cozmescu, Mina Nazari, Loukia Touramanidou, Sonam Gurung, Dany Perocheau, Neil Sebire, Yi-Ting Hu, Sian Goldsworthy, Jemima J. Burden, Youssef Khalil, Ivan Doykov, John R. Counsell, Rajvinder Karda, Sergi Castellano, Philippa Mills, Peter Clayton, Wendy Heywood, Dale Moulding, Simon N. Waddington, Julien Baruteau, Giandomenico Turchiano, Paul Gissen

**Affiliations:** 1 NIHR Great Ormond Street Hospital Biomedical Research Centre, London, UK; 2 Great Ormond Street Institute of Child Health, University College London, London, UK; 3 Laboratory for Molecular Cell Biology, University College London, London, UK; 4 Division of Surgery and Interventional Science, University College London; Royal Free Hospital, 9Th Floor (East), London, UK; 5 EGA Institute for Women’s Health, University College London, London, UK; 6 Metabolic Medicine, Great Ormond Street Hospital for Children NHS Foundation Trust; Great Ormond Street, London, UK

**Keywords:** Gene therapy, Genetic vectors, Liver diseases

## Abstract

Arthrogryposis, Renal dysfunction and Cholestasis (ARC) syndrome is a rare inherited disorder caused by defects in the VPS33B trafficking protein, leading to impaired bile flow, progressive liver disease and early death. No effective treatments are currently available. Gene therapy offers a potential approach by restoring the missing VPS33B protein in liver cells. Here, we show that liver-targeted lentiviral gene therapy safely and effectively rescues key features of ARC syndrome in a mouse model following in vivo administration by intravenous injection, combined with transient liver macrophage depletion. To assess the treatment efficacy, disease severity is exacerbated by 0.25% cholic acid diet feeds. A liver-specific vector shows a favourable safety profile over a ubiquitous vector. Mice receiving the safe liver-specific vector display improved survival, growth and liver function, reduced fibrosis and bile canaliculi restoration. These findings support targeted gene therapy as a promising treatment for ARC syndrome and related early-onset liver diseases.

## Abstract (web summary)

Cozmescu and colleagues show that systemic delivery of lentiviral gene therapy can improve disease features in a rare paediatric disorder (ARC syndrome). They show that liver-specific approaches prove safer than non-specific strategies, which may carry cancer risk.

## Introduction

Arthrogryposis, Renal dysfunction and Cholestasis (ARC) syndrome is a rare autosomal recessive disease characterised by congenital joint contractures, renal tubular acidosis, and neonatal cholestatic jaundice^1^. The disorder typically arises from deficiencies of one of the two proteins forming the C homologues in endosome–vesicle interaction (CHEVI) complex encoded by the vacuolar protein sorting 33 homologue B (*VPS33B*) and the VPS33B interacting protein, apical–basolateral polarity regulator (*VIPAS39*) genes^2^. In the liver, CHEVI defects impair the polarised distribution of bile canalicular transporters such as the bile salt export pump (BSEP) and multidrug resistance protein 3 (MDR3)^3^. This leads to hepatocellular damage, which eventually progresses to liver fibrosis, cirrhosis, and end-stage liver disease^1,3^. There is no curative treatment for this disorder, and most ARC patients die within the first year of life^1,4^.

Our group has shown liver phenotype rescue using adeno-associated vector (AAV) gene therapy after administration in *Vps33b* adult knock-out mice^3^. However, loss of transgene expression after neonatal administration, due to rapid liver growth and the inability to reinject the AAV vector due to robust immune response triggered by the first dose, limits the translational potential of AAV therapy in most infantile-onset liver disorders^5^. Recently, concerns regarding AAV genotoxicity have also intensified after the FDA placed a clinical hold on REGENXBIO’s AAV therapies RGX-111 and RGX-121 following a CNS tumour case, although causality remains unproven and no additional neoplasms have been reported^6^. Lentiviral vectors (LV) have received regulatory approvals in ex vivo gene therapies for monogenic disorders and have been successfully delivered in vivo through local administration in clinical trials for Parkinson’s disease^7–10^. More recently, they have also showed favourable safety profiles in early-phase in vivo clinical trials, including emerging systemic delivery for direct immune-cell engineering, such as in vivo CAR-T approaches^11^. Notably for monogenic liver disorders, Genespire’s GENE202 has recently been granted orphan drug designation by both the FDA and the European Commission as a systemic LV therapy for methylmalonic acidemia and is expected to progress to clinical trials in 2026^12^. LVs integrate their payload into the genome of hepatocytes, ensuring sustained transgene expression despite liver growth and therefore potentially overcoming the shortcomings of AAVs^13^.

Here, we show that lentiviral gene therapy can safely and effectively treat hepatic disease in ARC syndrome. We generate and evaluate a liver-specific (LP1-VPS) and a ubiquitous (EF1-VPS) vector in vitro and in vivo. Both vectors improve disease-relevant cellular phenotypes in vitro. However, in vivo safety assessment in heterozygous *Vps33b*^*Liver+/-*^ mice reveals liver tumour formation following EF1-VPS treatment, whereas no tumours are observed with the LP1-VPS vector. In homozygous *Vps33b*^Liver-/-^ mice, LP1-VPS administration normalises multiple disease biomarkers to wild-type levels and restores bile canaliculi structure.

## Results

### LP1*-*VPS and EF1*-*VPS lentiviral vectors rescue *VPS33B* knock-out phenotype in vitro

**Fig. 1:**
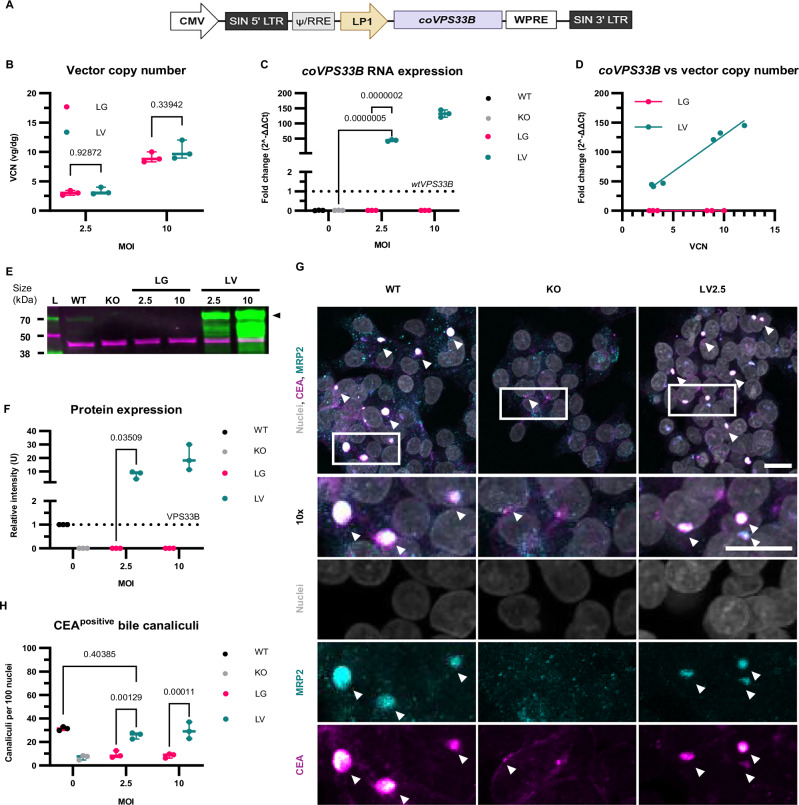
A liver-specific lentiviral vector (LP1*-*VPS) restores VPS33B expression and function in a *VPS33B*^−/−^ HepG2 cell model. **A** Schematic of the lentiviral vector backbone elements created with BioRender.com: LP1 – Liver promoter 1 comprising of the human apolipoprotein hepatic control region (HCR) and the human alpha-1-antitrypsin (hAAT) gene promoter, coVPS33B – codon optimised human *VPS33B* gene. In vitro testing in KO cells transduced with LP1*-*VPS (LV, *n *= 3) vs. control LP1*-*GFP (LG, *n* = 3) at multiplicity of infection (MOI) 2.5 and 10. **B** Post-treatment vector copy number (qPCR, *n* = 3 independent replicates). **C** Expression of *coVPS33B* mRNA compared to endogenous *MDH1* (qPCR), normalised to wild-type levels (*n = *3 independent replicates). **D** *coVPS33B* mRNA expression correlates with vector copy number (r = 0.9900; Pearson’s test). **E** Western blot analysis for VPS33B (green, black arrow) and β-actin (magenta). **F** Quantification of western blot VPS33B relative fluorescence intensity compared to β-actin and normalised to wild-type levels (*n *= 3 independent immunoblots). Functional rescue was assessed by localising carcinoembryonic antigen (CEA, magenta) and MRP2 (cyan) at bile canaliculi-like structures in HepG2 cells seeded in ibidi slides, with enhanced canaliculi formation via Oncostatin M. **G** Representative images of WT, KO, and LV2.5-treated cells; white arrows indicate bile canaliculi-like structures (scale bar: 100 μm; 5x electronic magnification insert, scale bar: 100 μm). **H** Quantification of CEA^+^ bile canaliculi per 100 nuclei (*n =* 3 independent replicates). Graphs (**B**-**C**, **F**–**H**) show individual values, medians, and IQRs. Statistical analysis used one-way ANOVA with Tukey’s test (*p* < 0.05 was considered significant). Source data are provided as a Source Data file.

A self-inactivating lentiviral vector (LP1*-*VPS) carrying the codon optimised *VPS33B* under the control of the liver-specific promoter 1 (LP1)^14,15^ (Fig. 1A) was produced and tested in a HepG2 ^VPS33B-/-^ cell model (HepG2 KO) generated using CRISPR-Cas9 (Supplementary Fig. 1). These cells display mislocalised carcinoembryonic antigen (CEA) (Supplementary Fig. 1), thigh junction proteins (Supplementary Fig. 2) and the bile salt export pump (BSEP, Supplementary Fig. 3), similar to the ARC patients’ and *Vps33b* KO mice (*Vps33b*^fl/fl^-Alfp-Cre referred to as *Vps33b*^Liver-/-^)^3^. HepG2 KO samples and cells treated with the LP1*-*VPS (LV) or the LP1*-*GFP (LG, control) at a multiplicity of infection (MOI, number of lentiviral vector particles per cell) 2.5 and 10. Treatment with LP1*-*VPS led to a significant increase in vector copy number (VCN, Fig. 1B), *coVPS33B* RNA expression (Fig. 1C, D), and resulted in high levels of VPS33B protein expression as measured by western blotting (Fig. 1E, F). Wild-type HepG2 cells form bile canaliculi-like structures that co-express CEA and multidrug resistance protein 2 (MRP2)^3^. HepG2 KO cells, which cannot form these structures, when treated with the LP1*-*VPS vector, regained this expression pattern (Fig. 1G, H).

**Fig. 2:**
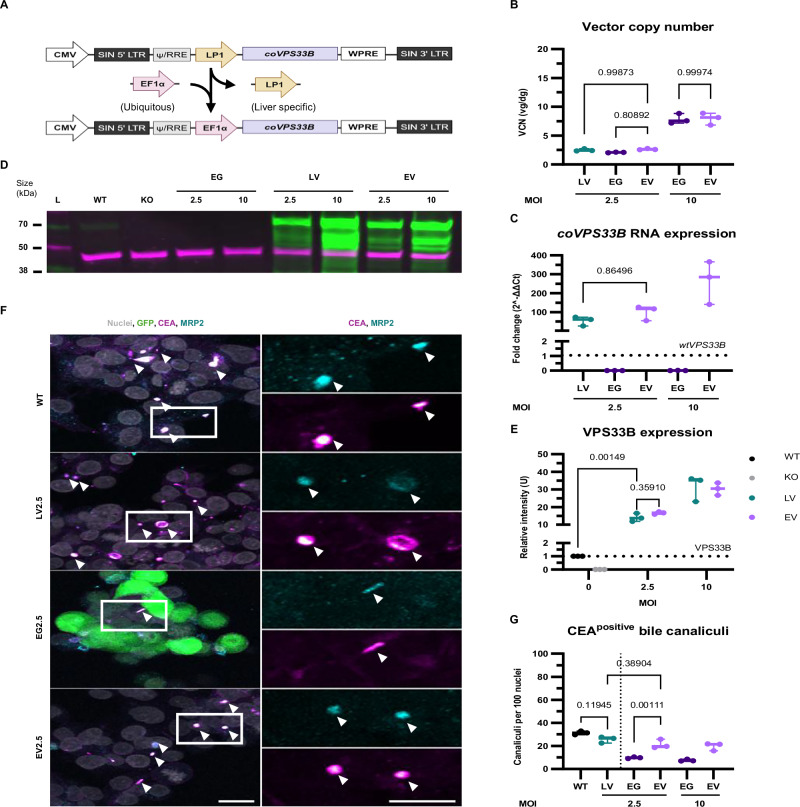
The EF1*-*VPS vector demonstrated stronger *VPS33B* expression and functional rescue similar to the LP1-VPS vector in HepG2 *VPS33B*^*-/-*^ cells. **A** Schematic of the lentiviral vector backbone elements created with BioRender.com: LP1 – Liver promoter 1 comprising of the human apolipoprotein hepatic control region (HCR) and the human alpha-1-antitrypsin (hAAT) gene promoter, coVPS33B – codon optimised human *VPS33B* gene. EF1α – eukaryotic translation elongation factor 1 alpha short promoter (EF1). The comparison was performed in vitro with *n = *3 samples of wild type (WT), HepG2 *VPS33B*^*-/-*^ (KO) cells and KO cells treated with either LP1*-*VPS (LV), EF1*-*GFP (EG) or EF1*-*VPS (EV) vectors. **B** Vector copy number (qPCR, *n* = 3 independent replicates). **C** Expression of *coVPS33B* mRNA compared to endogenous *MDH1* (qPCR), normalised to wild-type levels (*n* = 3 independent replicates). **D** Western blot of VPS33B (green, black arrow) and β-actin (magenta). **E** Quantification of western blot VPS33B relative fluorescence intensity compared to β-actin and normalised to wild-type levels (*n* = 3 independent immunoblots). Functional rescue was assessed by quantification of CEA^positive^ canaliculi (magenta) and presence of MRP2 (cyan) at bile canaliculi-like structures, enhanced by Oncostatin. **F** Representative confocal images with white arrows indicating bile canaliculi (Scale bars measure 100 μm, and 100 μm for the 5 x electronically magnified inserts). **G** Quantification of CEA^positive^ bile canaliculi per 100 nuclei (*n* = 3 independent replicates). The WT and LV values are the same as in Fig. 1, as the staining experiment has been performed simultaneously for all samples. Graphs show individual values, medians, and IQRs. Statistical analysis used one-way ANOVA with Tukey’s test (*p* < 0.05 was considered significant). Source data are provided as a Source Data file.

ARC syndrome is a multisystem disorder^1^ which will require restoration of VPS33B in several tissues. Hence, we compared the safety and efficacy of LP1*-*VPS with the EF1*-*VPS vector containing ubiquitous EF1α promoter^16^, previously used in clinical studies^7,17^ (Fig. 2A). We found no difference in cell viability (Supplementary Fig. 4), and no difference (*p* = 0.9987) in the VCN post-transduction with EF1*-*VPS vectors compared to LP1*-*VPS (Fig. 2B). Despite this, the increase in *coVPS33B* RNA expression was approximately 2-fold higher for the EF1*-*VPS compared to LP1*-*VPS treated cells (54.29, *p* = 0.8650, Fig. 2C), but the protein level difference was minimal (*p* = 0.3591, Fig. 2D–F). Both treatments restored CEA^positive^ bile canaliculi numbers close to wild-type level and their co-expression with MRP2 in HepG2 *VPS33B*^*−/−*^ cells (Fig. 2G, H).

### Safety studies in a *Vps33b*^Liver+/-^ mouse model

**Fig. 3:**
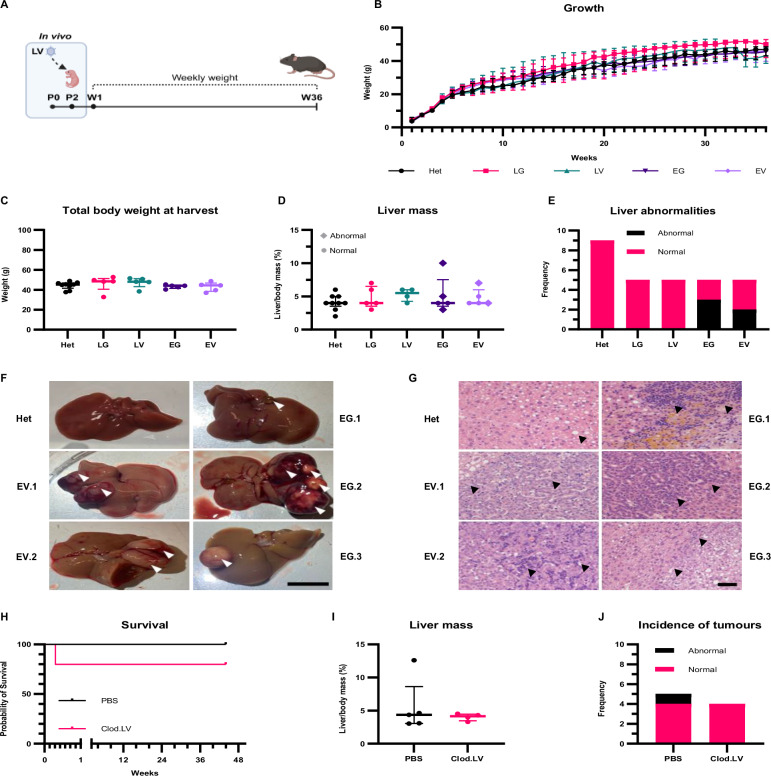
The liver-specific LP1*-*VPS vector showed no carcinogenic risk in *Vps33b*^*Liver+/-*^ heterozygous mice or *Vps33b*^*Liver-/-*^ mice pre-conditioned with clodronate. **A** In vivo safety study plan created with BioRender.com: *Vps33b*^Liver+/-^ heterozygous mice were injected at P2 with 5×10¹⁰ TU/kg of LP1*-*VPS (LV), LP1*-*GFP (LG), EF1*-*VPS (EV) or EF1*-*GFP (EG). Their weights were monitored for 36 weeks alongside wild-type littermates, followed by analysis at harvest. **B** Growth curves for WT (*n* = 9), LG (*n *= 5), LV (*n* = 5), EG (*n* = 5), and EV (*n *= 5) mice. **C** Total body weight for WT (*n* = 9), LG (*n* = 5), LV (*n *= 5), EG (*n *= 5), and EV (*n* = 5) mice. **D** liver-to-body mass percentage at harvest for WT (*n* = 9), LG (*n *= 5), LV (*n* = 4), EG (*n* = 5), and EV (*n* = 5) mice., with crossed diamonds marking liver abnormalities only found in EG and EV groups. **E** Frequency of liver abnormalities across groups. **F** Images of abnormal livers (white arrows; Scale bar measures 1 cm). **G** H&E staining shows liver samples from normal (WT) and abnormal (EV.1-2, EG.1-3) tissues, with black arrows marking atypical histology. The scale bar measures 100 μm. In vivo safety of the LP1*-*VPS (LV) vector was also validated in *Vps33b*^Liver-/-^ mice following the study plan (A) but pre-conditioned with clodronate (Clod) prior to the LV (Clod.LV, *n* = 5) or PBS (*n *= 5) administration. **H** Survival of Clod.LV treated animals compared with mock PBS- injected. The animal sacrificed in the first week post-treatment has been excluded from the long-term safety analysis. **I** Liver to body mass ratio of LV (Clod.LV, *n* = 4) or PBS (*n* = 5) treated experimental animals (**J**) Incidence of tumours in the experimental animals. Graphs C, D and I show individual values, medians, and IQRs, while B the medians and IQRs. Source data are provided as a Source Data file.

VPS33B has been reported to be a tumour suppressor protein, and its deficiency is associated with hepatocellular carcinoma (HCC)^18^. Hence, we selected heterozygous *Vps33b* knockouts (Het) for long-term safety testing. Neonatal *Vps33b*^Liver+/-^ mice were injected with LP1*-*VPS (*n* = 5), EF1*-*VPS (*n* = 5) or mock LP1*-*GFP (*n* = 5) and EF1*-*GFP (*n* = 5) vectors at 5 × 10^10^ TU/kg (Fig. 3A). The mice were monitored for 9 months before harvest and analysis. All the groups of treated mice showed similar growth (Fig. 3B, C) and liver mass (Fig. 3D). No liver abnormalities were observed in untreated *Vps33b*^Liver+/-^ mice or those treated with LP1*-*VPS or LP1*-*GFP vectors (Fig. 3E). In contrast, liver tumours were found in 3 of 5 mice injected with EF1*-*GFP and 2 of 5 with EF1*-*VPS vectors, (Fig. 3E–G) including 2 hepatic adenomas and 3 hepatocellular carcinomas (Fig. 3G). Only one mouse (EG.2) with a tumour had a substantial increase in liver-to-body weight ratio (Fig. 3D). The LP1*-*VPS safety was further evaluated in the *Vps33b*^Liver-/-^ disease model following the above protocol. To enhance hepatocyte transduction^19^, mice received intraperitoneal clodronate liposomes to transiently deplete liver and splenic macrophages^20,21^ prior to lentiviral vector (Clod.LV, *n* = 5) or PBS (*n *= 5) administration. Except for one mouse in the Clod.LV group, which was culled 2 days post-LV administration, all animals survived until the experimental endpoint (Fig. 3H) and displayed comparable median liver mass at harvest (Fig. 3I). No liver abnormalities were observed in Clod.LV treated animals, in contrast to a single PBS-treated *Vps33b*^Liver-/-^ mouse (Fig. 3J), which also had an increased liver mass (Fig. 3I). To further investigate the tumorigenic potential of the two EF1*-*GFP and EF1*-*VPS vectors, we have performed an identical experiment in wild-type animals, but no concerns were flagged.

**Fig. 4:**
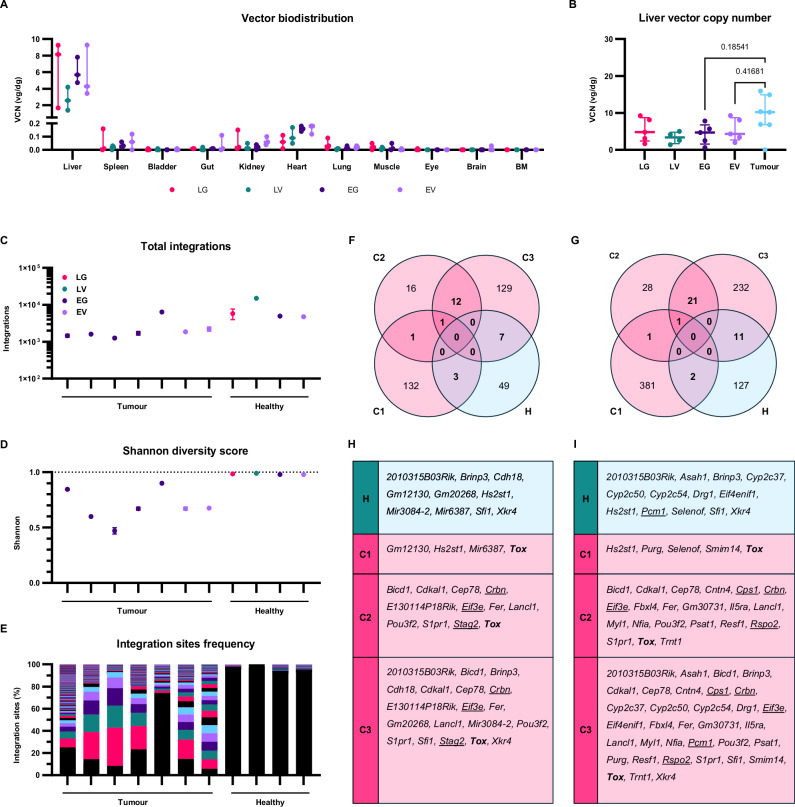
Vector integration analysis showed similar biodistribution across vectors but raised tumorigenic concerns with EF1*-*VPS and EF1*-*GFP vectors. *Vps33b*^Liver+/-^ heterozygous mice received 5×10^10^ TU/kg of LP1*-*VPS (LV), LP1*-*GFP (LG), EF1*-*VPS (EV) or EF1*-*GFP (EG) at P2. After 36 weeks, mice were sacrificed for organ collection and safety analysis. **A** Vector biodistribution assessed by qPCR in selected organs (*n* = 3 per group). **B** Vector copy number in healthy and tumour tissues from 36-week-old animals (LG, EG and EV: *n *= 5; LV: *n* = 4; Tumour: *n* = 7). Integration site analysis performed on DNA from tumour and control liver tissues. **C** Total integration sites measured in samples (*n =* 2, technical replicates per sample). **D** Shannon diversity score measuring integration site variety (*n* = 2, technical replicates per sample). **E** Integration site frequency with black areas representing integration events with a frequency below 0.1%. Integration events above 0.1% frequency were further examined. Sites from the same tumour were grouped as cancer clusters (C1, C2, C3), while healthy samples collected from LG, LV, EG and EV animals formed group H. Venn diagrams showing the number of genes with integration site frequencies > 0.1% (**F**) and genes within 100 kb of these sites (**G**), common across groups. **H**, **I** Lists of genes common between at least two groups in Venn diagrams (**F**) and (**G**), respectively, with the *Tox* gene in bold found across all cancer groups and known oncogenes underlined. Graphs A-B show individual values, medians, and IQRs, while graphs C and D display the median value and IQRs. Statistical analysis for B was performed by One-way ANOVA with Tukey’s multiple comparisons test (*p* < 0.05 considered significant). Source data are provided as a Source Data file.

Organ biodistribution of the integrated vectors was measured by qPCR in 3 randomly selected Het-mice per group (Fig. 4A), with the highest expression in the liver and a median VCN of 5.87 (IQR: 3.45-6.29). Liver VCN was further analysed across all groups for all experimental mice, showing similar values of 3.3 to 4.8 in healthy tissue (Fig. 4B).

To investigate the potential role of EF1-GFP and EF1-VPS vectors in carcinogenesis, genomic integration sites (IS) were sequenced from DNA extracted from tumours and healthy liver samples of treated animals using linear amplification–mediated PCR^22,23^. The aligned reads were deduplicated using sonic abundance to quantify each IS (Fig. 4C). The frequency of each IS was used to measure the Shannon Equitability Index, which ranges from 0 to 1, with 1 indicating no clonal proliferation. The healthy samples collected from LG, LV, EG and EV animals showed an index close to 1, while the tumour samples exhibited skewed ISs, with one event representing up to ~ 30% of all ISs and a relative Shannon Equitability Index of 0.46 (Fig. 4D, E). The integration sites with the top 5 IS frequencies for each cancer group are shown in Supplementary Table 1. Dominant integration sites and their frequencies varied across cancers: *Hs2st1* in C1 ( > 25% across all regions), *Cep78* in C2 (2.1%), *Arfgap2* in C3.1 (17.3%), and *Cdh18* in C3.2 (8.46%). IS with frequencies above 0.1% were further analysed to identify common genes either containing these sites or located within 100 kb of them in the three HCC samples and healthy tissues. For this, IS analysis results from different regions of each tumour and all healthy tissues were combined to create three cancer groups (C1, C2, C3) and one healthy group (H) (Fig. 4F–I). We identified 12 genes containing ISs (Fig. 4F, H) and 21 genes within 100 kb from IS (Fig.4G, I) that were common between at least 2 cancer groups and the *Tox* gene, which is common for all three cancer groups.

**Fig. 5:**
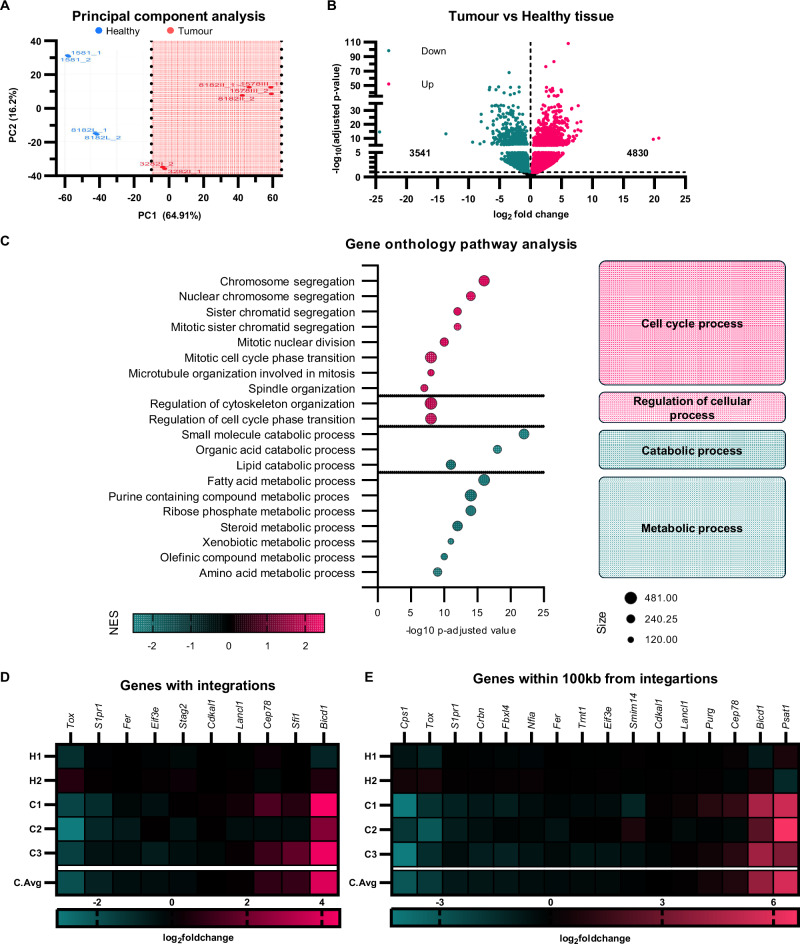
RNA-Seq analysis reveals gene expression dysregulation and pathway disruptions in HCC samples. **A** Principal component analysis (PCA) plots compare healthy (blue) and cancerous (red) liver samples, with variance percentages for each axis. **B** Volcano plots show differential gene expression (DEG) analysis between healthy and tumour samples, displaying significantly downregulated and upregulated genes (FDR-corrected p < 0.1) in cyan and pink respectivey, as well as non-significant genes in black. **C** Gene ontology analysis shows significantly dysregulated pathways (–log₁₀ p-adjusted), with positive normalised enrichment scores (NES) in pink and negative NES in dark cyan, clustered by common GO terms. The adaptive multi-level split Monte-Carlo scheme was used to estimate *p*-values. DEG analysis focuses on genes with > 0.1% integration site frequency **D** and those within 100 kb from such sites **E**, excluding genes common to both cancerous and healthy samples and those undetected in at least two RNA-Seq samples. Heat maps show log₂ fold changes, calculated as log₂(test/reference), where reference is the average normalised counts of healthy samples and test values are normalised counts of H1, H2, C1, C2, C3, and cancer average (C.Avg). Source data are provided as a Source Data file.

To explore potential mechanisms underlying vector-driven oncogenesis, we examined gene expression differences between HCCs in EF1*-*GFP and EF1*-*VPS treated *Vps33b*^Liver+/-^ mice and healthy liver tissues. To achieve this, we performed RNA-Seq analysis on RNA samples collected from 2 different regions of the analysed HCCs and healthy tissues. Principal component analysis showed two cancer samples clustering together and all cancer samples trending toward the right side of the PC1 axis. This indicated similar gene expression patterns in the cancer samples, in contrast to healthy liver samples, which clustered on the left (Fig. 5A). We then looked at the differential gene expression between tumour and healthy samples and detected 3,541 significantly downregulated and 4,830 significantly upregulated genes in tumours compared to healthy samples (Fig. 5B and Supplementary Table 2). Gene ontology pathway analysis revealed that the top 10 upregulated pathways are involved in cell cycle processes and regulation of cellular processes (Fig. 5C). In contrast, the top 10 downregulated pathways are involved in catabolic and metabolic processes (Fig. 5C). We also investigated if EF1*-*GFP and EF1*-*VPS integrations caused dysregulation of expression of genes either containing ISs (Fig. 4H) or within 100 kb of the ISs (Fig. 4I). Genes shared by both cancer and healthy tissues (Fig. 4H, I) or absent in at least two RNA-Seq samples were excluded from this analysis. We observed that *Bicd1*, *Cep78* and *Psat1* genes were upregulated while *Tox* and *Cps1* were downregulated when compared to the average normalised counts of those genes in healthy samples (Fig. 5D, E). Although the tumorigenic mechanism is not fully elucidated, EF1-VPS treatment showed signs of insertional oncogenesis. In contrast, the absence of tumours in animals injected with the LP1-VPS treatment, with or without clodronate, together with a Shannon equitability index close to 1, supports the safety of this vector for in vivo use.

### LP1-VPS rescues liver disease in *Vps33b*^*Liver-/-*^ mice

**Fig. 6:**
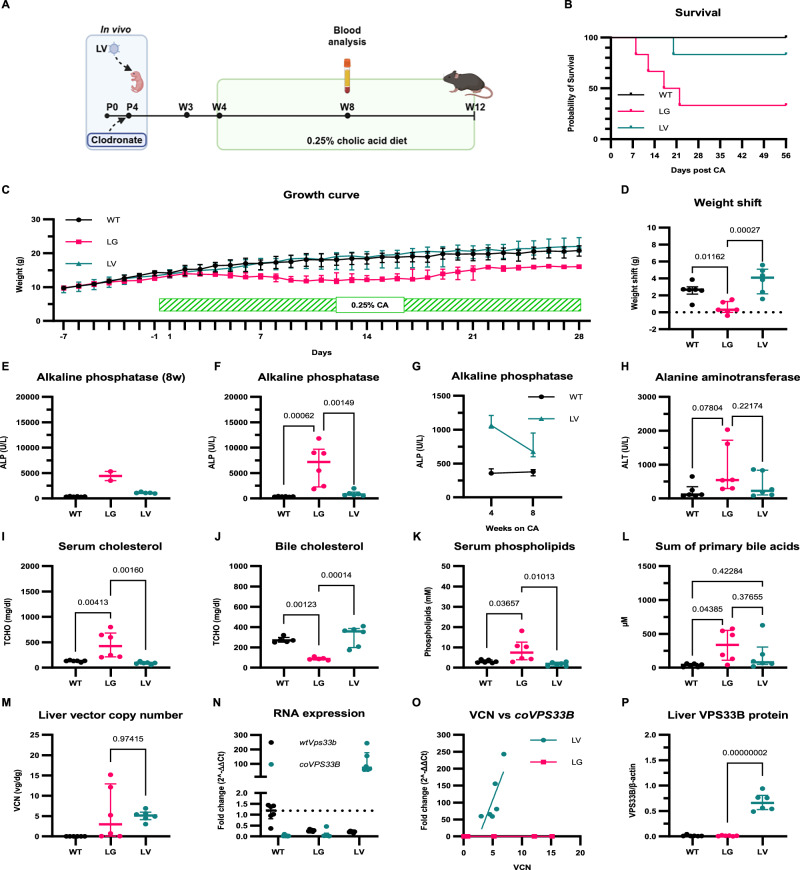
The LP1*-*VPS vector restores liver phenotype markers in the ARC syndrome *Vps33b*^*Liver-/-*^ mouse model upon restoration of gene expression. **A** Experimental plan created with BioRender.com: Neonate *Vps33b*^Liver-/-^ mice received 0.12 g/kg clodronate liposomes intraperitoneally 24 and 6 hours before 5 × 10¹⁰ TU/kg intravenous injection of LP1*-*GFP (LG,* n* = 6) or LP1*-*VPS (LV, *n* = 6) at P3-4. At 4 weeks of age, they were placed on a 0.25% cholic acid diet to induce cholestasis along with wild-type (WT, *n* = 6) mice. Efficacy was measured at 8 weeks and at harvest. **B** Survival plot of mice recorded up to 4 weeks post-diet administration. **C** Median weight over one month post-cholic acid diet administration (green dashed rectangle). **D** Weight change within 1 week post-cholic acid diet (*n* = 6 for each group). Serum alkaline phosphatase levels collected from live animals at 8 weeks in WT (*n* = 6), LG (*n* = 2) and LV (*n* = 5) animals **E**, as well as at harvest (*n* = 6, for all groups) **F**. **G** Alkaline phosphatase trend from 4–8 weeks on the cholic acid diet in WT (*n* = 6) and LV-treated mice (*n* = 5). Measurement of serum **H** alanine aminotransferase, **I** total cholesterol and **K** phospholipids collected from all experimental animals (*n* = 6 for each group), as well as **J** bile total cholesterol from WT (*n* = 5), LG (*n* = 5) and LV (*n* = 6) animals. **L** Sum of primary bile acids analysed by mass spectrometry from dried blood spots collected at harvest (n = 6, for all groups). **M** Vector copy number (qPCR, *n* = 6 for each group). **N** Expression of *wtVps33b* and *coVPS33B* compared to *Hprt* and normalised to average WT *wtVps33b* levels (qPCR, *n *= 6 for each group) **O** Correlation between *coVPS33B* RNA fold change and VCN in LG and LV samples (*r* = 0.7848, Pearson’s test). **P** VPS33B protein measured by mass spectrometry (*n* = 6 for each group). Graphs (**D**–**F**, **H–N** and **P**) show individual values, medians, and IQRs, while graph **C** and **G** shows the medians and IQRs. Statistical analysis by One-way ANOVA with Tukey’s multiple comparisons test (*p* < 0.05 considered significant). Source data are provided as a Source Data file.

After confirming the safety of the LP1*-*VPS vector in vivo, we tested its efficacy in *Vps33b*^Liver-/-^ mice^3^. Initial experiments have revealed a phenotypic drift when using the established protocol of liver phenotype induction by 0.5% cholic acid (CA) feeds^3^ (Supplementary Fig. 5), which lead to acute liver injury and death, so the following experiments were conducted using a 0.25% CA diet. In addition, we conducted preliminary optimisation experiments to assess the impact of clodronate liposome administration on vector uptake (Supplementary Fig. 6). Based on these findings, intraperitoneal clodronate treatment was incorporated into the final protocol to transiently deplete liver and splenic macrophages^20,21^, enhancing liver transduction^19^. Neonatal *Vps33b*^Liver-/-^ mice were pre-treated with 0.12 g/kg intraperitoneal injections of clodronate liposomes at 24 and 6 hours before lentiviral vector administration. At P3-4, mice were injected with 5 × 10¹⁰ TU/kg of either the mock LP1*-*GFP (LG, *n* = 6) or LP1*-*VPS (LV, *n* = 6) and monitored for 3 months alongside wild-type mice (WT, *n* = 6). At 4 weeks of age, all animals were placed on a 0.25% CA diet until the experiment’s end at 12 weeks (Fig. 6A).

To determine LP1*-*VPS treatment effects on survival and growth, animals were monitored daily for a month on the 0.25% CA diet. By the end of the month, LP1*-*VPS -treated animals had an 80% survival rate, compared to 33.3% for mock-treated mice (*p* = 0.0255, Fig. 6B). Animals that received the LP1*-*VPS vector displayed a growth curve similar to wild-type (Fig. 6C) and gained four times more weight than mock-treated animals (0.30 g, IQR: − 0.010-1.27 g, *p* = 0.0003) in the first week of CA supplementation (Fig. 6D). After a month on the CA diet, the live LV-treated animals showed an average 4-fold reduction in the serum ALP levels (a biomarker typically highly raised in ARC patients)^1^ compared to the live mock-treated animals (Fig. 6E). At sacrifice, ALP levels in the treatment group had a median of 778 U/L, nearly 10 times lower than the mock group (7,201 U/L, *p* = 0.0015, Fig. 6F). Notably, ALP levels in LP1*-*VPS treated mice continued to decrease from 4 to 8 weeks on the CA diet (Fig. 6G), possibly indicating a competitive advantage of treated cells in liver regeneration. In addition, in LP1-VPS-treated animals, median total serum cholesterol levels significantly decreased to wild-type levels (92.0 mg/dl, IQRs: 72.5-108.5 mg/dl, *p* = 0.0016, Fig. 6I). In addition, total bile cholesterol showed a significant increase from 85 mg/dl in the mock-treated group to 358 mg/dl in treated animals (*p* = 0.0001, Fig. 6J). Similarly, total serum phospholipids were significantly reduced to wild-type levels (1.88 mM, IQRs: 0.75-2.68 mM, *p* = 0.0101, Fig. 6K) in therapeutically treated animals. Lastly, primary bile acids displayed a slight improvement in treated animals, though elevated levels were observed in the early-harvested LP1-VPS-treated mouse (Fig. 6L and Supplementary Fig. 7).

The analysis of VCN in the livers of LP1*-*GFP and of the LP1*-*VPS injected animals showed similar results when measured by qPCR, though variability was noted in the mock-treated group (Fig. 6M). The average VCN of 5, was found in the livers of LP1*-*VPS treated animals, showing an average 110-fold increase in *coVPS33B* RNA expression compared to the level of *wtVps33b* found in wild-type animals, as determined by qPCR (Fig. 6N). The *coVPS33B* RNA expression positively correlated with the LP1*-*VPS VCN (r = 0.7848, Fig. 6O). Restoration of human VPS33B protein has also been observed post-treatment (Fig. 6P).

### The LP1*-*VPS vector restores hepatocyte architecture

**Fig. 7:**
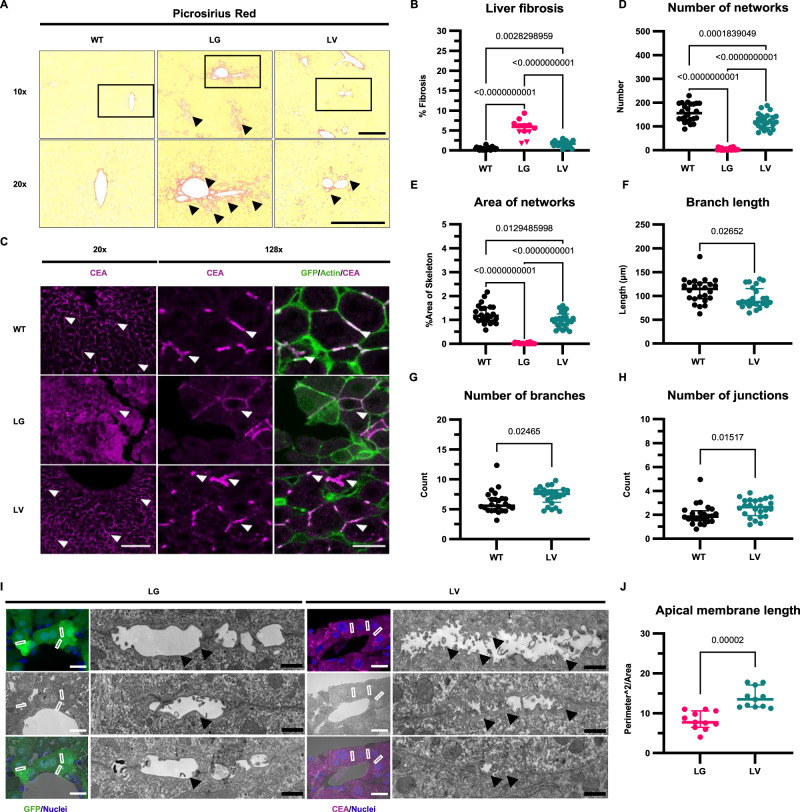
Correction of ARC syndrome liver phenotypes following LP1*-*VPS treatment in *Vps33b*^*Liver-/-*^ mice. Neonate *Vps33b*^Liver-/-^ mice received 0.12 g/kg clodronate liposomes intraperitoneally 24 and 6 hours before intravenous injections of 5 × 10^10^ TU/kg LP1-GFP (LG, *n* = 6) or LP1-VPS (LV, *n* = 6) at P3–4. At 4 weeks, treated and WT (*n* = 6) mice were placed on a 0.25% cholic acid diet to intensify cholestasis before liver collection at 12 weeks. **A** Picrosirius red staining showing fibrotic liver regions (black arrows) in images taken at 10 x magnification and inserts taken at 20 x magnification (scale bar measures 250 μm). **B** Fibrosis area was quantified from 5 × magnification images obtained from four different liver regions in WT (*n* = 6), LG (*n* = 3) and LV (*n* = 5) mice. Animals sacrificed under 3 weeks post-cholic acid were excluded, while those within 1 month are indicated by inverted triangles. Regions containing large blood vessels were omitted. **C** Immunofluorescence images of carcinoembryonic antigen (CEA, magenta), β-actin and GFP (green) staining taken at 20 x magnification (scale bar measures 100 μm) and 128 x electronic magnification (scale bar measures 15 μm). White arrows indicate bile canaliculi. Confocal images acquired at 20 × magnification from four liver regions per mouse were used to quantify the number of CEA^positive^ bile canaliculi networks **D** and the percentage of liver area covered by skeletonised CEA^positive^ canaliculi **E** (*n* = 6 animals per group - four regions analysed per liver).** F** Average bile canaliculi branch length in WT and LV-treated mice. Total numbers of canaliculi branches **G** and junctions **H** normalised to total network area (*n* = 6 animals per group - four regions analysed per liver). **I** Fluorescence-targeted TEM of bile canaliculi in LG and LV-treated livers. White rectangles mark regions analysed by EM, while black arrows indicate canalicular membranes. Scale bars measure 20 μm (low magnification) and 1 μm (high magnification). **J** Quantification of apical membrane length (Perimeter²/Area) (*n* = 11 bile canaliculi). Graphs (**C**–**H** and **J**) show individual values, medians, and IQRs. Graphs (**B**, **D** and **E**) were statistically analysed by one-way ANOVA with Tukey’s test (*p* < 0.05) and graphs (**F**–**H**) by two-tailed unpaired *t* test. Source data are provided as a Source Data file.

To gauge the impact of VPS33B restoration on liver architecture, we performed several histopathological, immunofluorescence and electron microscopy experiments. Haematoxylin & eosin (H&E) staining showed a moderate increase in immune cell infiltrates in surviving mock-treated 12-week-old mice versus LP1-VPS-treated animals (Supplementary Fig. 9). Picrosirius red staining revealed that control vector-injected mice had a higher degree of liver fibrosis (black arrows, Fig. 7A, B) than age-matched mice receiving the therapeutic vector. Wild-type livers showed intricate networks of immunofluorescently labelled CEA^positive^ bile canaliculi overlapping with dense regions of β-actin (bile canaliculi marker^24^ at higher magnification (white arrows, Fig. 7C–E). While in mock-treated animals, CEA canalicular localisation was lost, demonstrating diffuse hepatocyte membrane distribution, the LP1*-*VPS therapy restored CEA polarisation at bile canaliculi in large areas of the liver (white arrows, Fig. 7C–E). The average bile canaliculi branch length in LV-VPS-treated mice was approximately 15 μm shorter than in WT animals (111.2 μm, *p *= 0.0265, Fig. 7F). However, despite the reduced branch length, the bile canaliculi network appeared more complex following treatment, with a significant increase in branch number (*p* = 0.0264, Fig. 7G) and an average increase of 0.5 junctions (*p* = 0.0152, Fig. 7H). Fluorescence-targeted transmission electron microscopy (Fig. 7I, J) also revealed that the ultrastructure of bile canaliculi found in CEA^positive^ liver regions reverted to a morphology closer to the wild type^3^.

## Discussion

ARC syndrome is a fatal autosomal recessive multisystem disorder, resulting from mutations in either *VPS33B* (75%) or *VIPAS39*, genes encoding the two components of the CHEVI complex, which plays a crucial role in intracellular protein trafficking^1,2^. In hepatocytes, CHEVI appears to be the master regulator of apical membrane protein delivery. Its dysfunction results in the mislocalisation of numerous canalicular associated proteins, including BSEP, MDR3 and ABCB5, causing progressive liver damage and abnormal levels of various biomarkers in blood and bile^3^.

In this study, we evaluated the in vitro and in vivo safety and efficacy of two lentiviral vectors carrying a codon-optimised *VPS33B* gene in rescuing ARC syndrome-liver disease. We demonstrated that the liver-specific lentiviral vector (LP1*-*VPS) was safe for in vivo systemic delivery. The vector has shown high efficiency in improving several ARC-specific biomarkers such as ALP, total cholesterol, total phospholipids and bile acids, as well as liver fibrosis.

For the in vitro validation of our LVs, we have generated a HepG2 cell model of ARC that demonstrated VPS33B deficiency and abnormal localisation of canalicular membrane-associated protein CEA. This model could also be used for further assessing the trafficking at the bile canaliculi of other apical proteins, such as BSEP in health and disease. However, we have used CEA as a representative marker of ARC syndrome correction as it was shown to be mislocalised in previous publications^3^. Both vectors rescued the VPS33B expression, restoring the number of CEA^positive^ bile canaliculi and MRP2 localisation at the bile canaliculi.

Long-term clinical studies using ex vivo gene therapy with self-inactivating lentiviral vectors have suggested its safety compared to γ-retroviral vectors^25^. However, recent clinical research findings raised concerns about the possible oncogenic events, with a significant number of cases of myelodysplastic syndrome linked to the internal MNDU3 promoter used in the vector design^26^. This underscored the importance of evaluating the safety of the vectors developed for in vivoLV gene therapy for ARC syndrome. Our safety data for the liver-specific lentiviral vector aligns with previous studies that have used lentiviral vectors to target the liver, which demonstrated its safety and efficacy in canine models of haemophilia B^27^, as well as in murine and non-human primate models of haemophilia A^28^.

In contrast, 50% of the mice injected with the EF1*-*VPS or EF1*-*GFP containing EF1α promoter developed liver tumours, with 3 hepatocellular carcinomas confirmed by histopathological analysis. A slight increase in VCN in the tumour samples compared to the healthy tissue has prompted an investigation into the carcinogenic propensity of the two vectors. The IS analysis on samples collected from different regions of the three HCCs revealed clonal expansion of specific IS. Comparative analysis between the IS with a frequency above 0.1% in the three HCCs showed that *Tox* was a common integration locus for the vectors. In addition, 12 genes containing integrated vectors and 22 genes within 100 kb from IS were common between at least two of the HCCs analysed. Out of these, the expression of *Psat1*, *Bicd1* and *Cep78* were highly upregulated compared to their expression in healthy samples. Upregulation of *Psat1* and *Bicd1* genes has been previously associated with poor prognosis in hepatocellular carcinoma^29,30^. We also found *Tox* and *Cps1* expression to be downregulated in the tumour samples. While the loss of *Cps1* was previously observed to potentiate HCC metastasis^31^, downregulation of *Tox* has not been previously reported in HCC cells, and we do not consider it to be a likely driver mutation in this context. Nonetheless, not all HCC samples contained IS within 100 kb of these genes, suggesting their dysregulation is more likely a consequence of oncogenic cell transition rather than vector integration. Although self-inactivating lentiviral vectors are generally considered safe, vectors containing strong internal promoters (e.g., MNDU3^32^) can dysregulate gene expression and activate neighbouring promoters to at least 100 kb from the integration site, thereby enabling insertional oncogenesis independently of the targeted gene. With only three samples analysed, various vector integration-associated carcinoma-initiating mechanisms may not have been fully captured, and the oncogenic mechanism could have been different between the three tumours.

Among other oncogenic mechanisms involving the ubiquitous EF1α promoter, we considered the possible expression of VPS33B transgene in antigen-presenting cells, or insertion of the vector in other parenchymal cells such as hepatocyte progenitors, which have higher proliferative potential^33^. While we believe the first mentioned mechanism to be unlikely as this would lead to immune responses mounted against VPS33B positive hepatocytes and hence a lower VCN in tumour cells, the second option warrants further investigations. Although the precise mechanism underlying the genotoxicity observed with EF1α promoter containing vectors remains unclear, insertional oncogenesis, likely driven by promoter mediated dysregulation of nearby gene expression, remains the most plausible explanation. Whilst EF1α has proven safe in ex vivo gene therapy^7^, its oncogenic potential may be enhanced in a cancer-predisposed liver, such as in *Vps33b*^Liver+/-^ mice with reduced *Vps33b* expression^18^.

Strain-to-strain variation in the safety and efficacy of the lentiviral vectors targeting the liver has previously been noted^34,35^. Similarly, our safety findings in wild-type animals, where vectors containing the EF1α promoter did not lead to tumour development, contrasted with the data obtained from the *Vps33b*^Liver+/-^ mice. In our case, the genetic background of the mouse could have caused a different interaction of transcription factors with the LP1 and EF1α promoters, resulting in tumorigenesis. In addition, dysregulation of *VPS33B* expression has been previously seen in several cancers, including leukaemia^36^, colorectal carcinoma^37^, and hepatocellular carcinoma^18^. Specifically, downregulation of *VPS33B* has been shown to promote oncogenesis, suggesting that VPS33B may function as a tumour suppressor^18^. The *Vps33b*^Liver+/-^ mouse model, which has a single functional *Vps33b* allele in hepatocytes, may be prone to developing hepatocellular carcinoma. Together with our safety data, these findings support a two-hit model of cancer development^38^, where reduced VPS33B expression acts as a predisposing event requiring additional secondary alterations, possibly from vector integration combined with the stronger EF1α promoter-driven gene expression changes, to result in tumour formation. Thus, it might be speculated that this mouse model could be used in future safety studies for liver-directed gene therapy.

No carcinogenic events were observed in the livers of mice treated with the LP1*-*GFP and LP1*-*VPS vectors with or without clodronate administration, and their biodistribution was largely limited to the liver. Given this data supported the safety of the LP1*-*VPS vector for in vivo delivery, we have further investigated its therapeutic efficacy in a *Vps33b*^Liver-/-^ liver disease model^3^. We have demonstrated that the LP1*-*VPS treatment effectively reduced serum levels of ALP, total cholesterol, total phospholipids, and bile acids to levels at or near those found in wild-type controls. The treatment has also reduced the amount of fibrosis. Following treatment, large regions of the liver regained normal bile canalicular structure, including CEA localisation.

Our initial experiments have shown an average liver VCN from LP1*-*VPS-treated mice of approximately 0.2, resulting in liver *VPS33B* expression at about 63% of wild-type levels. This VCN was similar to other experiments where mice were treated with lentiviral vectors at a similar age^28,39^. This level of VPS33B expression was insufficient to ameliorate the ARC disease phenotype. Transiently depleting liver Kupffer cells with clodronate liposome treatment before LP1*-*VPS administration increased the VCN to 0.9 and the *VPS33B* gene expression to about 3 times the wild-type levels of expression. This has led to slightly improved markers of the ARC liver phenotype, but the real advancement in efficacy was achieved by a prolonged period of lower-dose CA administration. A 0.25% cholic acid dietary supplementation for 8 weeks resulted in a VCN increase to 5 and a 110-fold rise in *VPS33B* expression compared to wild-type levels, effectively correcting the liver phenotype over time, suggesting a possible effect of the competitive advantage of corrected cells repopulating the liver. These findings support the potential of lentiviral vectors as a therapeutic tool for a range of inherited liver disorders when combined with transient macrophage depletion and selective pressure.

Whilst the clodronate liposome approach used here to selectively deplete liver macrophages is not currently an established clinical intervention our findings indicate its utility to enhance lentiviral delivery to hepatocytes. Clinically relevant bisphosphonates, including clodronate, have been safely administered in patients through multiple routes^40,41^. Liposomal formulations of alendronate have demonstrated effective macrophage targeting and tolerability in non-human primate models^42^. Furthermore, in a double-blind clinical trial in diabetic patients undergoing percutaneous coronary intervention, liposomal alendronate was well tolerated without increased adverse events, supporting its clinical safety despite limited efficacy in this setting^43^. In addition to liposomes containing various types of bisphosphonates, other strategies are available, such as lentiviral vectors incorporating “don’t-eat-me” signals to reduce macrophage uptake^11,39^. These approaches suggest that overcoming the hepatic macrophage barrier may be clinically achievable. The requirement for a cholic acid diet reflects a limitation of the ARC syndrome murine disease model, as mice have a hydrophilic, muricholic acid–rich bile acid pool that attenuates cholestatic injury^44^. Such dietary intervention is not anticipated in patients, as human bile acids are naturally more hydrophobic and cytotoxic, providing natural selective pressure.

This study demonstrates that liver-directed gene therapy can be both safe and effective in treating cholestatic liver disorders such as ARC syndrome. The findings underscore the critical importance of achieving high transduction efficiency across target cells to ensure therapeutic impact. In addition, this work emphasises the need for rigorous, long-term safety evaluation of systemically delivered lentiviral vectors. Given the lentiviral vectors’ ability to integrate in actively transcribed genes^45^ it is crucial to consider potential variations in integration sites between murine and human hepatocytes, as these differences may influence the safety profile of the vectors. To ensure a complete evaluation of potential risks, further toxicological studies with larger sample sizes, along with a comparative integration site analysis between murine and human hepatocytes, are recommended before progressing the therapy to clinical application.

## Methods

### Study design

The objective of this study was to develop and assess the safety and efficacy of in vivo lentiviral gene therapy to treat the liver phenotype of ARC syndrome. For this, the human *VPS33B* gene was ordered from GeneArt with a 5’ Kozak sequence as well as 5’ AgeI and 3’ SalI cutting sites. Liver-specific pLP1-VPS and the ubiquitously expressed pEF1-VPS lentiviral vector backbones have been generated using enzymatic digestion and T4 ligation. The corresponding lentiviral viral vectors were then produced via transient transfection of Hek293T cells. In vitro studies were performed in HepG2 *VPS33B*^*-/-*^ cells (*n* = 3, for all experimental groups) for an initial validation of the vectors. In vivo mouse experiments were performed in compliance with the UK Home Office regulations under project licence number PP9223137 and personal license number I92863816. In all studies, animals were randomly distributed to experimental groups and no power calculations were used to predetermine the sample sizes for the study. Equal numbers of male and female animals were used for therapeutic efficacy experiments with no obvious difference between the sexes. Full details of materials, including name, catalogue number and manufacturer of the reagents, as well as the software used, can be found in the attached reporting summary and the supplementary file in Supplementary Tables 3, 4.

### Cell culture

Hek293T and HepG2 cells were cultured at 37 °C and 5% CO2 in Dulbecco’s Modified Eagle Medium (DMEM) supplemented with 10% (v/v) heat-inactivated foetal bovine serum (FBS) and 1% (v/v) penicillin/streptomycin (100 U/ml). For maintenance, at 70% confluence, Hek293T cells were dissociated using Trypsin EDTA (0.05%), while HepG2 cells were dissociated with Trypsin-EDTA (0.25%). Cells were subsequently centrifuged for 5 min at 500 x *g*, resuspended in fresh culture media and seeded as suitable for different culture dishes.

### Lentiviral vector production

To produce lentiviral vectors, Hek293T cells were seeded in T175 flasks. At 80–90% confluency, the cells were transiently transfected with the corresponding transgene plasmid along with Addgene pMDLg/pRRE (Cat. No. 12251), pMD2G (Cat. No. 12259) and pRSV-Rev (Cat. No. 12253) plasmids at a ratio of 4:2:1:1, respectively. The media collected at 48- and 72-hours post-transfection was centrifuged at 500 x *g*, filtered through a 0.22 μm and concentrated by ultracentrifugation for 120 min at 4 °C at 90400 x *g* in an Optima™ XPN-100 - IVD (Beckman Coulter). The viral titre was determined by culturing 5 × 10^4^ Hek293T cells with varying viral dilutions. DNA was extracted at 7 days, and 100 ng was analysed by qPCR using TaqMan Universal PCR Master Mix on a StepOne™ Real-Time PCR System. Lentiviral genomes were amplified using LV forward and reverse primers and an LV probe, while β-actin served as the endogenous control and was amplified with β-actin F and β-actin R primers alongside a β-actin probe. Standards ranging from 10^3^ to 10^7^ copies were included. Vector copy number (VCN) was calculated as: ${VCN}=\frac{{LV}.{Quantity}.{mean}}{{beta}-{actin}.{Quantity}.{mean}}*2$. Viral titre was determined as follows: ${Titre}=\frac{{VCN}*{{\mathrm{50,000}}}}{{viral\; volume}({ml})}$, then averaging across dilutions. For primers and probe details see Supplementary Table 3.

### Animal work

Animal work was conducted in compliance with the UK Home Office regulations under project licence number PP9223137 and personal license number I92863816. In accordance with the project licence, tumour-prone animals were regularly monitored and palpated during anaesthesia. Humane endpoints included palpable tumours or ascites causing impaired mobility or respiratory distress, at which point animals were euthanised by a Schedule 1 method. The maximal permitted tumour burden was not exceeded in any animal in this study. The C57BL/6 J *Vps33b*^*fl/fl*^-Alfp-Cre mice^3^ were rederived by the UCL Transgenic Services from frozen embryos, while wild-type C57BL/6 J (JAX Strain 000664) mice were purchased from Jackson Laboratory (Bar Harbour, ME). All the mice were maintained in a temperature-controlled environment with a 12 h/12 h light-dark cycle and ad libitum access to water and standard rodent chow (Cat. No. 2018C, Envigo). When phenotypic exacerbation was required, the standard chow was replaced by 0.5% or 0.25% cholic acid-supplemented chow (TestDiet® Europe, London, UK). For neonatal injections, the desired vector concentration, diluted in 40 µl PBS, was injected into the temporal vein using a tubed Hamilton syringe with a 33 G needle. Neonatal intraperitoneal injections with Clodronate Liposomes (Cat. No. F70101C-NH, TribioScience) were performed with a regular Hamilton syringe with a 33 G needle at 24 and 6 h before lentiviral vector administration.

### Immunofluorescence imaging prep of HepG2 cells

HepG2 cells seeded in 8-well µ-slides (Cat. No. 80826, Ibidi) at low density (5 × 104 cells/well) were cultured for 24 h before supplementing the media with 5 ng/ml Oncostatin to stimulate bile canaliculi formation^46^. After 48 h, the cells were fixed with 4% paraformaldehyde (PFA) for 12 min, followed by methanol (MeOH) fixation for 8 minutes. The cells were then washed three times with phosphate-buffered saline (PBS) and blocked for 60 min in 0.1% Tween PBS (PBS-T) containing 1% goat serum. Primary antibodies were then added, diluted 1:100, in blocking buffer, and the cells were incubated for 120 min at room temperature before two washes in PBS-T. Secondary antibodies, AlexaFluor 488 nm Goat anti-Mouse IgG (1:1000 dilution) and AlexaFluor 568 nm Goat anti-Rabbit IgG (1:1000 dilution) were then added for 60 min at room temperature and kept in a dark chamber. Cells were washed three times in PBS-T and once in PBS before being mounted in VECTASHIELD® Antifade Mounting Medium with DAPI.

### Western blotting

Protein immunoblotting was conducted using the NuPAGE® system (ThermoFisher) with 35–50 µg of total protein per sample. Samples were loaded onto a NuPAGE™ 4–12% Bis-Tris gel (Invitrogen) alongside a Chameleon Duo ladder (Li-COR) and run at 120 V for 50 min in NuPAGE MOPS SDS Running Buffer with antioxidant. Proteins were transferred to PVDF membranes (Bio-Rad) according to the manufacturer’s protocol. Membranes were blocked in 5% milk PBS-T for 1 h, followed by overnight incubation with primary antibodies diluted in blocking buffer rabbit anti-VPS33B (1:1000), mouse anti-Tubulin (1:5000), mouse anti-GAPDH (1:5000) or mouse anti-β-actin (1:5000). After washing, secondary antibodies were added and incubated for 2 h in the dark. Final washes were done in PBS-T and PBS, and membranes were imaged using an LI-COR Odyssey® CLx.

### Blood and bile analysis

Blood collection was performed under anaesthesia by superficial tail vein bleeding after 15–20 min of pre-heating at 37 °C in a heating chamber or by terminal bleeding through cardiac puncture. Serum was obtained from whole blood collected into micro sample tubes, CAT-Gel (Sarstedt) and centrifuged at 10000 xg for 5 minutes at room temperature. Bile samples were collected under terminal anaesthesia directly from the gallbladder using a 30 G needle. DRI-CHEM NX600 (Fujifilm, UK) system was used to measure the levels of alkaline phosphatase (ALP, Cat. No 16653964), alanine transaminase (ALT, Cat. No 16654035) and total cholesterol (TCHO, Cat. No 16654073) in 10 μl of sample per the manufacturer’s instructions. Total phospholipids were assessed using the phospholipid assay kit (Merck) per the manufacturer’s instructions.

### Liver processing

Perfusion was performed under terminal isoflurane anaesthesia to remove blood from the livers. For this, the mouse heart was exposed, the right atrium severed, and PBS injected into the left ventricle with a 20 ml syringe. Successful perfusion was confirmed by a light brown liver. Whole livers were collected, weighed on an Ohaus CL2000 scale, and dissected. Samples for DNA and RNA extraction were frozen on dry ice and stored at − 20 °C. Samples for H&E, Picrosirius Red, and immunofluorescence microscopy were fixed in 10% Neutral Buffered Formalin for 48 h at 4 °C temperature, then transferred to 70% ethanol and stored at 4 °C. Liver samples were processed and sectioned by UCL’s IQPath department using a Leica ASP300 automated tissue processor, embedded in paraffin (Leica EG1150H), and sectioned into 5 µm slices with a Leica RM2235 microtome. Sections were floated in a 42 °C water bath and mounted on Polysine adhesion slides (Thermo Scientific, 10219280), then stored at room temperature. Before use, slides were deparaffinized with two 5-minute washes in Histo-clear II (HS-202, National Diagnostics) and hydrated for analysis.

### VCN detection in mouse tissues

The vector copy number in the animal tissues was detected as described in the ‘Lentiviral vector production’. In contrast to the titering experiment, the reactions were started with variable amounts of DNA and the Titin gene was used as an endogenous control instead of β-actin. The sequences of the primers and probe targeting *Titin* can be found in Supplementary Table 3.

### Integration site analysis

VCN was remeasured for all samples using ddPCR (BioRad QX200). Lentiviral primers targeted the Psi vector region, while reference assays targeted the Titin gene in murine tissues. The Psi copy number was divided by the reference copy number and halved to determine the average VCN per diploid cell.

Integration site analysis (ISA) was conducted using linker-mediated PCR (LM-PCR) with modifications to established protocols^47^. Briefly, 500 ng of gDNA was fragmented and ligated to a double-stranded linker using NEB Next Ultra II FS DNA Library Prep reagents. Specific primers targeting the viral LTR included: LV_1PCR: 5’-CCCGTCTGTTGTGTGACT-s-C-3’, LV_Nested: 5’G-s-ACTGGAGTTCAGACGTGTGCTCTTCCG ATTCTGGTAACTAGAGATC CCTCAGA-s-C-3’, Linker_IPCR: 5’-GTAATACGACTCACTATAGGGC-3’; Linker_Nested: 5’-ACACTCTACACTCTTTCCCTACACGACGCTCTTCCGATCTAGGGCTCCGCTTAAGG GAC-3’, where the ‘s’ represents a phosphonothioate modification.

The PCR reactions were purified and barcoded with NEB Next Multiplex Oligos for Illumina (New England Biolabs). AMPpure XP beads purification (Beckman Coulter) was set at 0.7X volume to select fragments > 180 bp. The libraries were finally sequenced using the Illumina NovaSeq platform and analysed through the CAST-Seq Bioinformatic pipeline (https://github.com/AG-Boerries/CAST-Seq). True Integration sites (IS) were identified as events with more than 3 reads, which were then deduplicated and quantified using the unique molecular signature derived from the sonic abundance method. An in silico random IS library of 10,000 events was generated to perform a statistical comparison over the genetic features distribution and to control the clustering thresholds. The Shannon Diversity Index, utilised to measure the clonality of IS, was calculated as follows: $H=-\sum {pi}*{{\mathrm{ln}}}({pi})$; where H – Shannon Diversity index, pi – the proportion of the entire community made up of species ‘i’. The Shannon equitability index was calculated as: ${EH}=\frac{H}{{{\mathrm{ln}}}(S)}$; where EH – Shannon equitability index and S – the total number of unique species. This value ranges from 0 to 1, where 1 indicates complete evenness.

### Gene expression analysis by Quantitative Real-Time PCR

The high-capacity RNA-to-cDNA™ kit was used to convert 1 µg of RNA into cDNA following the manufacturer’s instructions. Gene expression analysis for the genes of interest (GOIs) was performed using quantitative real-time PCR with the Takyon ROX probe qPCR kit. The cDNA obtained as above was diluted 1:30 in RNase-free water before use. Primers for detecting wild-type human *VPS33B*, mouse wild-type *Vps33b*, codon optimised *VPS33B*, *GFP* and the housekeeping genes *MDH1* and *Hprt1* are listed in Supplementary Table 3. PCR reactions were conducted on a StepOne real-time PCR system (Applied Biosystems) using the default cycling conditions. All reactions were performed in triplicate, and gene expression levels were calculated using the ΔΔCt method^48^.

### RNA-Seq

The RNA-Seq was performed by the UCL Genomics facility to identify features that are differentially expressed between healthy and tumour liver samples collected from the lentiviral safety assessment cohort. Sequencing data was processed using nf-core/rnaseq (https://github.com/nf-core/rnaseq.git) of the nf-core collection of workflows^49,50^. Reads were pre-processed, including quality filtered and trimmed before being mapped to the GRCm38 genome, using software listed in the table set with default parameters. Mapped reads were de-duplicated and quantified to produce gene counts, which were used as input for differential gene expression. Differential gene expression was performed with the SARTools pipeline (V1.8.1)^51^ using DESeq2 (V1.40.2)^52^. All software used is available in the reporting summary file.

### Imaging of H&E and Picrosirius red stained samples

H&E and Picrosirius red-stained liver whole slide images were acquired using the Hamamatsu NanoZoomer S360 slide scanner. The images were then professionally analysed by a pathologist on the NZConnect 1.1.0 (IVD) platform, and crops at indicated magnifications were taken for publication and saved as ‘.tiff’ files.

### Immunofluorescence imaging prep of liver sections

For CEA-positive bile canaliculi detection, deparaffinized liver sections were rehydrated through 100% to 50% graded ethanol and rinsed with distilled water. Antigen retrieval was performed by boiling samples in citrate buffer for 20 min in a microwave at medium power, followed by cooling for 1 h at room temperature. Slides were rinsed in PBS-T and blocked for 1 h with a solution containing 10% foetal calf serum, 1% BSA, and 10% normal goat serum in PBS-T. Sections were incubated overnight with a rabbit anti-CEA primary antibody, diluted 1:100 in blocking solution, and washed 3 x 5 min with PBS-T. They were then incubated with fluorescent goat anti-rabbit secondary antibodies, diluted 1:300 in blocking solution, phalloidin–Alexa 488 nm (1:150) and DAPI (1 mg/ml; 1:1000) for 2 h in the dark. After three 5 min washes in PBS-T and one 5 min wash in PBS, slides were mounted with DPX resin.

### Confocal microscopy acquisition of immunofluorescently labelled samples

Images of immunofluorescently labelled cells and liver slices were acquired using the LSM710 Zeiss Confocal microscope and Zen black 2009 software. Cell images were captured at 25x NA0.8 with water immersion as 4-slice z-stacks. For liver sections, images were acquired at 20x NA0.8 and 63x NA1.4. At 10x NA0.45, z-stacks of five slices were captured at 0.96 µm spacing, while at 63x magnification z-stacks of twelve slices were captured at 0.32 µm spacing. All images were saved as ‘.tiff’ files for subsequent analysis using FiJi open-source software^53^. To improve clarity, confocal images displayed in this manuscript have been similarly enhanced. Quantification of CEA-positive bile canaliculi in HepG2 cells was performed on raw 20x images using a custom FiJi macro script developed in-house, available at (https://github.com/DaleMoulding/Fiji-Macros/blob/master/README.md#bile-canaliculi-assay).

### Fluorescence-targeted transmission electron microscopy (TEM) of liver sections

Liver samples were wedge injection fixed in 4% PFA for 5 min then stored overnight in 4% PFA at 4 °C prior to being sectioned with a vibrating microtome into 100 µm thick slices. The surface of the 100 µm slices was then immunolabelled with rabbit anti-CEA antibody, diluted 1:100 in PBS, for 24 h at room temperature. After three 15 min washes in PBS-T, samples were incubated for 2 h in PBS containing secondary antibodies diluted 1:1000 and DAPI (1 mg/ml; 1:1000). Following the incubation, the samples were washed three times for 15 min in PBS-T and once for 5 min in PBS before being transferred into a 2% formaldehyde, 1.5% glutaraldehyde in 0.1 M sodium cacodylate fixative. Slices were imaged using a 10x objective on a LSM710 Zeiss Confocal microscope, and regions displaying evidence of lentiviral transduction for example, GFP positive in the LP1-GFP treated mice, and Alexa-568 nm – CEA positive post LP1-VPS treatment, were trimmed down to small asymmetric regions of tissue and mapped at higher resolution using a 25x lens to capture the regions to be investigated by TEM. Once imaged by light microscopy, 100 µm liver slices were fixed overnight in 2% formaldehyde, 1.5% glutaraldehyde in 0.1 M sodium cacodylate. The following day, samples were treated with 1% tannic acid in 0.05 M sodium cacodylate, washed in water then dehydrated through as ethanol series (70, 90 and 100%) prior to infiltration series with Epon resin (50:50 Propylene oxide: Epon; 100% Epon and 100% Epon). After baking overnight at 60 °C, regions of interest, identified by the fluorescence imaging, were targeted for trimming and sectioning. Ultrathin sections were collected onto formvar coated slot grids (UC7 ultramicrotome, Leica; and Ultra45 diamond knife, Diatome), and bile canaliculi in regions where evidence of lentiviral transduction had been identified by light microscopy were imaged in the TEM (Tecnai T12 Biotwin, Thermofisher Scientific with Morada CCD using iTEM software, OSIS).

### Measurement of bile acids in dried blood spots by liquid chromatography-mass spectrometry

Dried blood spots were prepared for bile acid measurements by punching out a 6 mm spot and placing it in a 2 mL round-bottom tube. A 240 μl methanol solution was added, containing 25 nmol/L stable isotope internal standards of cholic and chenodeoxycholic acid and their taurine and glycine conjugates. Sixty microliters of deionised H2O were then added to the methanol solution. The bile acids were eluted into the methanol:H2O solution by sonicating the tube in a water bath for 15 min at room temperature. The solution was then transferred to a glass vial for LC-MS analysis. A calibration line was created for quantitation containing 20 nmol/L stable isotope internal standards, and the reference standards for cholic and chenodeoxycholic acid and their taurine and glycine conjugates with a concentration range of 0 – 250 nmol/L. Primary bile acids were calculated from their respective calibration line, assuming a 6 mm blood spot contains 10.3 μL blood. Secondary bile acids were semi-quantified using the primary bile acids calibration lines. The samples were analysed using a Waters Acquity UPLC coupled to an electrospray Xevo TQ-S triple quadrupole mass spectrometer. Five microliters of the methanol:H2O solution containing the extracted bile acids were analysed on an ACQUITY UPLC BEH C18 column (1.7 μm x 2.1 mm x 150 mm) using mobile phase A (0.01% formic acid) and B (Methanol), with a linear gradient at a flow rate of 0.25 mL/min as follows: Initial condition, 50% B gradually increasing to 99% B between 0.5 and 5 minutes and maintained for 2.5 min, after which the condition is returned to 50% B and maintained for 2.5 min with a total run time of 10 min]. The bile acids were analysed in negative ion mode by multiple reaction monitoring using the transitions listed in Supplementary Table 5. The mass spectrometer parameters were as follows: Capillary voltage = 2.7 kV; Source temperature = 150 °C; Desolvation temperature 600 °C; Cone gas flow = 150 L/Hr; Desolvation gas flow = 1200 L/Hr. Data was processed using TargetLynx software (Waters).

### Targeted proteomic quantification of VPS33B by LC–MS/MS

Liver tissue ( ~ 6 mg per replicate) was homogenised (Minilys bead homogeniser, Bertin Technologies) in 50 mM ammonium bicarbonate containing 0.2% (w/v) sodium deoxycholate using three 10 s high-speed pulses with cooling on ice between pulses. Protein concentration was determined by BCA assay (Thermo Fisher Scientific), and 100 µg total protein was adjusted to 100 µL in the same buffer. Samples were reduced with DTT (10 mM final)at 85 °C for 15 min, cooled, and alkylated with iodoacetamide (at room temperature in the dark for 45 min), followed by overnight tryptic digestion (Promega, 0.1 µg/µL) for ~ 10 h at 37 °C Digests were acidified with 5 µL 6% trifluoroacetic acid (TFA) for 10 min to precipitate deoxycholate, centrifuged at 21,000 × *g* for 10 min, and supernatants desalted using C18 solid phase extraction cartridges (Biotage). Peptides were vacuum-dried and reconstituted in 5% acetonitrile/0.1% TFA prior to analysis.

Targeted LC–MS/MS was performed on an ACQUITY I-Class Plus UPLC coupled to a Xevo TQ-XS triple quadrupole mass spectrometer (Waters). Peptides were separated on a Premier BEH C18 column (2.1 × 50 mm) at 50 °C and a flow rate of 200 µL/min. Mobile phase A was 0.1% (v/v) formic acid in water, and mobile phase B 0.1% (v/v) formic acid in acetonitrile. The gradient run time was 12 min, including equilibration (see Supplementary Table 6), and the autosampler was maintained at 10 °C. Needle washes consisted of ACN:H₂O:IPA:MeOH (1:1:1:1) for strong wash and 0.1% TFA in water for weak wash. Three VPS33B proteotypic peptides (IANVSILK, RPEIGHIFLLDR, SWQGLDEVVR) were quantified by MRM together with reference peptides from ACTB, GAPDH, PGK1 and PARK7. Transitions were optimised in Skyline (MacCoss Lab, University of Washington), and precursor/product ions, cone voltages, collision energies and quantifier/qualifier assignments are listed in Supplementary Table 7. Relative VPS33B abundance was calculated from peptide peak areas normalised to housekeeping peptide signals and expressed as target/reference ratios analogous to immunoblot loading controls.

### Statistical analysis

GraphPad Prism V10.0.2 software was used to generate graphs and perform statistical analysis when suitable. Unless otherwise mentioned, the graphs are individual dot plots displaying the median and interquartile ranges. A colour-blind safe colour scheme was applied to all the graphs. One-way ANOVA with Tukey’s correction was used for statistical analysis of data containing *n* > 3 samples per group. Values of *p* < 0.05 were considered significant. The graphs displayed *p*-values only for relevant comparisons, and non-significant values larger than 0.05 were displayed only at need. Correlation analysis was also performed using a Pearson correlation test.

### Reporting summary

Further information on research design is available in the Nature Portfolio Reporting Summary linked to this article.

## Supplementary information

Supplementary information

Reporting Summary

Transparent Peer Review file

## Source data

Source data

## Data Availability

All data associated with this study are included in the paper or the Supplementary Information. The integration site analysis data used in this study is available in the BioProject database under accession code PRJNA1458313. RNA-seq data discussed here has been deposited in NCBI’s Gene Expression Omnibus^54^ and is accessible through GEO Series accession number GSE329589. Protein mass spectrometry data presented in this study was deposited in the ProteomeXchange repository under the accession code PXD077802 https://panoramaweb.org/ZNP2AN.url. The mass spectrometry bile acid analysis raw data has been uploaded in the MetaboLights under the accession code MTBLS14408 https://www.ebi.ac.uk/metabolights/MTBLS14408. Source data are provided in this paper.

## Code availability

The Fiji macro script developed for bile canaliculi quantification is available at https://github.com/DaleMoulding/Fiji-Macros/blob/master/README.md#bile-canaliculi-assay.
