# Supplementary material for: Safety and efficacy analysis of in vivo lentiviral gene therapy in pre-clinical ARC syndrome models
Source: Nat Commun. 2026 Jun 19;17:5074. doi: 10.1038/s41467-026-73631-x (PMC13282399; doi:10.1038/s41467-026-73631-x)
Supplement: Supplementary file 1 — Supplementary information [file 41467_2026_73631_MOESM1_ESM.pdf]

Safety and efficacy analysis of *in vivo* lentiviral gene therapy in pre-clinical ARC syndrome models

Authors: Claudiu A. Cozmescu[1,2*], Mina Nazari[1,2], Loukia Touramanidou[2], Sonam Gurung[2], Dany Perocheau[2], Neil Sebire[1], Yi-Ting Hu[2], Sian Goldsworthy[2], Jemima J. Burden[3], Youssef Khalil[2], Ivan Doykov[1,2], John R. Counsell[4], Rajvinder Karda[5], Sergi Castellano[2], Philippa Mills[1,2], Peter Clayton[1,2], Wendy Heywood[1,2], Dale Moulding[1,2], Simon N. Waddington[5], Julien Baruteau[1,2,6], Giandomenico Turchiano[2], Paul Gissen[1,2,6*]

Supplementary Methods

gRNA design for HepG2 *VPS33B* knock-out

The CRISPOR online tool[1] was used to design and evaluate candidate gRNAs targeting the vacuolar protein sorting 33 homolog B (*VPS33B*) gene. A single gRNA was selected based on a high Doench '16 predicted efficiency score, a high MIT specificity score, and a strong out-of-frame score. To maximise the likelihood of functional gene disruption, the gRNA (5'-TATCGCATATTCTTGATGCG-3') was designed to introduce an early stop codon within the coding sequence (Supplementary Figure 1). As exons 2 and 3 are not present in all *VPS33B* isoforms, the selected guide targets exon 4, ensuring disruption across the majority of *VPS33B* isoforms.

Genomic DNA and RNA extraction from cells and tissues

Genomic DNA and RNA were extracted from cells and animal tissues using the DNeasy Blood & Tissue kit and the RNeasy kit, respectively, according to the manufacturer's instructions. On average $3x10^6$ cultured cells or 30 mg of tissue were used for each extraction. The DNA and RNA samples were eluted in 30 µl of nuclease-free water for cells and 100 µl nuclease-free water for tissues.

PrestoBlue cell viability assay

The viability of the cells was measured by PrestoBlue assay following the manufacturer's guidelines. In brief, 5x104 cells plated in a 24-well plate were cultured for 24 hours in complete DMEM before media was replaced with 200 µl of 1x PrestoBlue reagent diluted in DMEM. After a 10-minute incubation at 37°C, 100 µl of the substance was transferred to a black 96-well plate and its fluorescence was measured using a plate reader (Fluostar) at Ex/Em=560/590 nm.

Picrosirius Red Staining

The picrosirius red stain kit (Abcam) was used by the IQPath department at UCL's Institute of Neurology to stain fibrotic regions in liver sections. Sections were baked at 60°C for 1 hour, dewaxed using a Leica HistoCore Spectra automatic stainer, and hydrated in distilled water. They were then incubated in Picrosirius Red solution for 60 minutes, rinsed twice with acetic acid solution, and briefly rinsed in absolute ethanol. The sections were dehydrated with two changes of absolute alcohol, cleared with three changes of xylene, and mounted with DPX resin.

Haematoxylin and Eosin (H&E) Staining

Liver morphology was assessed using standard H&E staining, performed by the IQPath department at UCL's Institute of Neurology with the XL WORKSTATION (Cat. No. ST5010, Leica). Samples were dewaxed in xylene and hydrated through IMS, 70% ethanol and water. Sections were incubated in Harris Haematoxylin for 5 minutes, rinsed in water, and differentiated for 14 seconds in acid alcohol (1% HCL in 70% ethanol). After a 10-minute wash in water, samples were stained with 1% Eosin for 3 minutes, washed in water for 15 seconds, and dehydrated through 70% to 100% IMS. Finally, sections were cleared with xylene and mounted using DPX resin.

Supplementary Figures

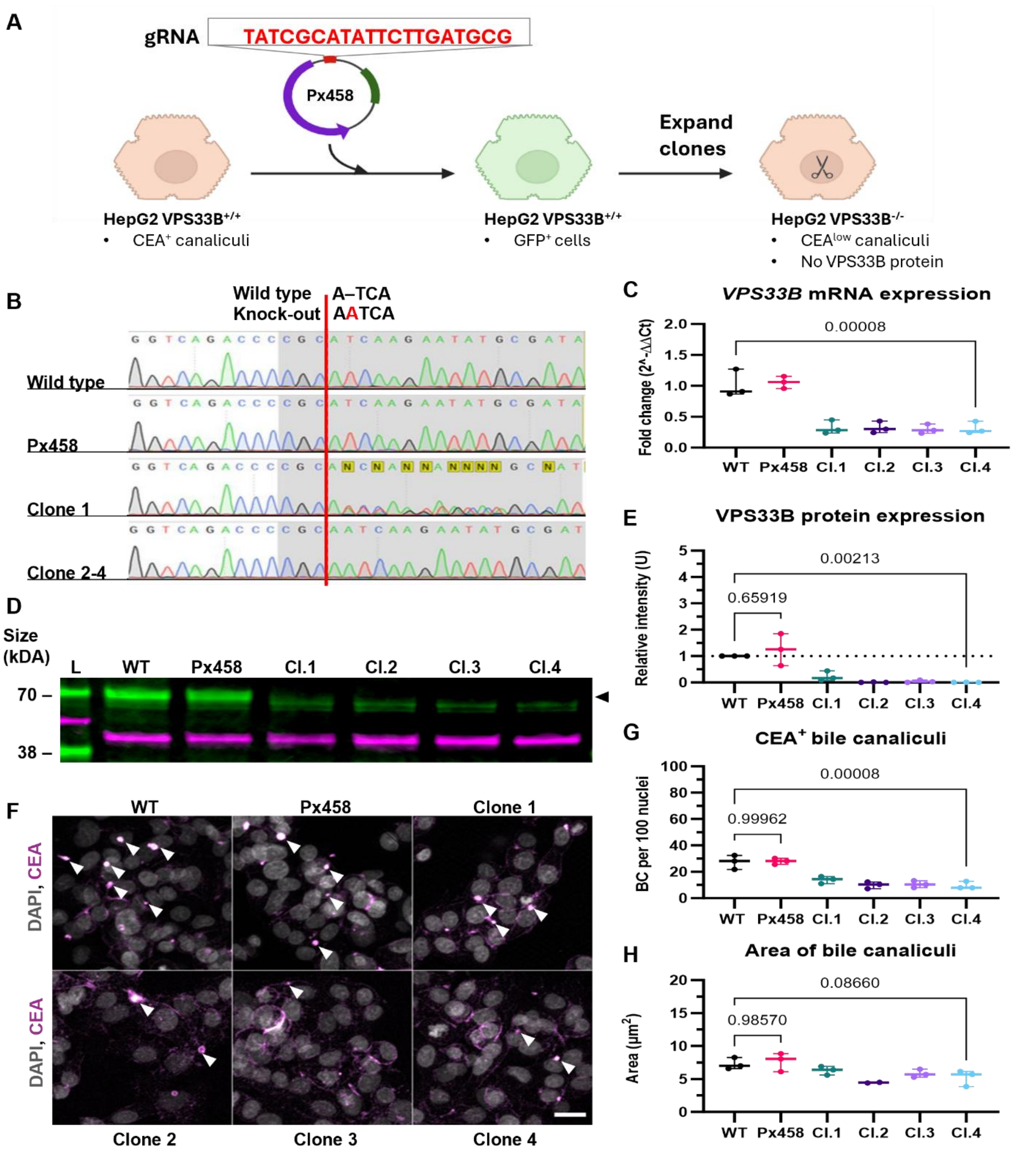

Supplementary Figure 1. CRISPR-Cas9 Knock-out of VPS33B in HepG2 Cells. (A) Schematic of the *VPS33B* knock-out process in HepG2 cells. Wild-type (WT) HepG2 cells (*VPS33B*$^{+/+}$) were transfected with the Px458 plasmid, expressing Cas9, gRNA (5’-

TATCGCATATTCTTGATGCG-3') targeting *VPS33B* exon 4, and *GFP*. GFP-positive cells were sorted at 48 hours, expanded, and single-cell clones were generated by serial dilution. Clones were assessed for *VPS33B* RNA and protein expression and carcinoembryonic antigen (CEA) localisation at bile canaliculi. **(B)** Sanger sequencing traces of the cutting site associated with the (5'-TATCGCATATTCTTG ATGCG-3') gRNA. Clones 2-4 have an adenine addition at the cutting site, which generates a frame shift. **(C)** RT-qPCR analysis of *VPS33B* mRNA in WT, Px458, and clones (Cl.1-4), normalised to *MDH1* and compared to WT expression levels (n=3 independent replicates). **(D)** Western blot of VPS33B protein in WT, Px458, and Cl.1-4. VPS33B (green, black arrow) and β-actin (magenta) fluorescence were quantified and normalised to the WT protein level. **(E)** Quantification of western blot VPS33B relative fluorescence intensity compared to β-actin and normalised to wild-type levels (n=3 immunoblots). Cell polarisation was assessed by quantification of $CEA^{positive}$ canaliculi (magenta), enhanced by Oncostatin. **(F)** Representative confocal images taken at 20x magnification with white arrows indicating bile canaliculi (Scale bars measure 100µm). **(G)** Quantification of $CEA^{positive}$ bile canaliculi per 100 nuclei. **(H)** Measurement of the area covered by the $CEA^{positive}$ bile canaliculi. Graphs show individual values, medians, and IQRs. Statistical analysis used one-way ANOVA with Tukey's test ($p < 0.05$ was considered significant). Source data are provided as a Source Data file.

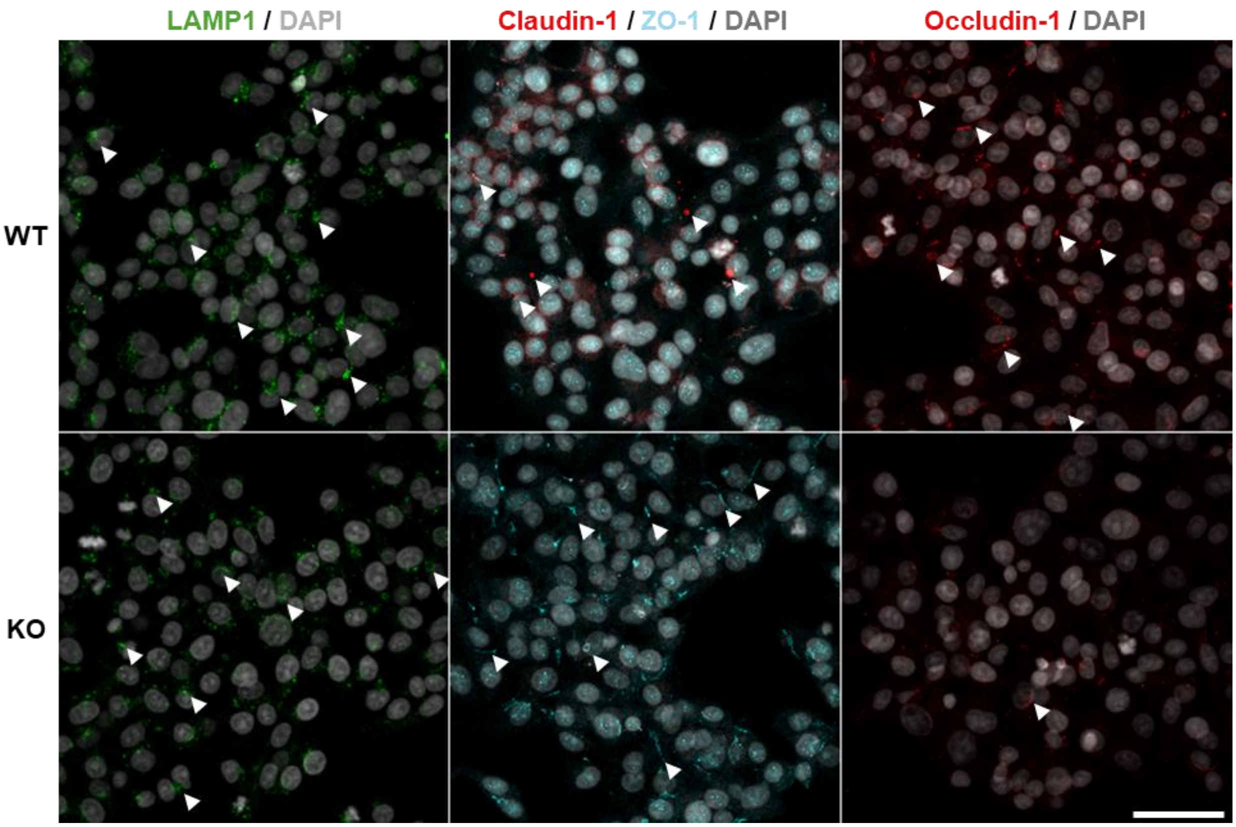

Supplementary Figure 2. Further characterisation of Clone 4 VPS33B$^{-/-}$ HepG2 cells. VPS33B$^{-/-}$ HepG2 (KO) cells were cultured alongside wild-type (WT) HepG2 cells in ibidi plates in the presence of oncostatin M to promote the formation of bile canaliculi-like structures. Cells were subsequently immunostained for LAMP1 (green), Claudin-1 (red), ZO-1 (cyan), and Occludin-1 (red). Representative confocal images were acquired at 40× magnification (scale bar measures 100 μm), with white arrows indicating regions of interest. Notably, VPS33B$^{-/-}$ cells exhibited reduced staining intensity of LAMP1, Claudin-1, and Occludin-1 compared to WT cells. In contrast, ZO-1 appeared more prominently localised at cell–cell junctions in KO cells, forming more continuous and pronounced junctional structures.

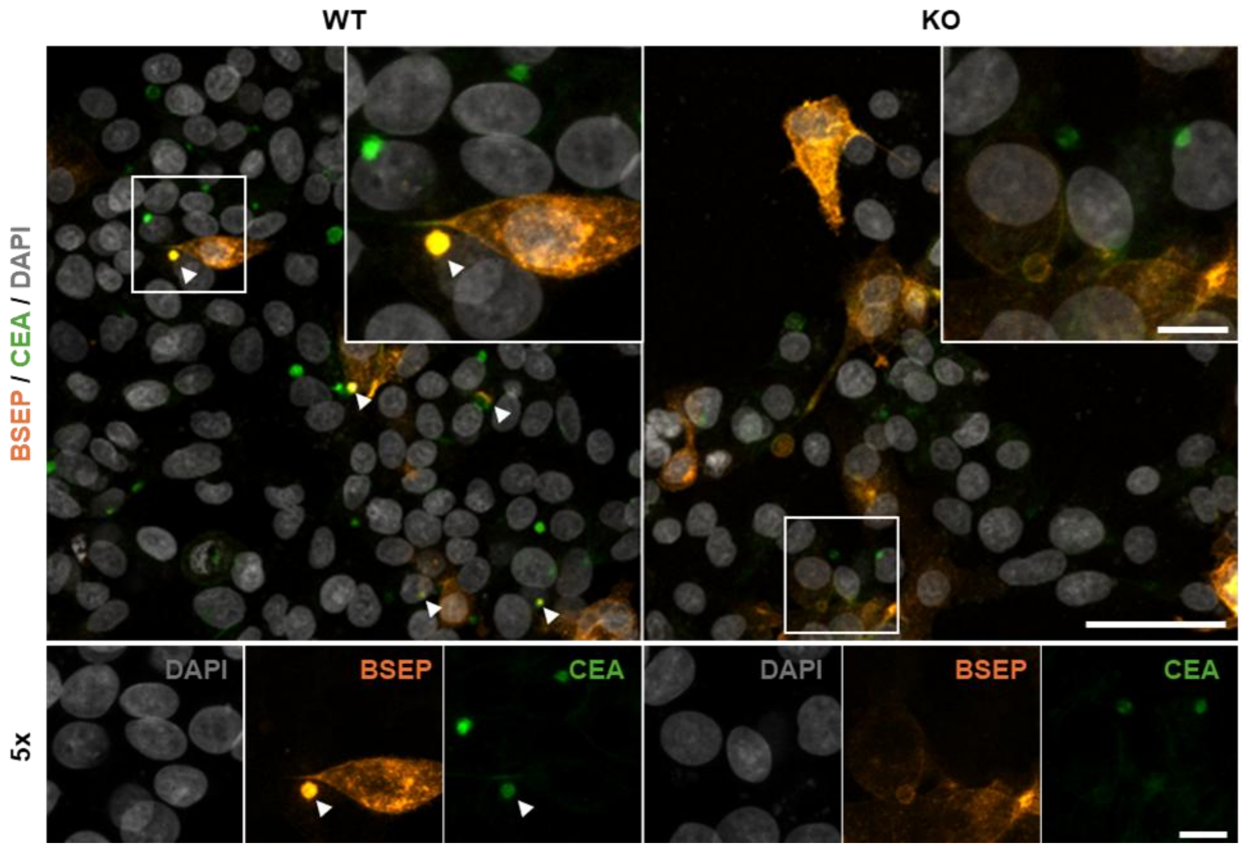

Supplementary Figure 3. Bile salt export pump (BSEP) fails to polarise to bile canaliculi in VPS33B$^{-/-}$ HepG2 cells. VPS33B knockout (KO) HepG2 cells were cultured alongside wild-type (WT) HepG2 cells in ibidi plates. As HepG2 cells express minimal endogenous BSEP, both WT and KO cells were transfected with a BSEP expression plasmid before induction of bile canaliculi formation using oncostatin M. Cells were subsequently immunofluorescently stained for CEA (green), a bile canaliculi marker, and BSEP (orange). Representative confocal images were acquired at 40× magnification (scale bar measures 100 µm). White arrows indicate bile canaliculi structures positive for both CEA and BSEP. Insets show a 5× electronic zoom of the regions highlighted by white boxes, and the lower panels display the individual fluorescence channels corresponding to these insets. Under these conditions, BSEP was correctly polarised to bile canalicular structures in WT cells, whereas VPS33B$^{-/-}$ cells failed to show canalicular localisation of BSEP.

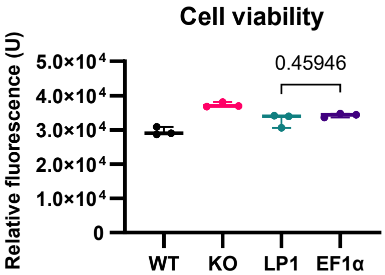

Supplementary Figure 4. The EF1-VPS vector treatment showed similar cell viability to the LP1-VPS treatment in HepG2 *VPS33B*$^{-/-}$ cells. *In vitro* safety was assessed via PrestoBlue assay in n=3 samples of wild type (WT), HepG2 *VPS33B*$^{-/-}$ (KO) cells and KO cells treated with either LP1-VPS (LP1) or EF1-VPS (EF1α) vectors MOI 10. Of note, the vectors used for this experiment were titred in Hek293T cells, so the VCN measured in HepG2 cells was about 4 times higher than the MOI. Graphs show individual values, medians, and IQRs. Statistical analysis used one-way ANOVA with Tukey's test ($p < 0.05$ was considered significant). Source data are provided as a Source Data file.

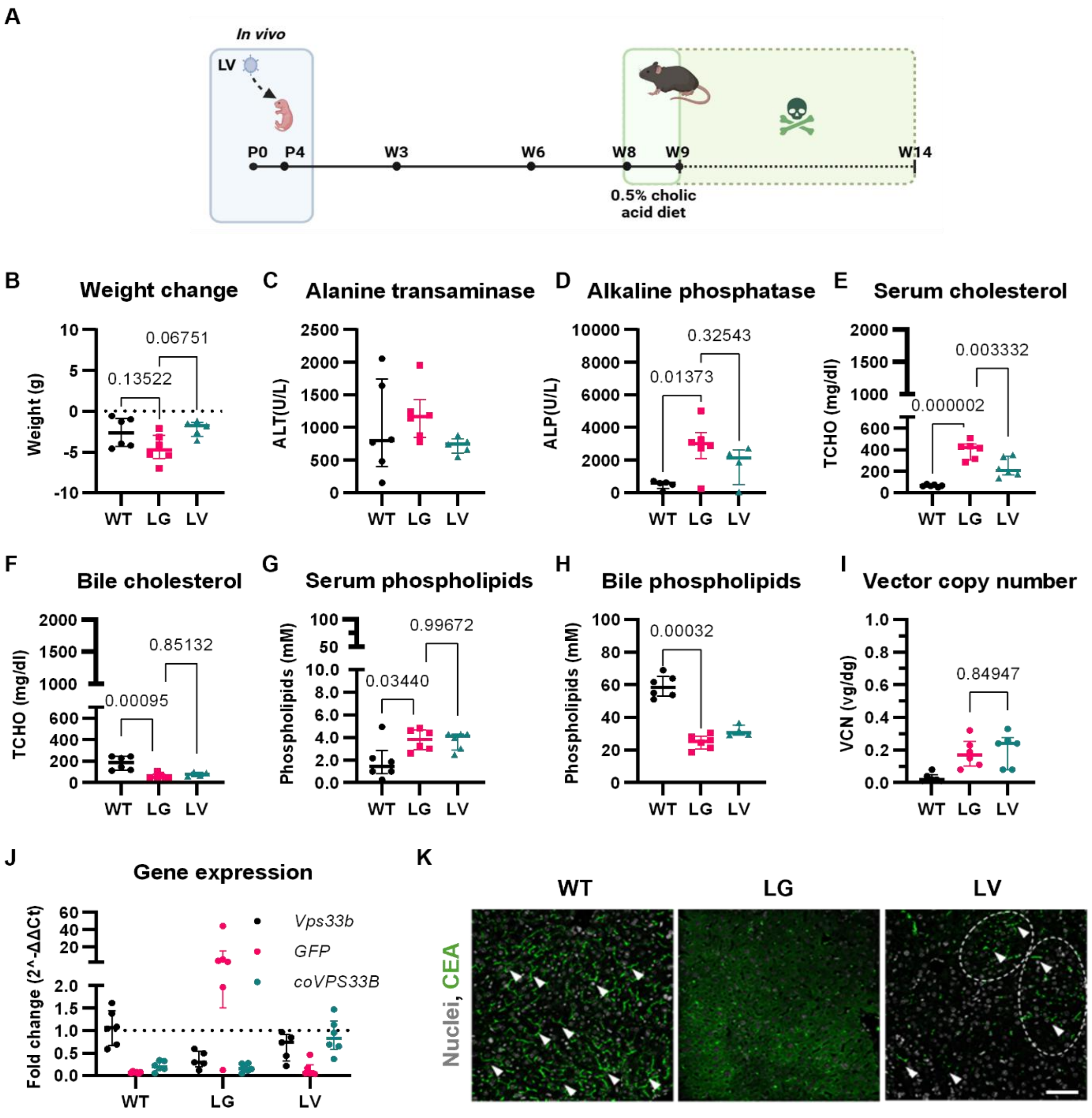

Supplementary Figure 5. Initial LP1-VPS *in vivo* efficacy test. (A) Schematic representation of the experimental plan: Neonatal *Vps33b*$^{Liver-/-}$ mice were injected with LP1-GFP (LG, n=6) or LP1-VPS (LV, n=6) at 5×10^{10} TU/kg. At 8 weeks, mice and wild-type (WT, n=6) controls were placed on a 0.5% cholic acid diet for 6 weeks to exacerbate the cholestatic phenotype. Mice were sacrificed after 1 week due to severe phenotype, earlier than expected based on prior studies. The green area, indicated by a skull symbol, represents the originally planned time period before animal sacrifice. **(B)** Weight changes during 1-week cholic acid administration for WT (n=6) and LG

(n=6) and LV (n=5) mice. Serum measurements of alanine aminotransferase for WT (n=6) and LG (n=6) and LV (n=5) mice **(C)**, alkaline phosphatase for WT (n=6) and LG (n=6) and LV (n=4) mice **(D)**, total cholesterol (n=6 for all groups) **(E)** and phospholipids for WT (n=6) and LG (n=6) and LV (n=4) mice **(G)**. Bile samples analysis of total cholesterol (n=6 for all groups) **(F)** and phospholipids for WT (n=6) and LG (n=6) and LV (n=4) mice **(H)**. **(I)** Vector copy number in liver tissue (qPCR, n=6 for all groups). **(J)** Expression of *wtVps33b* for WT (n=6) and LG (n=5) and LV (n=5) mice, *coVPS33B* (n=6 for all groups), and *GFP* (n=6 for all groups) compared to *Hprt* and normalised to average WT *wtVps33b* levels (qPCR). **(K)** Immunofluorescence microscopy images acquired at 25× magnification showing CEA (green) and nuclei (grey) in liver sections. White arrows point to $CEA^{positive}$ bile canaliculi; dashed circles indicate $CEA^{positive}$ bile canaliculi areas in treated samples (scale bar measures 100 μm). Graphs display individual values, medians, and IQRs. Statistical analysis used one-way ANOVA with Tukey's test ($p<0.05$ considered significant). Source data are provided as a Source Data file.

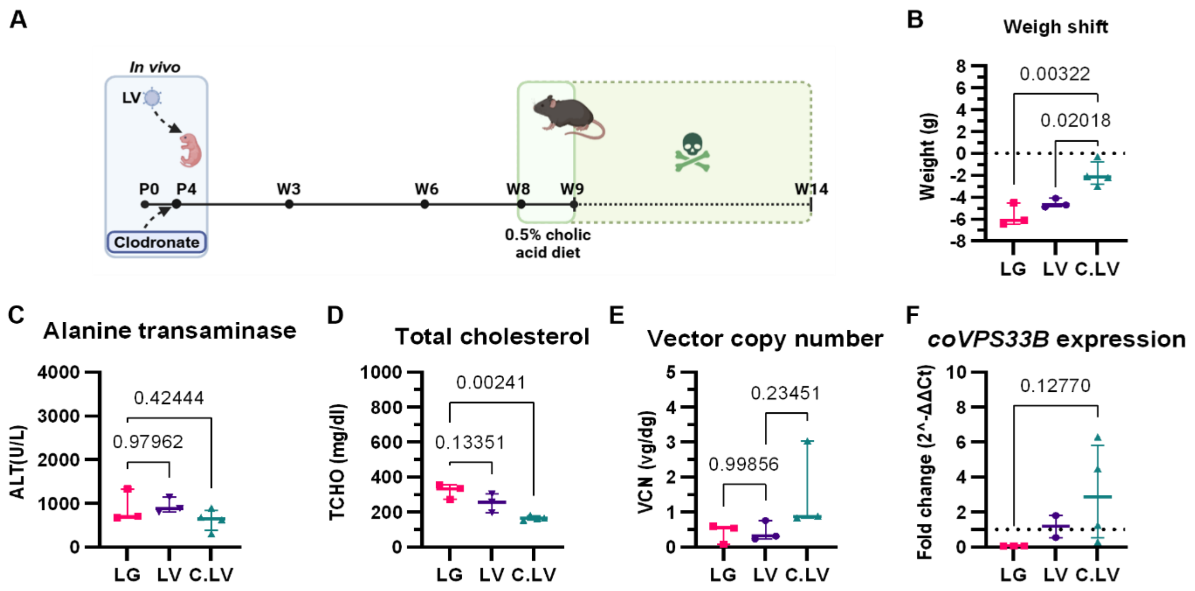

Supplementary Figure 6. Clodronate liposomes enhance lentiviral gene therapy for ARC syndrome. (A) Schematic representation of the experimental plan: Neonatal *Vps33b*$^{Liver-/-}$ mice were pre-treated with 0.12 g/kg clodronate liposomes via intraperitoneal injections 24 and 6 hours before lentiviral vector delivery. At P3-4, clodronate-treated mice received 5×10^{10} TU/kg LP1-VPS (C.LV, n=4). Control groups included LP1-GFP (LG, n=3) and LP1-VPS (LV, n=3) without clodronate pre-treatment. At 8 weeks, all mice, along with wild-type (WT, n=6), were placed on a 0.5% cholic acid diet for 6 weeks to exacerbate the cholestatic phenotype. Mice were sacrificed after one week due to severe symptoms. The green area, indicated by a skull symbol, represents the originally planned time period before animal sacrifice. **(B)** Weight change after 1 week on the cholic acid diet for LG (n=3), LV (n=3) and C.LV (n=4). Serum alanine transaminase **(C)** and cholesterol levels **(D)** measured at harvest for LG (n=3), LV (n=3) and C.LV (n=4). **(E)** Vector copy number (qPCR, n=3 for all groups). **(F)** Expression of *coVPS33B* compared to *Hprt* and normalised to average WT *wtVps33b* levels (qPCR) for LG (n=3), LV (n=2) and C.LV (n=4). Graphs display individual values, medians, and IQRs. Statistical analysis used one-way ANOVA with Tukey's test ($p<0.05$ considered significant). Source data are provided as a Source Data file.

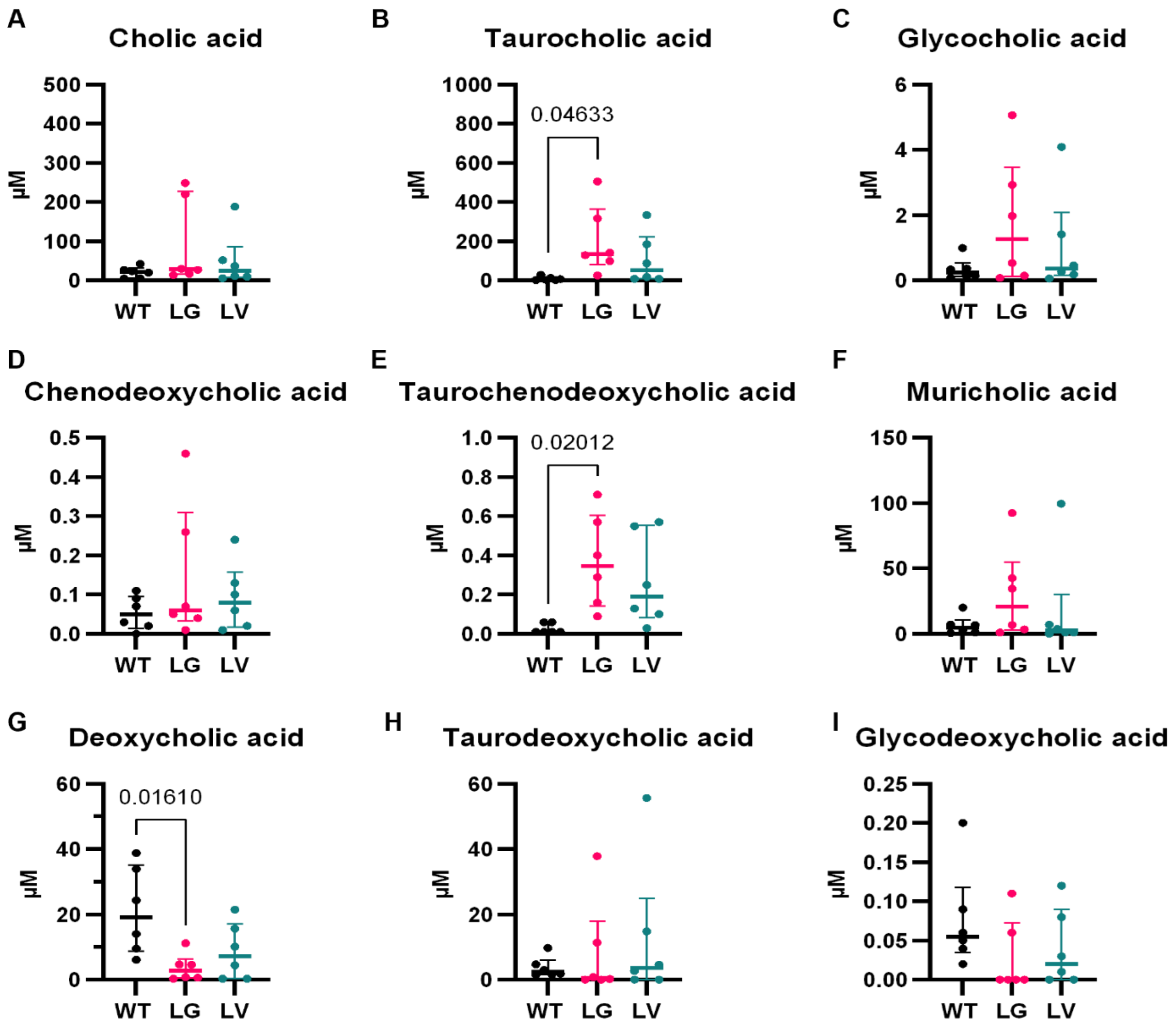

Supplementary Figure 7. Bile acids measurements. Neonatal *Vps33b*$^{Liver-/-}$ mice were pre-conditioned with 0.12 g/kg clodronate liposomes 24 and 6 hours before lentiviral vector delivery. At P3-4, they received $5x10^{10}$ TU/kg of LP1-GFP (LG, n=6) or LP1-VPS (LV, n=6). At 4 weeks, treated mice and wild-type (WT, n=6) were placed on a 0.25% cholic acid diet to exacerbate the cholestatic phenotype. Mice were sacrificed at 12 weeks and dry blood spots were collected for bile acid analysis by mass spectrometry. The graphs display individual measurement values (n=6 for all groups), medians and IQRs of cholic acid (**A**), taurocholic acid (**B**), glycocholic acid (**C**), chenodeoxycholic acid (**D**), taurochenodeoxycholic acid (**E**), muricholic acid (**F**), deoxycholic acid (**G**), taurodeoxycholic acid (**H**) and glycodeoxycholic acid (**I**). Statistical analysis used one-way ANOVA with Tukey's test ($p<0.05$ considered significant).

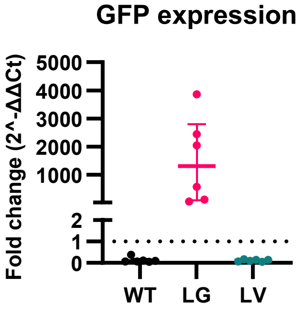

Supplementary Figure 8. LP1-GFP vectors drive GFP expression in the livers of control animals. Experimental plan: Neonate *Vps33b*$^{Liver\text{-}/\text{-}}$mice received 0.12 g/kg clodronate liposomes intraperitoneally 24 and 6 hours before 5x10^{10} TU/kg intravenous injection of LP1-*GFP* (LG, n=6) or LP1-*VPS* (LV, n=6) at P3-4. At 4 weeks of age, they were placed on a 0.25% cholic acid diet to induce cholestasis along with wild-type (WT, n=6) mice. Expression of GFP compared to Hprt and normalised to average WT wtVps33b levels (qPCR). The graph shows individual values, medians, and IQRs. Source data are provided as a Source Data file.

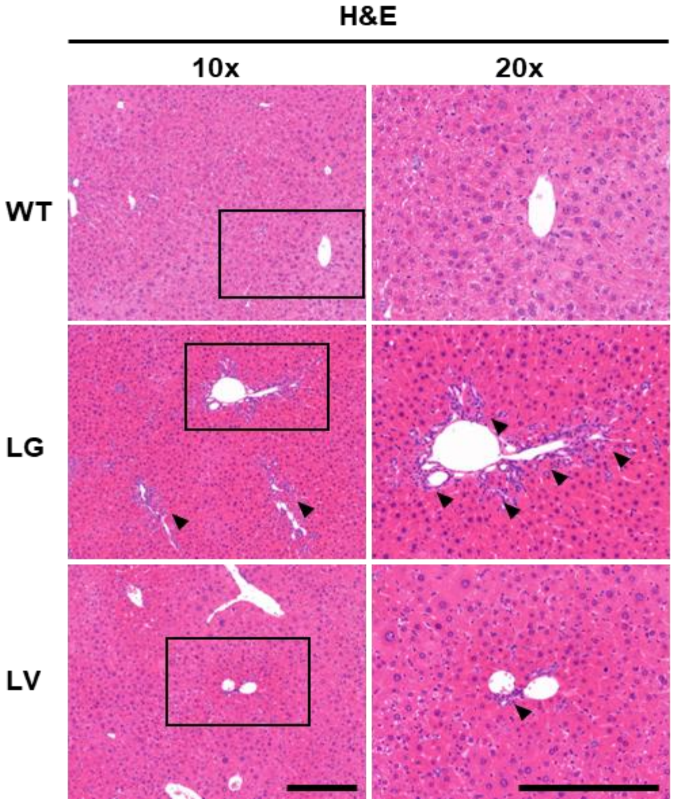

Supplementary Figure 9. H&E staining reveals reduced immune cell infiltrates post LP1-VPS treatment. Neonatal *Vps33b*$^{Liver-/-}$ mice were pre-conditioned with 0.12 g/kg clodronate liposomes 24 and 6 hours before lentiviral vector delivery. At P3-4, they received 5x10^{10} TU/kg of LP1-GFP (LG, n=6) or LP1-VPS (LV, n=6). At 4 weeks, treated mice and wild-type (WT, n=6) were placed on a 0.25% cholic acid diet to exacerbate the cholestatic phenotype. At 12 weeks, the animals were sacrificed and their livers harvested. Representative images of H&E staining showing infiltrates in the WT, LG and LV samples. Black arrows point towards cellular infiltrates, while the black squares in the 10x magnification images mark the region presented at 20x magnification. The scale bars measure 250 µm.

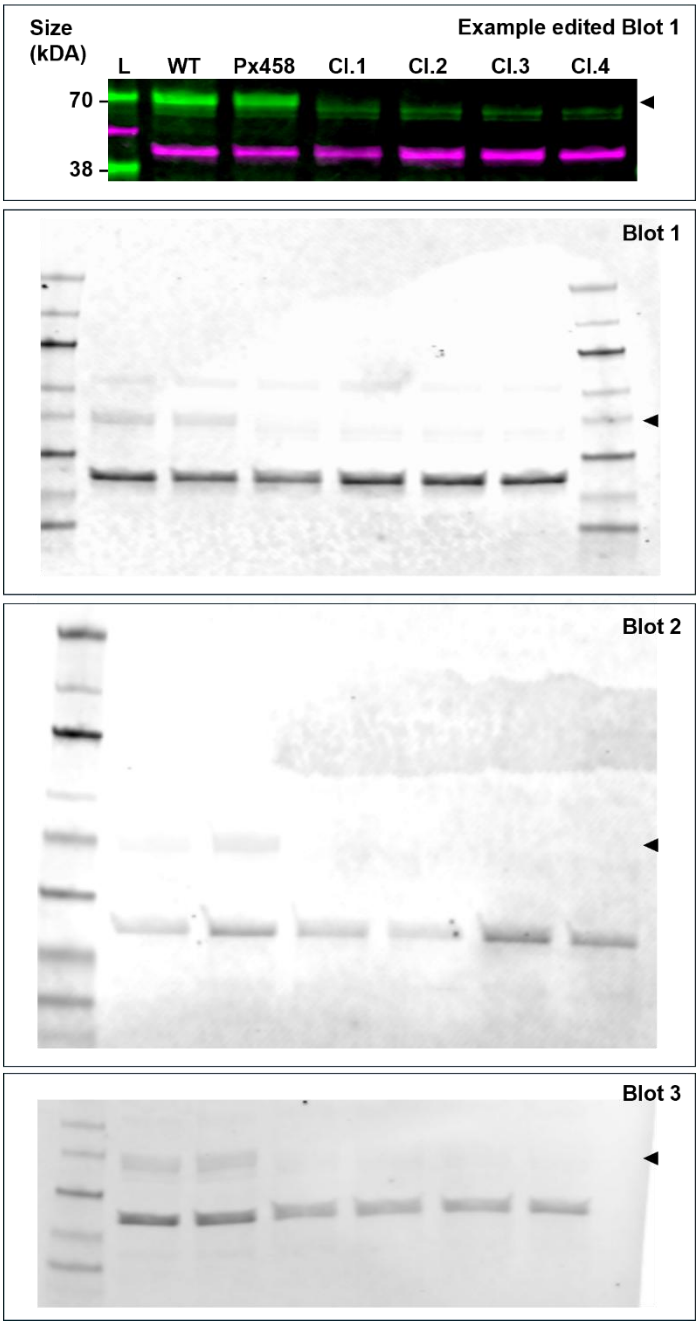

Supplementary Figure 10: Uncropped western blots used for Supplementary Figure 1.E analysis.

Supplementary Table 1. Integration sites with top 5 frequencies. Top integration sites determined by IS analysis of each of the cancer samples (C1-3). The number after the sample code represents the region biopsied. C1 and C2 tumours were localised and collected from the left lateral lobe, while C3.1 and C3.2 were found on the right medial lobe and the right lateral lobe of the liver. Source data are provided as a Source Data file.

Sample	Chromosome	Start	End	Strand	Frequency	Gene Symbol
C1.1	chr3	144516233	144516255	Positive	25.170	*Hs2st1*
	chr3	79600196	79600217	Positive	16.122	*Ppid*
	chr4	57507860	57507868	Negative	12.993	*ND*
	chr8	42181115	42181124	Negative	8.163	*2810404M03Rik*
	chr9	88687372	88687376	Negative	4.422	*Mthfsl*
C1.2	chr3	144516233	144516266	Positive	36.197	*Hs2st1*
	chr3	79600197	79600217	Positive	18.015	*Ppid*
	chr4	57507859	57507868	Negative	16.430	*ND*
	chr8	42181115	42181121	Negative	8.507	*2810404M03Rik*
	chr9	88687372	88687376	Negative	4.504	*Mthfsl*
C1.3	chr3	144516233	144516269	Positive	34.351	*Hs2st1*
	chr3	79600197	79600217	Positive	20.000	*Ppid*
	chr4	57507861	57507866	Negative	15.649	*ND*
	chr8	42181116	42181121	Negative	9.542	*2810404M03Rik*
	chr9	88687372	88687379	Negative	5.038	*Mthfsl*
C2	chr19	15981125	15981139	Positive	2.184	*Cep78*
	chr13	29660762	29660767	Negative	1.921	*Cdkal1*
	chr4	97763827	97763846	Positive	1.890	*E130114P18Rik*
	chrX	42217413	42217421	Negative	1.750	*Stag2*
	chr4	22434439	22434444	Negative	1.642	*Pou3f2*
C3.1	chr2	91274277	91274300	Positive	17.384	*Arfgap2*
	chr9	124260169	124260191	Positive	8.931	*2010315B03Rik*
	chr9	4308155	4308170	Positive	6.911	*Aasdhppt*
	chr13	21556263	21556287	Positive	6.699	*Olfr1535*
	chr11	23343971	23343987	Positive	6.592	*Usp34*
C3.2	chr15	22127702	22127725	Positive	8.463	*Cdh18*
	chr1	3551468	3551494	Positive	8.013	*Xkr4*
	chr11	3142737	3142754	Positive	7.931	*Sfi1*
	chr1	146436552	146436576	Positive	7.809	*Brinp3*
	chr12	34999497	34999518	Positive	7.318	*Prps1l1*

Supplementary Table 2. Top 10 most significantly up- and downregulated genes. The p-values were estimated using the Wald test and adjusted p-values using the Benjamini-Hochberg (BH) method. Source data are provided as a Source Data file.

Change	Gene Symbol	baseMean	Healthy	Tumour	log2FoldChange	padj
Up	5330417C22Rik	3086.36	70	5097	6.09	6.99E-109
	Mfge8	1140.38	129	1815	3.784	1.04E-83
	Abi2	592.22	159	881	2.453	8.11E-77
	Wbp5	1088.25	269	1634	2.579	5.12E-48
	Fam101b	289.84	38	458	3.535	1.19E-46
	Pak1	476.83	84	739	3.109	1.30E-44
	Ly6c1	173.29	9	283	4.92	1.30E-44
	Pygb	1427.39	176	2262	3.628	1.44E-43
	Mansc1	177.37	45	265	2.524	1.48E-42
	Cd93	2218.56	327	3479	3.365	2.82E-42
Down	Srd5a1	973.71	2146	192	-3.451	1.14E-68
	Fcna	472.49	1137	30	-5.148	6.58E-49
	Adap2	680.21	1206	329	-1.847	1.84E-48
	Ugt1a9	748.95	1850	15	-6.591	3.72E-48
	Aldh1a1	13899.28	30062	3124	-3.232	4.40E-48
	Cyp2c54	1087.49	2645	49	-5.593	1.12E-44
	Mmd2	320.71	793	6	-6.651	1.18E-44
	Oat	15202.9	37534	316	-6.63	1.85E-43
	Hsd17b11	1795.76	3175	876	-1.839	1.12E-41
	Prodh	2018.64	4702	230	-4.275	1.78E-40

Supplementary Table 3. Sequence based reagents list

Name	Sequence	Supplier
*VPS33B*_E22-23F	GCAGAGAGAAAGGCTACAGGT	Integrated DNA Technologies
*VPS33B*_E22-23R	TGAGGAATGTGTTCAGGGAAG	Integrated DNA Technologies
*coVPS33B*_F	TCTGGGAAGAGAAAAGGGGTAT	Integrated DNA Technologies
*coVPS33B*_R	TCACTTCGGACATTGCTTCC	Integrated DNA Technologies
*Vps33b*_L_F	GTGAGCTTCCCGACTTCTCT	Integrated DNA Technologies
*Vps33b*_L_R	CCACTTCATGTTGCTTCAGGA	Integrated DNA Technologies
*GFP*_F	GGCACAAGCTGGAGTACAAC	Integrated DNA Technologies
*GFP*_R	AGTTCACCTTGATGCCGTTC	Integrated DNA Technologies
*MDH1*_F	GTCACGACTGTGCAGCAGCGT	Integrated DNA Technologies
*MDH1*_R	TGGGGTTCCAAACCAGATGTCCCTG	Integrated DNA Technologies
*Hprt1*_F	CCCTGGTTAAGCAGTACAGC	Integrated DNA Technologies
*Hprt1*_R	ACAAAGTCTGGCCTGTATCCA	Integrated DNA Technologies
R-U5-PBS F	AGCTTGCCTTGAGTGCTT	Integrated DNA Technologies
R-U5-PBS R	GCGCCACTGCTAGAGATTT	Integrated DNA Technologies
β-actin F	GTAGCACAGCTTCTCCTTAAT	Integrated DNA Technologies
β-actin R	GGACCTGACTGACTACCT	Integrated DNA Technologies
Titin F	AAAACGAGCAGTGACGTGAGC	Integrated DNA Technologies
Titin R	TTCAGTCATGCTGCTAGCGC	Integrated DNA Technologies
*Vps33b*_Flox_F	CCAACTGAATTGCTTGCTCAGC	Integrated DNA Technologies
*Vps33b*_Flox_R	GTGTGACTCAGTGATACAGC	Integrated DNA Technologies
*Cre*_F	CCCGCAGAACCTGAAGATGTTC	Integrated DNA Technologies
*Cre*_R	CCTGATCCTGGCAATTTCGGC	Integrated DNA Technologies
β-actin probe	56-FAM-CGCGCTCGGTGAGGATCTT CAT/3BHQ_1	Integrated DNA Technologies
Titin probe	56-FAM-CGCGCTCGGTGAGGATCTT CAT/3BHQ_1	Integrated DNA Technologies
LTR probe	56-FAM-CAGTGGCGCCCGAACAG GGA/3BHQ_1-	Integrated DNA Technologies
HIV-Psi Forward	CAGGACTCGGCTTGCTGAAG	Integrated DNA Technologies
HIV-Psi Reverse	TCCCCCGCTTAATACTGACG	Integrated DNA Technologies
HIV-Psi Probe	FAM-CGCACGGCAAGAGGCGAGG	Integrated DNA Technologies
Titin ddPCR probe	HEX-TGCACGGAAGCGTCTCGTCTC AGTC	Integrated DNA Technologies

Supplementary Table 4. Other reagents list (e.g. drugs, proteins, vectors etc.)

Name	Manufacturer	Cat no.
5053 with 0.1% sodium cholate light blue	Testdiet	T-5ZP4-lightblue
5053 with 0.25% sodium cholate green	Testdiet	T-5ZP5-green
5053 with 0.05% sodium cholate yellow	Testdiet	T-5ZPT-yellow
Antifade Mounting Medium with DAPI	VECTASHIELD	H-1200-10
Bovine Serum Albumin	Sigma-Aldrich	A9418
Chameleon Duo ladder	Li-COR Biosciences	928-60000
Citrate buffer	Sigma-Aldrich	C9999
Clodronate liposomes	Tribioscience	F70101c-nh
Dneasy Blood & Tissue Kit	Qiagen	69504
DPBS (1x) without Ca and Mg	Gibco	14190-094
DPX resin	Militonadams	10197905000
Dulbecco's-modified Eagle's medium	Gibco	11965-092
Endofree Plasmid Maxi Kit (10)	Qiagen	12362
Eosin	Pioneer research chemicals	PRC/66/1
Ethanol	Sigma-Aldrich	32221-2.5L-M
Faetal calf serum	Sigma	F7524
Fetal bovine serum	Gemini bio	100-106
Formaldehyde 36% solution	Sigma-Aldrich	FX0415
Harris haematoxylin	Pioneer research chemicals	Prc/r/51
Hematoxylin counterstain	Vectorlabs	H-3401-500
High-capacity cDNA RT kit	Life Technologies	4368813
Histo-clear II	National Diagnostics	HS-202
Isopropanol	Sigma-Aldrich	59300-2.5L
Methanol	Sigma-Aldrich	34885-2.5L-M
Micro sample tube serum CAT-Gel	Sarstedt	41.1378.005
Neutral buffered formalin	Cellpath	Baf-6000-08a
Normal goat serum	Sigma-Aldrich	NS02L
Nupage Antioxidant	Thermofishe	NP0005
Nupage MOPS SDS Running Buffer	Thermofisher	NP0001
Nupage™ 4 to 12%, Bis-Tris gel	Invitrogen™	NP0322BOX
Oncostatin	Sigma-Aldrich	O9635
Penicillin-streptomycin	Gibco	10378-016
Phalloidin–Alexa 488	Thermo fisher scientific	A12379
Phospholipid assay kit	Merck	MAK122-1KT
Picrosirius red stain kit	Abcam	Ab150681
Pierce™ BCA protein assay kit	Thermo	23225
Powder milk	M7409	Sigma-Aldrich
Prestoblue assay	Thermofisher	A13262
Protease and Phosphatase Inhibitor	Thermo	78443
Qiaprep Spin Miniprep Kit (50)	Qiagen	27104
Qiaquick Gel Extraction Kit (50)	Qiagen	28704
RIPA lysis buffer	Teknova	R3792
Rneasy Mini Kit	Qiagen	74034
Takyon ROX Probe qPCR kit	Eurogentec	UF-RPMT-C0701
Trans-Blot Turbo Mini 0.2 µm PVDF Transfer Packs	Bio-Rad	1704156
Triton™X-100	Sigma	T9284
Trypsin EDTA (0.05%)	Gibco	25300054
Trypsin-EDTA (0.25%)	Gibco	25200056
Vibrance Antifade Mounting Medium	VECTASHIELD	H-1700
Xylene	VWR	28976.294

Supplementary Table 5. Multiple reaction monitoring transitions for bile acid analysis by mass spectrometry.

Bile acid	Transition (*m/z*)	Cone voltage (V)	Collision energy (V)
Cholic/ Muricholic acid	407.3 > 407.3	112	30
Deoxycholic/ Chenodeoxycholic acid	391.3 > 391.3	110	30
Glycocholic acid	464.3 > 74.0	106	36
Glycodeoxycholic/ glycochenodeoxycholic acid	448.2 > 74.0	104	34
Taurocholic acid	514.2 > 80.0	118	66
Taurodeoxycholic/ Taurochenodeoxycholic acid	498.3 > 80.0	120	60

Supplementary Table 6. Gradient profiles table.

Time (min)	Flow rate (mL/min)	A (%)	B (%)	Gradient
Initial	0.200	97	3	Initial
1.33	0.200	97	3	6
7.7	0.200	65	35	6
8.2	0.200	0	100	6
9.9	0.400	0	100	6
10.91	0.400	97	3	1
11.49	0.400	97	3	1
11.5	0.200	97	3	6
12	0.200	97	3	6

Supplementary Table 7. MRM transitions used for targeted quantification of VPS33B and reference proteins. Multiple reaction monitoring (MRM) transitions were acquired on a Xevo TQ-XS triple quadrupole mass spectrometer. For each peptide, one transition was designated as the quantifier, and additional transitions were monitored as qualifiers for identity confirmation.

Protein	Peptide sequence	Precursor m/z	Product m/z	Cone voltage (V)	Collision energy (eV)	Transition type
VPS33B	IANVSILK	429.276	744.461	35	15	Quantifier
VPS33B	IANVSILK	429.276	673.424	35	15	Qualifier
VPS33B	IANVSILK	429.276	460.313	35	15	Qualifier
VPS33B	RPEIGHIFLLDR	489.281	663.382	35	16	Quantifier
VPS33B	RPEIGHIFLLDR	489.281	776.467	35	16	Qualifier
VPS33B	RPEIGHIFLLDR	489.281	690.368	35	16	Qualifier
VPS33B	SWQGLDEVVR	594.804	787.431	35	21	Quantifier
VPS33B	SWQGLDEVVR	594.804	915.489	35	21	Qualifier
VPS33B	SWQGLDEVVR	594.804	365.708	35	21	Qualifier
ACTB	DLTDYLMK	499.747	770.375	35	17	Quantifier
ACTB	DLTDYLMK	499.747	669.328	35	17	Qualifier
ACTB	DLTDYLMK	499.747	554.301	35	17	Qualifier
PGK1	AAVPSIK	343.216	444.282	35	12	Quantifier
PGK1	AAVPSIK	343.216	543.350	35	12	Qualifier
PGK1	AAVPSIK	343.216	242.150	35	12	Qualifier
GAPDH	QASEGPLK	415.224	414.271	35	14	Quantifier
GAPDH	QASEGPLK	415.224	630.346	35	14	Qualifier
GAPDH	QASEGPLK	415.224	315.676	35	14	Qualifier
PARK7	DGLILTSR	437.753	589.367	35	15	Quantifier
PARK7	DGLILTSR	437.753	476.283	35	15	Qualifier
PARK7	DGLILTSR	437.753	399.224	35	15	Qualifier

Supplementary References

1. Haeussler, M. et al. Evaluation of off-target and on-target scoring algorithms and integration into the guide RNA selection tool CRISPOR. Genome Biol. 17, 148 (2016). https://doi.org/10.1186/s13059-016-1012-2
