# Supplementary material for: Safety and efficacy analysis of in vivo lentiviral gene therapy in pre-clinical ARC syndrome models
Source: Nat Commun. 2026 Jun 19;17:5074. doi: 10.1038/s41467-026-73631-x (PMC13282399; doi:10.1038/s41467-026-73631-x)
Supplement: Supplementary file 2 — Reporting Summary [file 41467_2026_73631_MOESM2_ESM.pdf]

nature portfolio

Corresponding author(s): Claudiu A. Cozmescu
Paul Gissen

Last updated by author(s): May 6, 2026

Reporting Summary

Nature Portfolio wishes to improve the reproducibility of the work that we publish. This form provides structure for consistency and transparency in reporting. For further information on Nature Portfolio policies, see our Editorial Policies and the Editorial Policy Checklist.

Statistics

For all statistical analyses, confirm that the following items are present in the figure legend, table legend, main text, or Methods section.

n/a	Confirmed	
☐	☒	The exact sample size (n) for each experimental group/condition, given as a discrete number and unit of measurement
☒	☐	A statement on whether measurements were taken from distinct samples or whether the same sample was measured repeatedly
☐	☒	The statistical test(s) used AND whether they are one- or two-sided<br>*Only common tests should be described solely by name; describe more complex techniques in the Methods section.*
☐	☒	A description of all covariates tested
☐	☒	A description of any assumptions or corrections, such as tests of normality and adjustment for multiple comparisons
☐	☒	A full description of the statistical parameters including central tendency (e.g. means) or other basic estimates (e.g. regression coefficient) AND variation (e.g. standard deviation) or associated estimates of uncertainty (e.g. confidence intervals)
☐	☒	For null hypothesis testing, the test statistic (e.g. F, t, r) with confidence intervals, effect sizes, degrees of freedom and P value noted<br>*Give P values as exact values whenever suitable.*
☒	☐	For Bayesian analysis, information on the choice of priors and Markov chain Monte Carlo settings
☒	☐	For hierarchical and complex designs, identification of the appropriate level for tests and full reporting of outcomes
☐	☒	Estimates of effect sizes (e.g. Cohen's d, Pearson's r), indicating how they were calculated

Our web collection on statistics for biologists contains articles on many of the points above.

Software and code

Policy information about availability of computer code

Data collection

Software - Name - Manufacturer Version
> Zen black 2009 - Zeiss - V2.3

Data analysis

Software Name - Process - Version
> ImageJ - ImageJ - V2.3.0
> GraphPad - PRISM - GraphPad PRISM V10.0.2
> SnapGene - SnapGene - V8.0
> Python, yaml - CUSTOM_DUMPSOFTWAREVERSIONS V3.11.0, V6.0
> Getchromsizes - CUSTOM_GETCHROMSIZES V1.16.1
> Fastqc - FASTQC V0.11.9
> Fq - FQ_SUBSAMPLE V0.9.1
> Python - GTF_GENE_FILTER, SALMON_TX2GENE, SAMPLESHEET_CHECK V3.9.5
> Rsem, star - MAKE_TRANSCRIPTS_FASTA V1.3.1, V2.7.10a
> Salmon - SALMON_INDEX, SALMON_QUANT V1.10.1
> Bioconductor-summarizedexperiment, r-base - SALMON_SE_GENE V1.24.0, 4.1.1
> Bioconductor-tximeta, r-base - SALMON_TXIMPORT V1.12.0, 4.1.1
> Samtools - SAMTOOLS_FLAGSTAT, SAMTOOLS_IDXSTATS, SAMTOOLS_INDEX, SAMTOOLS_SORT, SAMTOOLS_STATS - V1.17
> Samtools, gawk, star STAR_ALIGN_IGENOMES V1.10, V5.1.0, V2.6.1d
> Cutadapt, trimgalore TRIMGALORE V3.4, V0.6.7

> Umitools UMITOOLS_DEDUP, UMITOOLS_PREPAREFORSALMON V1.1.4
> Nextflow, nf-core/rnaseq Workflow V22.10.7, V3.12.0
> Bile canaliculi analysis maro script: https://github.com/DaleMoulding/Fiji-Macros/blob/master/README.md#bile-canaliculi-assay

For manuscripts utilizing custom algorithms or software that are central to the research but not yet described in published literature, software must be made available to editors and reviewers. We strongly encourage code deposition in a community repository (e.g. GitHub). See the Nature Portfolio guidelines for submitting code & software for further information.

Data

Policy information about availability of data

All manuscripts must include a data availability statement. This statement should provide the following information, where applicable:

- Accession codes, unique identifiers, or web links for publicly available datasets
- A description of any restrictions on data availability
- For clinical datasets or third party data, please ensure that the statement adheres to our policy

All data associated with this study are included in the paper or the Supplementary Materials. Source data are provided with this paper. The integration site analysis data used in this study is available in the BioProject database under accession code PRJNA1458313 [http://www.ncbi.nlm.nih.gov/bioproject/1458313]. RNA-seq data discussed here has been deposited in NCBI's Gene Expression Omnibus 55 and is accessible through GEO Series accession number GSE329589 [https://www.ncbi.nlm.nih.gov/geo/query/acc.cgi?acc=GSE329589]. Protein mass spectrometry data presented in this study was deposited in the ProteomeXchange repository under the accession code PXD077802 [https://panoramaweb.org/ZNP2AN.url]. The mass spectrometry bile acid analysis raw data has been uploaded in the MetaboLights under the accession code MTBLS14408 [https://www.ebi.ac.uk/metabolights/MTBLS14408].

Research involving human participants, their data, or biological material

Policy information about studies with human participants or human data. See also policy information about sex, gender (identity/presentation), and sexual orientation and race, ethnicity and racism.

Reporting on sex and gender	N/A
Reporting on race, ethnicity, or other socially relevant groupings	N/A
Population characteristics	N/A
Recruitment	N/A
Ethics oversight	N/A

Note that full information on the approval of the study protocol must also be provided in the manuscript.

Field-specific reporting

Please select the one below that is the best fit for your research. If you are not sure, read the appropriate sections before making your selection.

☒ Life sciences ☐ Behavioural & social sciences ☐ Ecological, evolutionary & environmental sciences

For a reference copy of the document with all sections, see nature.com/documents/nr-reporting-summary-flat.pdf

Life sciences study design

All studies must disclose on these points even when the disclosure is negative.

Sample size	Sample sizes were deemed sufficient based on previous optimisation experiments performed in our lab. Sample sizes were generally above 3, but in an instance where n = 2 (Fig 6. E) this was only displayed on the graph but not included in statistical analysis.
Data exclusions	All individual data points are displayed on the graphs. No data was excluded from the analysis. If there are any points missing is because of inability to collect the sample (i.e bile).
Replication	In vivo experiments followed the exact same protocol but were performed generally with different batches of lentiviral vectors and mice litters obtained at different time points from different breeding pairs. The data obtained was not different between these experiments.
Randomization	N/A
Blinding	N/A

Reporting for specific materials, systems and methods

We require information from authors about some types of materials, experimental systems and methods used in many studies. Here, indicate whether each material, system or method listed is relevant to your study. If you are not sure if a list item applies to your research, read the appropriate section before selecting a response.

Materials & experimental systems

n/a	Involved in the study	
	x	Antibodies
	x	Eukaryotic cell lines
x		Palaeontology and archaeology
	x	Animals and other organisms
x		Clinical data
x		Dual use research of concern
x		Plants

Methods

n/a	Involved in the study	
x		ChIP-seq
x		Flow cytometry
x		MRI-based neuroimaging

Antibodies

Antibodies used

Name - Supplier - Cat no. - Clone no. - Lot no.
> Goat anti-Mouse IgG (H+L) Secondary Antibody AlexaFluor 488 - Invitrogen - A11029 - Polyclonal
> Goat anti-Rabbit IgG (H+L) Secondary Antibody AlexaFluor 568 - Invitrogen - A11036 - Polyclonal
> Goat anti-Mouse IgG (H+L) Secondary Antibody AlexaFluor 647 - Invitrogen - A21236 - Polyclonal
> Mouse anti-MRP2 antibody - Abcam - ab3373 - M2 III-6 - GR3228397-6
> Polyclonal rabbit anti-CEA - DaKo- discontinued - A0115 - Polyclonal - 00032394
> Polyclonal rabbit anti-VPS33B - Proteintech - 12195-1-AP - Polyclonal - 00067271
> Mouse monoclonal IgG anti b-Actin (C4) - sc-47778 - Santa Cruz Biotechnology - Monoclonal
> Mouse anti-beta-Tubulin antibody - Sigma-Aldrich - T8660 - SDL.3D10 - 103M4830
> Mouse anti-GAPDH antibody - Abcam - ab226408 - 12G11 -
> IRDye® 800CW donkey anti-rabbit IgG - Li-COR Biosciences - 926-32213 - Polyclonal
> IRDye® 680RD donkey anti-mouse IgG - Li-COR Biosciences - 926-68072 - Polyclonal

Validation

Name - Citation - Supplier statement
> Mouse anti-MRP2 antibody - doi: 10.1016/j.crmeth.20 23.100440. - "Mouse Monoclonal MRP2 antibody. Suitable for WB and reacts with Human samples. Cited in 85 publications. Immunogen corresponding to Recombinant Fragment Protein within Human ABCC2 aa 1300 to C-terminus."
> Polyclonal rabbit anti-CEA - doi: 10.1177/0192623318789069. - Discontinued
> Polyclonal rabbit anti-VPS33B - doi: 10.1002/ijc.32429. - "VPS33B Polyclonal Antibody for WB, IHC, IF/ICC, ELISA"; "KO/KO validated"
> Mouse monoclonal IgG anti b-Actin (C4) - Cited in 14948 publications (e.g doi: 10.1529/biophysj.104.045153)- ". Anti-β-actin antibody (C4) is recommended for detecting β-actin in various species, including mouse, rat, human, avian, bovine, canine, porcine, rabbit, Dictyostelium discoideum, and Physarum polycephalum, by western blotting (WB), immunoprecipitation (IP), immunofluorescence (IF), immunohistochemistry with paraffin-embedded sections (IHCP), and enzyme-linked immunosorbent assay (ELISA)."
> Mouse Anti-β-Tubulin III Antibody - - "Monoclonal Anti-β-Tubulin Isotype III specifically recognizes an epitope located on human β-tubulin (isotype III). It cross-reacts with bovine and rat in an immunoblotting technique where it localizes the tubulin band in either a rat brain extract or a bovine brain MAPs extract. "
> Mouse anti-GAPDH antibody - doi: 10.17305/bb.2023.9698. - Tested for human and mouse WB.

Eukaryotic cell lines

Policy information about cell lines and Sex and Gender in Research

Cell line source(s)

Name - Citation Supplier - Cat no. - Passage no.
> HepG2 - ATCC - HB-8065 - < 25
> Lenti-X™ 293T - Clontech - 632180 - < 25

Authentication

> HepG2 - STR profiling
> Lenti-X™ 293T - Not performed

Mycoplasma contamination

Monthly testing performed in the department.

Commonly misidentified lines
(See ICLAC register)

N/A

Animals and other research organisms

Policy information about studies involving animals; ARRIVE guidelines recommended for reporting animal research, and Sex and Gender in Research

Laboratory animals

Name - Citation - Supplier - Strain - Sex - Age
> Vps33bfl/fl-Alfp-Cre - DOI: 10.1016/j.jhep.2017.01.001 - N/A - #550 - male and female - 6-12 weeks
> Vps33bfl/wt-Alfp-Cre - N/A - N/A - #550 - male and female - 36 weeks
> C57BL/6J - https://www.jax.org/strain/000664 - Jackson Laboratory - #000664 - male and female - 6-12 weeks

Wild animals	N/A
Reporting on sex	All in vivo experiments contained both male and female animals. For efficacy studies an equal number of male and females were used for each group and no sex difference was observed.
Field-collected samples	N/A
Ethics oversight	Animal work was conducted in compliance with the UK Home Office regulations under project licence number PP9223137 and personal license number I92863816.

Note that full information on the approval of the study protocol must also be provided in the manuscript.

Plants

Seed stocks	N/A
Novel plant genotypes	N/A
Authentication	N/A
