# Supplementary figures and images for: Safety and efficacy analysis of in vivo lentiviral gene therapy in pre-clinical ARC syndrome models
Source: Nat Commun. 2026 Jun 19;17:5074. doi: 10.1038/s41467-026-73631-x (PMC13282399; doi:10.1038/s41467-026-73631-x)

Figure 1F

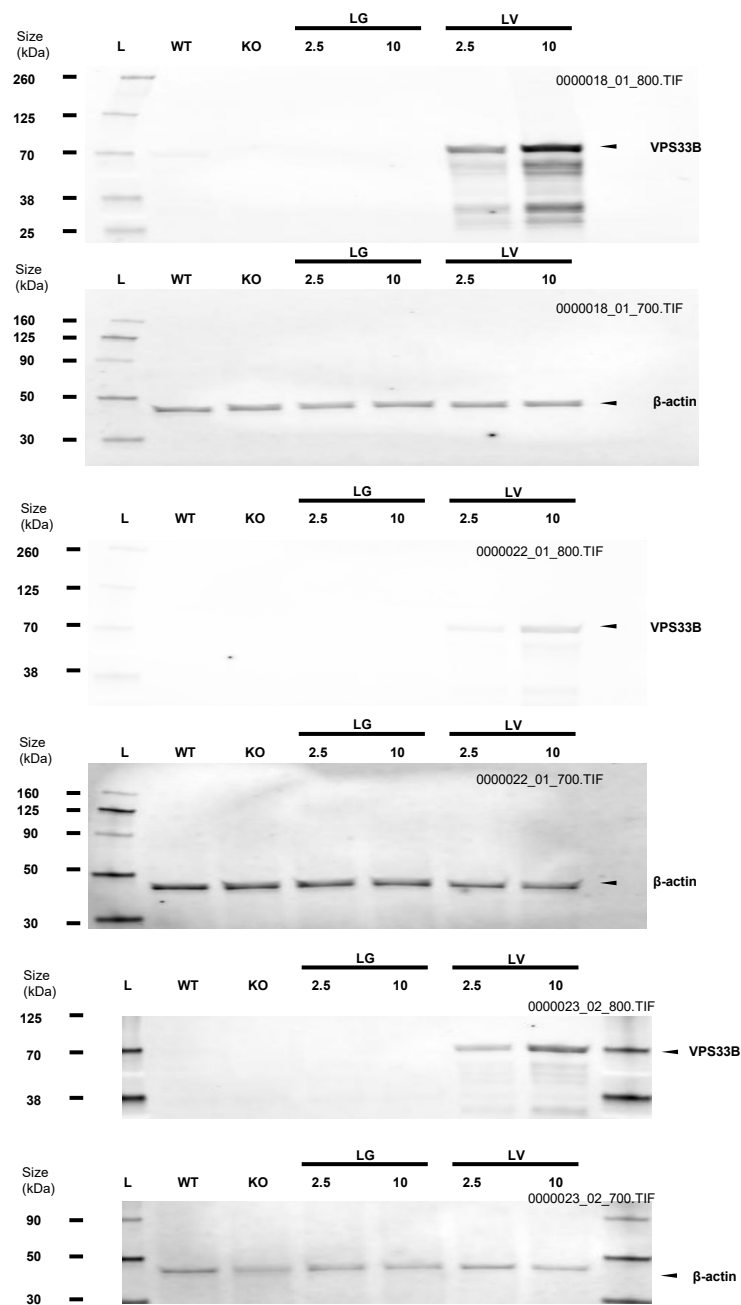

Figure 2E

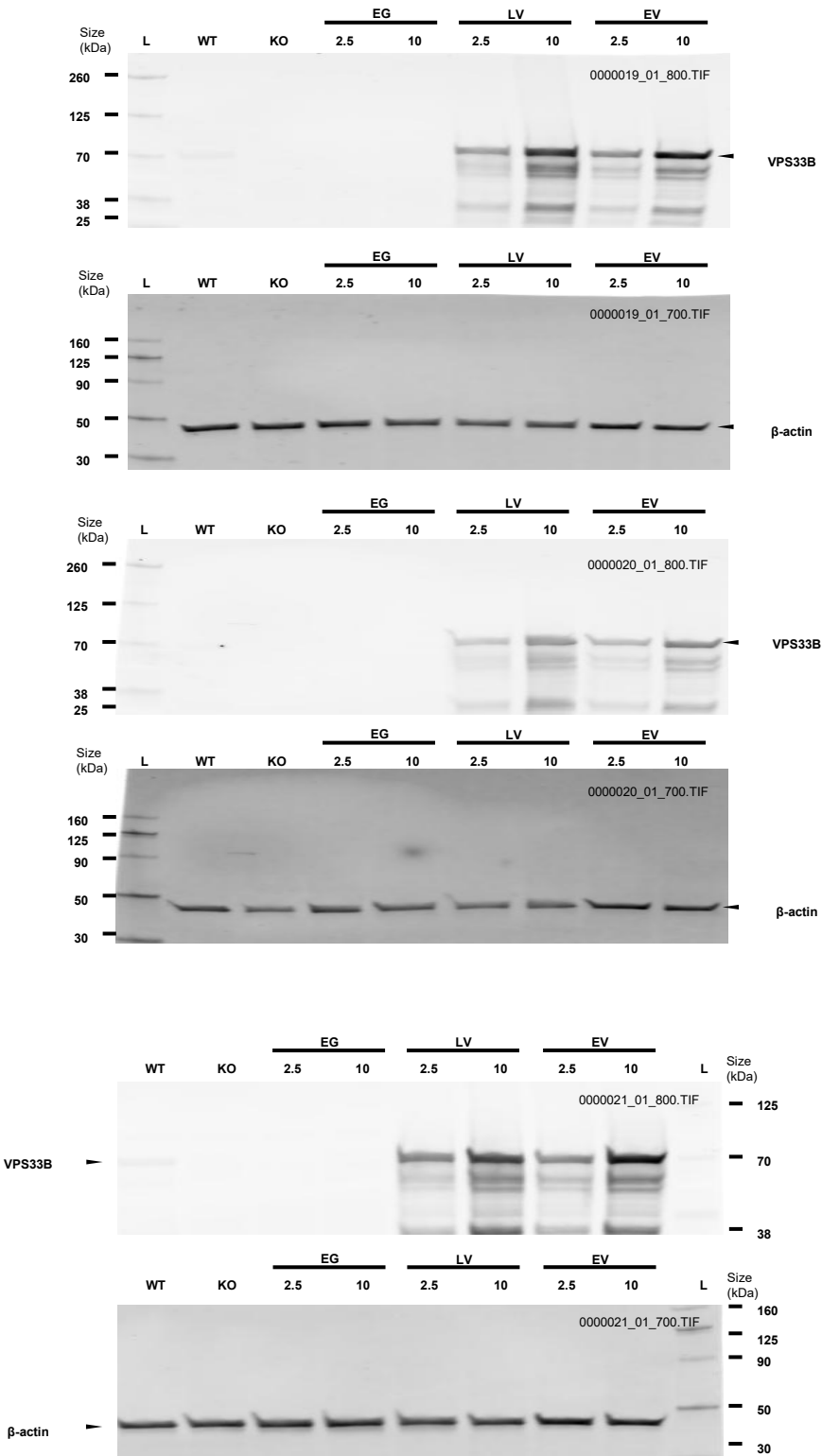

Supplement: Supplementary file 4 — Source data [file 41467_2026_73631_MOESM4_ESM.zip › Uncropped and unprocessed western blots.pdf]
