# Supplementary material for: Safety and efficacy analysis of in vivo lentiviral gene therapy in pre-clinical ARC syndrome models
Source: Nat Commun. 2026 Jun 19;17:5074. doi: 10.1038/s41467-026-73631-x (PMC13282399; doi:10.1038/s41467-026-73631-x)
Supplement: Supplementary file 3 — Transparent Peer Review file [file 41467_2026_73631_MOESM3_ESM.pdf]

nature portfolio

Peer Review File

Safety and efficacy analysis of *in vivo* lentiviral gene therapy in pre-clinical ARC syndrome models

Corresponding Author: Dr Claudiu Cozmescu

This file contains all reviewer reports in order by version, followed by all author rebuttals in order by version.

Version 0:

Reviewer comments:

Reviewer #1

(Remarks to the Author)
The authors show a clear improvement of the ARC syndrome liver phenotype and survival in Vps33b Liver-/- mice upon treatment with a lentivirus expressing the VPS33B transgene under the LP1 promoter. Topic would be of interest for the community. However, in vitro data are weak and mechanisms responsible for carcinogenicity observed in the Vps33bLiver+/- animals treated with the EF1A construct are unclear.
Point-to-point comments are provided below:

Line 71-72, paragraph 81-103, figure S1, figure 1, figure 2
- I disagree with the statement that "both vectors improved the disease-relevant phenotype in vitro" as phenotype has not been clearly characterized in the KO HepG2 cells and the rescue is not obvious.
- Figure S1c) HepG2 does not seem to be the most appropriate cell type for a VPS33B knock out as VPS33B expression seems to be very low (as shown in fig S1c) or even absent (see fig 1F and 2F). Also, mRNA levels should be undetectable in knock-out clones (S1b). Was RNA extracted from a pure clonal population? Could it be that the detected signal is coming from contaminating DNA? Was the sample DNAse treated? Were the primers designed in different exons? Showing the raw Ct value would also help with the interpretation of the data.
- Figure S1d) authors should highlight the region from S1C that was used for normalization
- Figure S1e) a representative image for each treatment is not sufficient. A quantification of the canaliculi/phenotype from several images should be provided.

- Authors should use a different hepatic cell line with higher basal VPS22B levels and more obvious canaliculi structure. I wonder if HepaRG or primary Human Hepatocyte may be appropriate. Data as presented in HepG2 are not convincing.

- Figure 1b) authors should explain why wtVPS33B levels are shown. Was it to prove that endogenous levels are not affected by viral infection? Or just for future comparison with fig 1D?
- Figure 1c) VCN is an acronym. It should be written in capital and the unit of measurement (e.g. viral genomes/diploid genome, viral copies/cell, etc) should be added every time a VCN value is reported. This correction should be applied to all the figures and to the text.
- Figure 1c) It is puzzling that cells infected with an MOI of 2.5 have a VCN of ~5 vg/dg and cells infected with 10 have a VCN higher than 25 vg/dg. It makes me wonder if the indicated MOI doses are correct or the VCN quantification is overestimated.
- Figure 1f) the use of bands from different gels is unacceptable. If relevant bands from the same gel have been shown and the others were excluded as not relevant to the result, the original image showing the whole panel should be shown somewhere. Otherwise, a new gel only containing the relevant treatment should be run.
- Figure 1i-k) in all graphs, the effects of the control virus are not shown. Effects of control viruses on CEA and MRP2 canaliculi should be shown to prove that the positive effects are linked to transgene expression and not by the viral transduction per se. Also, higher VCN do not seem to increase the number of CEA and MRP2 canaliculi. Is it because a dose of 2.5 has already reached a plateau? It does not seem so as the wt has higher values.
- For all pictures: What is the unit for MOI?
- Figure 2b) The authors evaluate if the difference between LP1 and EF1a in cell viability is statistically significant. Based on the graph data, I wonder if viability of KO cells is higher than wt and, if so, what could be the mechanism responsible for this observation.

- Figure 2d) please change vcn to “VCN” and add the unit to the graph
- Figure 2h) Images from KO cells should be added as well as quantification of CEA and MRP2 quantifications (as in fig. 1i-k) for all the conditions. As only one image for condition is shown and the KO is not shown, I struggle to agree with the statement in line 102-103 “both treatments restored CEA and MRP2 co-expression in HepG2 VPS33B-/- cells”

Integration site analysis, line 136-137, table S1, line 154-156, carcinogenicity
- Interpretation of the integration site analysis may be misleading. It is true that integration in Tox has been found in all the cancer samples (line 136-137, line 242-243) but, based on the fact that Tox was not amongst the 5 most frequent sites in the cancer samples (Table S1) and the least frequent sample was between 1.6 and 7.3%, it is unlikely that integration in Tox has played a common role in tumorigenicity. Also, Tox is not a cancer associated gene according to all databases consulted (e.g. COSMIC, Intogen, etc). For this reason, I do not support the statement “the above data supports the safety of the LP1-VPS vector but highlights signs of insertional mutagenesis for the EF1-VPS vector”. I would suggest the use of insertional oncogenesis, as insertional mutagenesis (= mutations due to integrations) is intrinsic to the MoA of any integrating virus.
- If viral integration was responsible for insertional oncogenesis, different parts of the same tumour should present the same integration profile (as only observed for C1), integration profile should suggest mono or oligoclonality (as for C1) and the dominant integration(s) should occur in a certified cancer associated gene (as observed in none of the tumours, as Hs2st1 is not a cancer associated gene). A clear statement that insertional oncogenesis does not seem to be the driver of tumour transformation should be added.
- Other mechanisms related to viral transduction may be responsible and need to be added. If possible and material is available, authors should investigate genetic profile of the tumour samples to identify mutations that may have been responsible for tumour transformation.
- Integration profile from liver of animals transduced with the safe LP1 construct should have been added to show similarity/differences between the two viruses.

Figure 4
4b) please change vcn to “VCN” and add the unit to the graph

Figure 7
7d-f) graphs representing a quantification of the data shown should be provided to strengthen the results.

Line 217
Statement “we evaluated the in vitro and in vivo safety and efficacy of two lentiviral vector” is incorrect as safety was not evaluated for any of them.

Line 260-265
Although a few relevant papers have been cited, VSP33B is not classified as cancer-associated gene, hence the weight of evidence that VSP33B may be an oncosuppressor is missing. The statement that “the Vps33bLiver+/- mouse model, which has a single functional Vps33b allele in hepatocytes, may be prone to developing hepatocellular carcinoma” is only speculative.
Line 279
Is treatment with clodronate liposome a therapeutic option for patients?
Line 283-286
Could the authors elaborate more on the role of cholic acid if hypothetically treating patients? Would it be required? Or is it just a way to exacerbate the murine phenotype to make it more similar to the human one?

Line 292-297
In principle, this reviewer agrees with the statements, even though in their opinion this is not a case of insertional oncogenesis and other mechanisms may have been responsible. However, in agreement with the statements in line 292-297, the reviewer would suggest considering integration site analysis in relevant human cell lines (e.g. primary hepatocyte) transduced in vitro to predict IS profile in patients.

(Remarks on code availability)

Reviewer #2

(Remarks to the Author)
In this manuscript, Cozmescu et al. have developed two recombinant lentivirus vectors. These vectors vary in their design: one utilizes a codon-optimized cDNA of VPS33B under the control of a liver-specific promoter (LP1 named LV), and the other uses a ubiquitous promoter (EF1a named EV), both intended for the treatment of ARC syndrome caused by VPS33B deficiency. They analyzed the safety of lentiviral vector administration in heterozygous animals and found that the administration of the lentiviral vector carrying the EF1a promoter, independently of the transgene (VPS33B or GFP), induced the formation of liver malignancies, while the Lentiviral vector with the LP1 promoter did not. They also found that the Lentivirus EF1a was associated with integration in genes potentially linked to liver tumor formation. Finally, they evaluated the therapeutic efficacy of the safer LV vector administered in combination with clodronate-loaded liposomes in a model of hepatic ARC showing a beneficial effect.
These findings are very relevant and raise concerns about the safety of systemic LV administration. Although the mice receiving the vector with the LP1 promoter showed no malignancies, the authors should indicate that the vector copy number

in this group was lower.

Furthermore, there are a number of important issues and inconsistencies that need to be properly addressed by the authors.

Major Comments

Efficacy evaluation was performed in Vps33b Liver-/- animals pretreated with clodronate-loaded liposomes and receiving the vector at P4, with sacrifice 12 weeks later. In contrast, safety testing was performed in Vps33b Liver+/- animals receiving the vector at P2 in the absence of clodronate pretreatment, with sacrifice 36 weeks later. These differences might have a significant impact on safety and are not mentioned by the authors.

The authors should clearly indicate in the abstract that the treatment consisted of the administration of the viral vector together with macrophage depletion using clodronate. They should also discuss whether this protocol can be transferred to the clinic.

Figure 6 presents very important issues.
To evaluate the therapeutic efficacy of the vector one month after treatment, LV animals showed an 80% survival rate, compared to 33.3% for LG-treated animals. If the experiment was performed for 12 weeks, why are the authors not representing the follow-up data to the end of the experiment?
More importantly, if the study started with 6 animals per group, by week 8 of age (4 weeks after CA diet), only 2 animals of the LG group would remain alive. However, in the ALP value at 12 weeks of age, data from 6 animals are represented, which is not possible. The same applies to the values in the rest of the graphs, which, according to the legend, were obtained at harvest in n = 6 in all the groups, in the LG group should be 2 and in the LV group 5. A potential explanation to this incongruency is that the values were obtained at sacrifice but this should be clearly explained.
Supplementary Figures 2 and 3 deserve a better description, explanation, and discussion of the data. Why does the "therapeutic" efficacy of LV seem to be better in the absence of clodronate than when clodronate was administered and transduction improved? Ha really LV provide a selective advantage) is really therapeutic?
In fact, the authors claim that the increase in VCN levels was due to a selective advantage of transduced hepatocytes. However, if this is the case, a significant difference should be observed between the LG group (which does not have a selective advantage) and the LV group. In fact, GFP expression levels are higher than VPS33B.

Additional Comments:
Introduction
"Lentiviral vectors (LV) have been used in ex vivo and in vivo clinical trials and received regulatory approvals for the treatment of monogenic disorders6–8" No clinical data are reported in those references for in vivo clinical trials; please modify this statement.
Results
VPS33B protein analysis in the liver by immunohistochemistry should be included, and the levels of fibrosis and bile duct numbers should be quantified, plotted, and statistically analyzed.
Discussion
The authors should highlight the limitation of the model, since liver pathology has to be induced by a cholic acid diet.

(Remarks on code availability)

Version 1:

Reviewer comments:

Reviewer #1

(Remarks to the Author)
I commend the authors for addressing most of the concerns through the provision of additional data that now better support the in vitro statements. Regarding insertional oncogenesis, although I do not entirely agree with their interpretation, the data are adequate to enable readers to form their own opinion, and it is beneficial to have these findings reported.

(Remarks on code availability)

Reviewer #2

(Remarks to the Author)
The authors have made a significant effort to address the reviewers' questions, which is greatly appreciated. However, two points still require attention.
First, the clinical use of agents to deplete macrophages in the liver typically requires systemic administration, whereas the examples provided by the authors are based on local injection. The authors should therefore be cautious when discussing the translatability of this approach.
Second, the reference used to support the statement "Lentiviral vectors (LV) have been used in ex vivo and in vivo clinical

trials and received regulatory approvals for the treatment of monogenic disorders" (Palfi et al., Long-Term Follow-Up of a Phase I/II Study of ProSavin, a Lentiviral Vector Gene Therapy for Parkinson's Disease, Human Gene Therapy Clinical Development, 2018) is not appropriate. Parkinson's disease is not a monogenic disorder, and the vector administration in this study was not systemic. In fact, one of the limitations of lentiviral vectors for systemic delivery is the lack of clinical experience, and the authors should clearly acknowledge this point.

(Remarks on code availability)
Yes they provide enough information to run the application

Open Access This Peer Review File is licensed under a Creative Commons Attribution 4.0 International License, which permits use, sharing, adaptation, distribution and reproduction in any medium or format, as long as you give appropriate credit to the original author(s) and the source, provide a link to the Creative Commons license, and indicate if changes were made.
In cases where reviewers are anonymous, credit should be given to 'Anonymous Referee' and the source.
The images or other third party material in this Peer Review File are included in the article's Creative Commons license, unless indicated otherwise in a credit line to the material. If material is not included in the article's Creative Commons license and your intended use is not permitted by statutory regulation or exceeds the permitted use, you will need to obtain permission directly from the copyright holder.
To view a copy of this license, visit https://creativecommons.org/licenses/by/4.0/

Reviewer #1 (Remarks to the Author):

The authors show a clear improvement of the ARC syndrome liver phenotype and survival in Vps33b Liver-/- mice upon treatment with a lentivirus expressing the VPS33B transgene under the LP1 promoter. Topic would be of interest for the community. However, in vitro data are weak and mechanisms responsible for carcinogenicity observed in the Vps33b Liver+/- animals treated with the EF1A construct are unclear.

Author comments:

We thank the reviewer for recognizing the therapeutic impact and relevance of our findings. We agree that clarification of the in vitro data and expanded discussion on mechanisms contributing to the carcinogenicity observed with the EF1α-driven construct is important. To address these points, we have performed further experiments and expanded our analyses. Each concern is addressed in detail below.

Point-to-point comments are provided below:

1. HEPG2 cells Line 71-72, paragraph 81-103, figure S1, figure 1, figure 2
- I disagree with the statement that “both vectors improved the disease-relevant phenotype in vitro” as phenotype has not been clearly characterized in the KO HepG2 cells and the rescue is not obvious.

Author comments:

We thank the reviewer for their constructive comments regarding data presentation. In response, we have performed additional experiments to further characterise the KO HepG2 model and to strengthen the robustness of the in vitro data. These include improved western blot analyses with higher-quality images, repetition of all experiments in biological triplicates, and quantitative assessment of both the number and surface area of CEA-positive bile canaliculi (Fig. S1). In addition, we now provide qualitative evidence of mislocalisation of multiple apical proteins in the HepG2 VPS33B-/- clone 4 compared with wild-type cells (Fig.S2-3). Together, these new data reinforce the relevance of the KO HepG2 cells as a robust model of the ARC syndrome liver phenotype and substantially improve both the scientific rigor and clarity of data presentation to meet the standards of the journal.

- Figure S1c) HepG2 does not seem to be the most appropriate cell type for a VPS33B knock out as VPS33B expression seems to be very low (as shown in fig S1c) or even absent (see fig 1F and 2F).

Author comments:

We thank the reviewer for raising this important point regarding VPS33B expression levels in HepG2 cells. Although VPS33B plays a critical role in maintaining hepatocyte polarity and bile canalicular

organisation, its expression in the liver is known to be relatively low compared with other tissues. Consistent with this, RNA expression data from the Human Protein Atlas indicate that the liver is among the tissues with the lowest VPS33B transcript levels (Supporting Figure 1). Importantly, low basal expression does not prevent functional relevance, as loss of VPS33B results in profound defects in apical trafficking and hepatocyte polarity, which underlie the cholestatic liver phenotype observed in ARC patients. Thus, despite modest endogenous expression, HepG2 cells provide a relevant system to analyse VPS33B-dependent hepatocellular polarity and trafficking defects.

In addition, the antibody used in the initial western blot analyses was the only commercially available reagent that consistently detected VPS33B, albeit alongside several non-specific bands. The combination of low endogenous VPS33B expression and limited antibody specificity contributed to the weak VPS33B signal observed in wild-type HepG2 cells in the original figures. To address this, we have repeated all experiments in biological triplicates and validated a new batch of the antibody that provides more reliable detection of VPS33B. Accordingly, we have replaced the original western blot panels with new representative blots in Figure 1D (replacing Figure S1C), Figure 1E (replacing Figure 1F), and Figure 2D (replacing Figure 2F). These revised figures clearly demonstrate the presence of the VPS33B-specific band in wild-type cells, its absence in knockout and mock-treated cells, and its restoration or overexpression following lentiviral treatment.

A

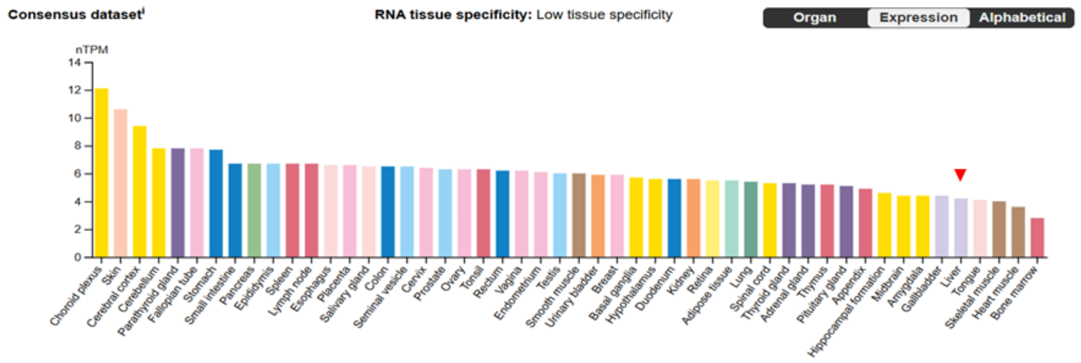

B

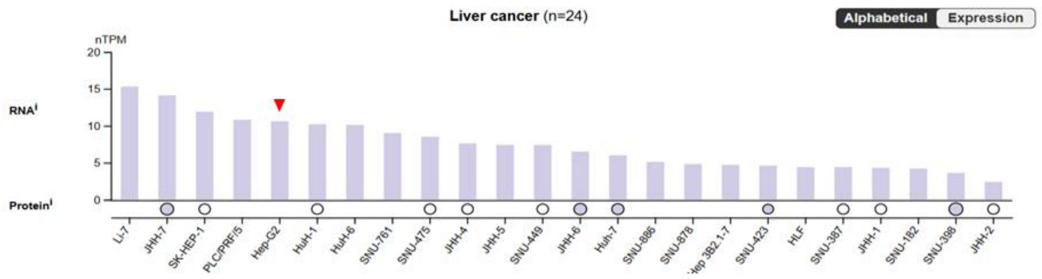

Supporting Figure 1: RNA expression of VPS33B. (A) *Expression in human tissues.* ***(B)*** *Expression in hepatic cell lines. Red arrow points toward the areas of interest. Data was sourced from https://www.proteinatlas.org/.*

- Also, mRNA levels should be undetectable in knock-out clones (S1b).

Was RNA extracted from a pure clonal population? Could it be that the detected signal is coming from contaminating DNA? Was the sample DNAse treated? Were the primers designed in different exons? Showing the raw Ct value would also help with the interpretation of the data.

Author comments:

We are grateful to the reviewer for this insightful comment, which prompted us to clarify the interpretation of VPS33B mRNA detection in the knockout samples. We agree that the presence of residual transcript deserves careful consideration (now Fig. S1C). However, low-level mRNA detection in CRISPR-Cas9–edited cells is a well-recognised phenomenon and does not necessarily indicate residual protein expression (as confirmed now by all the new Western Blots provided). As shown in the sequencing data now provided (Fig. S1B), we confirmed precise editing at the CRISPR-Cas9 target site in the knockout clones used in this study. Sanger sequencing of Clone 4, which was selected for all subsequent experiments, demonstrates a homogeneous edited population carrying a single adenine insertion at the Cas9-induced double-strand break site. This insertion introduces a frameshift and a premature stop codon in the VPS33B transcript (Supporting Figure 2), rendering it non-functional and targeting it for nonsense-mediated decay (NMD). As previously reported[1], NMD does not always result in complete transcript elimination, and low levels of residual mRNA may therefore remain detectable[1]. Importantly, due to the early stop codon, any remaining transcript would not be expected to give rise to functional VPS33B protein. Consistent with this interpretation, overexposed western blot analysis reveals a clear VPS33B-specific band in wild-type cells that is absent in knockout cells (Supporting Figure 3). We would be happy to include this figure in the supplementary material if deemed necessary by the reviewers.

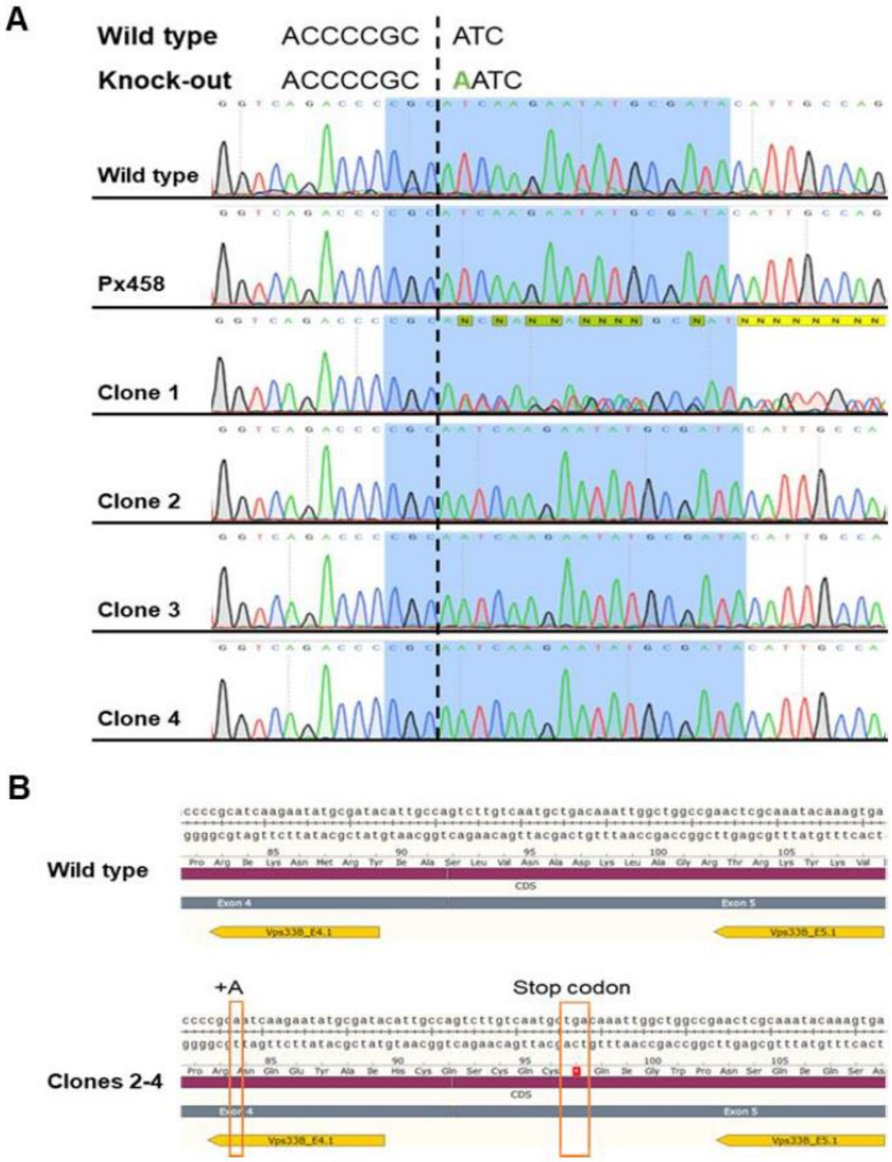

Supporting Figure 2: Sanger sequencing of the cutting sites associated to the gRNA used in the CRISPR-Cas9 knock-out. (A) *Amplicons covering the gRNA target region were PCR-amplified from genomic DNA of wild-type HepG2 cells and possible VPS33B knock-out (KO) clones. Sanger sequencing showed that the Px458-treated line had a trace identical to wild-type, indicating no editing. In contrast, clones 1–4 displayed sequence modifications. Clone 1 showed a mixed trace suggestive of a heterozygous edit, while clones 2–4 had a clear adenine insertion 5' of the Cas9 cut site guided by the used gRNA (marked with a dotted line).* ***(B)*** *The SnapGene map illustrates that this insertion creates a premature TGA stop codon upstream of the gRNA site, consistent with a functional VPS33B knockout in clones 2–4.*

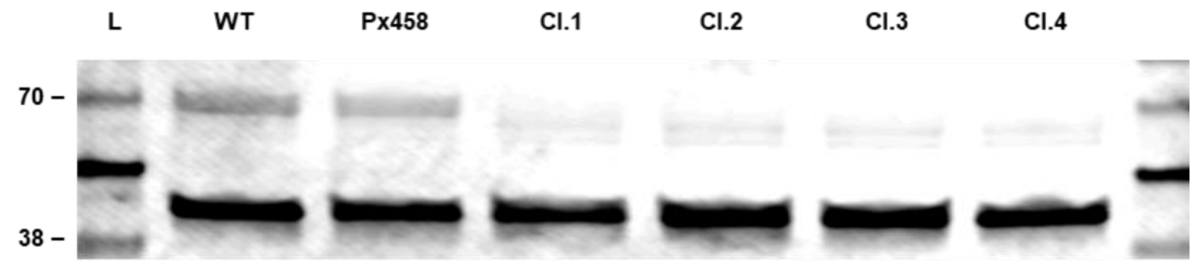

Supporting figure 3. Overexposed Western blot. *The Western blot from Fig. S1D overexposed and converted to grayscale shows no VPS33b specific bands at 70 kDA in the knock-out clones.*

The mRNA samples used for qPCR were not treated with DNAse to further remove possible genomic DNA contamination, however one of the primers for amplification has been placed at the junction between 2 exons to ensure the full primer sequence is not found in the genomic DNA (Supporting Figure 4). We also have RNA-seq data (not included in the paper) comparing WT vs Clone 4 RNA expression which show similar levels of VPS33B mRNA as the qPCR. (Supporting Table 1)

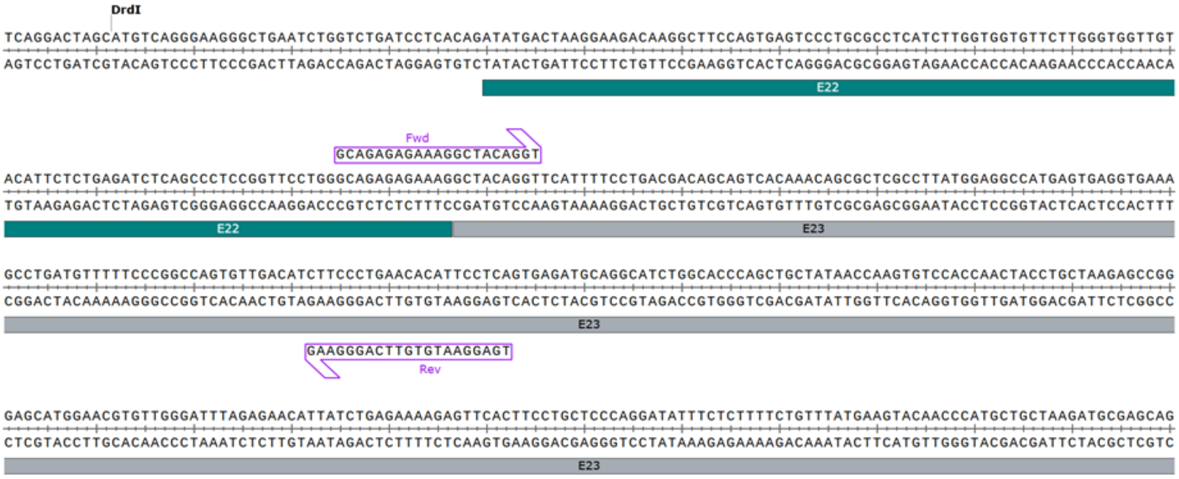

Supporting Figure 4: Annealing location of the primers used for the qPCR analysis of the wild type VPS33B expression. *The Fwd primer has been placed at the junction between exon 22 and 23 to ensure the full primer sequence is not present in the genomic DNA.*

Supporting Table 1: RNA-Seq data comparing Clone 4 and WT levels of VPS33B expression.

ID	log2FoldChange	pvalue	padj	Cl4	WT	Gene.name
ENSG00000184056	-1.7455	0.2626	0.9999	231.0213	774.6601	VPS33B

We believe this is sufficient to justify the presence of RNA

- Figure S1d) authors should highlight the region from S1C that was used for normalization

Author comments:

We thank the reviewer for this helpful suggestion, which has improved the clarity of the data presentation. In the revised manuscript, we have replaced the original panels with new, higher-quality western blots that clearly and unambiguously show the VPS33B-specific band <<Figure 1D (replacing Figure S1C), Figure 1E (replacing Figure 1F), and Figure 2D (replacing Figure 2F)>>. For normalisation, the VPS33B signal was quantified relative to the corresponding band detected in wild-type cells, which served as the reference. This is now clearly indicated in the updated figure and accompanying legend. Importantly, quantification was performed using n=3 *independent western blots, generated from cells harvested in different weeks, to robustly assess the reduction in VPS33B protein levels in the isolated knockout clones compared with wild-type cells. This information is now clearly indicated in the updated figure and legend (Fig. S1E).*

- Figure S1e) a representative image for each treatment is not sufficient. A quantification of the canaliculi/phenotype from several images should be provided.

Author comments:

We appreciate the reviewer's suggestion to strengthen the analysis by including quantitative data. Accordingly, the original Fig. S1E has been replaced with Fig. S1F in the revised manuscript. Given that CEA is mislocalised from bile canaliculi in both ARC patients and murine models with VPS33B deficiency[2], we quantified the number of CEA-positive bile canaliculi and the total canalicular area as markers of the disease phenotype. Images were acquired at 20× magnification, and analysis was performed across three independent fields of view per group, ensuring a representative and rigorous assessment. The results have shown consistent reduction in CEA^{positive} bile canaliculi number and area in the isolated clones compared to WT cells (Fig. S1G-H), supporting the HepG2 VPS33B$^{-/-}$ cells as a good in vitro model for the ARC syndrome hepatocyte phenotype.

- Authors should use a different hepatic cell line with higher basal VPS22B levels and more obvious canaliculi structure. I wonder if HepaRG or primary Human Hepatocyte may be appropriate. Data as presented in HepG2 are not convincing.

Author comments:

We appreciate the reviewer's thoughtful recommendation to consider alternative hepatic models, such as HepaRG cells or primary human hepatocytes. We carefully evaluated these options; however, we believe that HepG2 cells represent an appropriate and well-justified model for studying the hepatocellular phenotype associated with ARC syndrome for several reasons outlined below.

First, although VPS33B plays a critical functional role in hepatocyte polarity, the liver is among the tissues with the lowest basal VPS33B expression (Supporting Figure 5A). Consistent with this physiological context, HepG2 cells (Supporting Figure 5B) nevertheless rank among the top hepatic cell lines in terms of VPS33B RNA expression, making them suitable for loss-of-function studies (https://www.proteinatlas.org/).

Second, the ability of HepG2 cells to polarise and form bile canaliculi-like structures is well established and critically important for modelling VPS33B-associated defects in apical trafficking. A PubMed search using the terms "HepG2," "polarity," and "apical" identifies over 40 publications spanning 1993 to 2025, underscoring the extensive validation of this model. In the present study, we adopted and optimised protocols from one of these reports to enhance bile canaliculi formation[3]. Given that VPS33B mutations disrupt intracellular trafficking of apical proteins, including CEA[2], the well-characterised polarisation capacity of HepG2 cells is essential for accurately modelling key aspects of the ARC syndrome liver phenotype in vitro.

Third, we have now included in the revised manuscript additional data in Figure S1 demonstrating that loss of VPS33B protein in HepG2 cells is directly associated with a reduction in CEA-positive bile canaliculi, providing functional validation of the model.

Finally, while primary human hepatocytes can express VPS33B and polarise under specific conditions, their limited lifespan in culture, high cost, and lack of suitability for CRISPR-Cas9–mediated gene knockout make them impractical for the mechanistic and longitudinal studies performed here.

Based on these considerations and the new data provided, we remain confident that HepG2 cells are a robust and appropriate model for investigating the hepatocyte polarity defects underlying ARC syndrome. Nevertheless, we acknowledge the reviewer's comment regarding image quality and have therefore performed additional experiments and provided improved, higher-quality images in the revised manuscript.

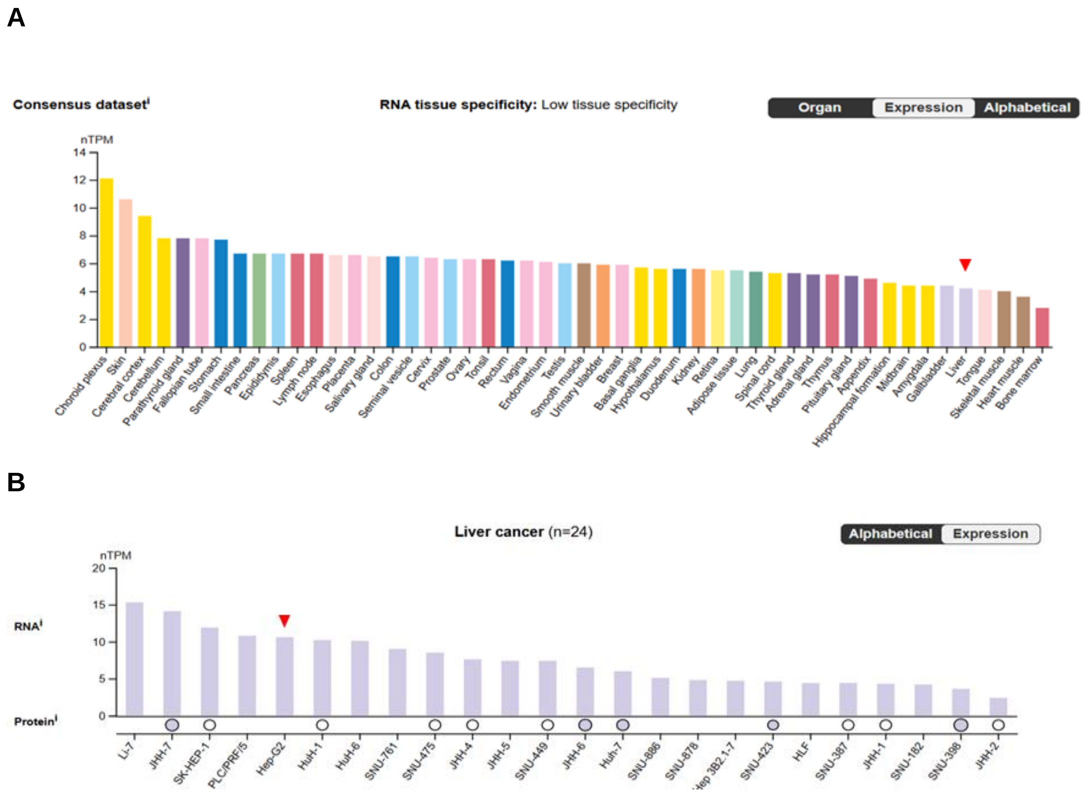

Supporting Figure 5: RNA expression of VPS33B. (A) *Expression in human tissues.* ***(B)*** *Expression in hepatic cell lines. Red arrow points toward the areas of interest. Data was sourced from https://www.proteinatlas.org/.*

- Figure 1b) authors should explain why wtVPS33B levels are shown. Was it to prove that endogenous levels are not affected by viral infection? Or just for future comparison with fig 1D?

Author comments:

We thank the reviewer for this thoughtful question, which helped us reassess the clarity and necessity of this panel. In the original version of the manuscript, Fig. 1B was included to demonstrate that the residual, non-functional VPS33B mRNA detected in the knockout HepG2 cells remained stable across experimental conditions and was therefore unlikely to act as a confounding factor. At that stage, western blot images were of lower quality, and this RNA-level control provided additional reassurance that observed phenotypes were not driven by variability in transcript abundance.

In the revised manuscript, we now present higher-quality western blot data that clearly demonstrate the absence of VPS33B protein in knockout cells, rendering residual mRNA levels functionally irrelevant

(Fig. 1E-F). To avoid unnecessary complexity and potential confusion, we have therefore removed Figure 1B from the revised version of the manuscript.

- Figure 1c) VCN is an acronym. It should be written in capital and the unit of measurement (e.g. viral genomes/diploid genome, viral copies/cell, etc) should be added every time a VCN value is reported. This correction should be applied to all the figures and to the text.

Author comments:

We agree with the reviewer and thank them for highlighting this point. The acronym VCN is now consistently capitalised, and the unit of measurement has been clearly specified each time VCN values are reported. These corrections have been applied throughout the manuscript, including the main text, all relevant figures, and figure legends. In the revised manuscript this point is relevant now to Fig. 1B and D.

- Figure 1c) It is puzzling that cells infected with an MOI of 2.5 have a VCN of ~5 vg/dg and cells infected with 10 have a VCN higher than 25 vg/dg. It makes me wonder if the indicated MOI doses are correct or the VCN quantification is overestimated.

Author comments:

We thank the reviewer for raising this important point. We initially shared the reviewer's concern and carefully considered both possibilities, namely inaccuracies in the indicated MOI and potential overestimation of VCN. Upon further investigation, we identified a more likely explanation related to differences in transduction efficiency between cell types used for viral titration.

As is common practice, lentiviral vectors were initially titrated in HEK293T cells. However, we have observed that functional vector titers can be 4–10-fold higher in HepG2 cells compared with HEK293T cells. Consequently, when MOI values calculated based on HEK293T titration are applied to HepG2 cells, the resulting VCN can appear disproportionately high. We therefore attribute the observed discrepancy between MOI and VCN to the higher permissiveness of HepG2 cells to lentiviral transduction rather than to errors in MOI assignment or VCN quantification.

To address this in the revised experiments, we have now titrated the vectors directly in HepG2 cells. Under these conditions, post-transduction VCN values show close concordance with the applied MOI, supporting this interpretation and further validating the revised data.

- Figure 1f) the use of bands from different gels is unacceptable. If relevant bands from the same gel have been shown and the others were excluded as not relevant to the result, the original image showing the whole panel should be shown somewhere. Otherwise, a new gel only containing the relevant treatment should be run.

Author comments:

We thank the reviewer for highlighting this important issue and we fully agree that the use of bands derived from different gels is not acceptable. We would like to clarify that the western blot shown in the original Fig. 1F was in fact derived from a single gel, from which two lanes (C5 and V5) were excluded for simplicity and consistency, as these conditions were not relevant to the specific comparison being presented and were omitted throughout the manuscript. We also acknowledge the reviewer's observation that the wild-type band was not clearly visible in the originally selected image. In response, and as described above, we have repeated the experiments and now present new, higher-quality western blot figures in the revised manuscript (Fig. 1E). All updated western blots are shown in full and derived from single gels to avoid any ambiguity or confusion.

We believe these changes fully address the reviewer's concern and improve both the rigor and clarity of the data presentation.

- Figure 1i-k) in all graphs, the effects of the control virus are not shown. Effects of control viruses on CEA and MRP2 canaliculi should be shown to prove that the positive effects are linked to transgene expression and not by the viral transduction per se. Also, higher VCN do not seem to increase the number of CEA and MRP2 canaliculi. Is it because a dose of 2.5 has already reached a plateau? It does not seem so as the wt has higher values.

Author comments:

We thank the reviewer for this important comment and agree that demonstrating the effect of the control virus is necessary to exclude an impact of viral transduction per se. These experiments were performed. However, in the initial experiments quantification of MRP2^{positive} canaliculi in control vector–treated cells was technically confounded by GFP expression from the control lentiviral vector, which interfered with detection using the Alexa-488-conjugated secondary antibody.

For the revised manuscript we have used a different secondary antibody for detection of MRP2 (Alexa-647-conjugated). To be consistent with the initial clone characterisation, we have focused quantitative analyses on CEA^{positive} canaliculi, while MRP2 localisation is presented qualitatively to demonstrate its correct targeting to bile canaliculi following VPS33B expression. As shown in the revised data, the number of CEA^{positive} canaliculi in control vector–treated knockout cells was comparable to untreated

knockout cells across all MOIs, demonstrating that the observed rescue is linked to VPS33B transgene expression rather than viral transduction (Fig. 1G-H).

- For all pictures: What is the unit for MOI?

Author comments:

We thank the reviewer for this question and are happy to clarify. The multiplicity of infection (MOI) is a dimensionless parameter and does not have a unit of measurement. It refers to the ratio of viral particles (or functional viral units, depending on titration method) administered per target cell. To avoid ambiguity, we now explicitly clarify this definition in the figure legends and Methods section where MOI values are reported.

- Figure 2b) The authors evaluate if the difference between LP1 and EF1a in cell viability is statistically significant. Based on the graph data, I wonder if viability of KO cells is higher than wt and, if so, what could be the mechanism responsible for this observation.

Author comments:

We thank the reviewer for this insightful observation. As shown in the original Fig. 2B, cell viability in VPS33B knockout cells appeared modestly higher than in wild-type cells. While the precise mechanism underlying this difference remains unclear, one possible explanation is that loss of VPS33B leads to disruption of junctional complexes that normally seal bile canaliculi, potentially facilitating increased cell proliferation. Consistent with this hypothesis, a similar proliferative advantage has been reported in mouse inner medullary collecting duct (mIMCD-3) cells with mutations in VIPAR[4], the functional partner of VPS33B, as well as in keratinocytes with dysfunctional VPS33B or VIPAR[5]. These observations support emerging evidence that the VPS33B-VIPAR complex may exert tumour-suppressive functions[6,7].

As cell viability was not reassessed in the newly performed experiments, we have moved this panel to the Supplementary Information to avoid overinterpretation and to maintain consistency with the revised dataset.

- Figure 2d) please change vcn to “VCN” and add the unit to the graph

Author comments:
We thank the reviewer for this helpful comment. The acronym has now been corrected to VCN, and the appropriate unit of measurement has been added to the graph in Fig. 2B (replacing original Fig. 2D).

- Figure 2h) Images from KO cells should be added as well as quantification of CEA and MRP2 quantifications (as in fig. 1i-k) for all the conditions. As only one image for condition is shown and the KO is not shown, I struggle to agree with the statement in line 102-103 “both treatments restored CEA and MRP2 co-expression in HepG2 VPS33B-/- cells”

Author comments:

We thank the reviewer for this helpful comment and agree that inclusion of knockout images and quantitative analyses is important to support this conclusion. In the revised manuscript, original Fig. 2H has been replaced with Fig. 2F-G. The new figures include representative images from knockout cells treated with the EF1-GFP control vector at MOIs of 2.5 and 10 to control for the effects of viral transduction. For consistency with earlier analyses, we have quantified CEA-positive bile canaliculi across conditions, while MRP2 localisation is shown qualitatively to demonstrate its correct targeting to the bile canaliculi. These revisions strengthen the evidence supporting restoration of canaliculi markers following VPS33B expression and address the reviewer’s concern regarding the statement in line 102-103 “both treatments restored CEA and MRP2 co-expression in HepG2 VPS33B-/- cells” (Now line 109-110).

Integration site analysis, line 136-137, table S1, line 154-156, carcinogenicity
- Interpretation of the integration site analysis may be misleading. It is true that integration in Tox has been found in all the cancer samples (line 136-137, line 242-243) but, based on the fact that Tox was not amongst the 5 most frequent sites in the cancer samples (Table S1) and the least frequent sample was between 1.6 and 7.3%, it is unlikely that integration in Tox has played a common role in tumorigenicity. Also, Tox is not a cancer associated gene according to all databases consulted (e.g. COSMIC, Intogen, etc). For this reason, I do not support the statement “the above data supports the safety of the LP1-VPS vector but highlights signs of insertional mutagenesis for the EF1-VPS vector”. I would suggest the use of insertional oncogenesis, as insertional mutagenesis (= mutations due to integrations) is intrinsic to the MoA of any integrating virus.

Author comments:

We agree with the reviewer that Tox is unlikely to represent a driver mutation in the observed tumours. Our intention was not to imply causality, but rather to report that Tox was the only gene meeting our predefined criteria (integration frequency >0.1%) that was both dysregulated and shared across the three tumours, which remains a factual observation. As stated in the manuscript, we performed a comprehensive analysis of all integration sites exceeding the 0.1% threshold, and Tox emerged as a common hit under these specific parameters.

To avoid any potential misinterpretation, we have now added explicit clarification in the revised manuscript stating that “downregulation of Tox has not previously been reported in HCC cells, and we

do not consider it to be a likely driver mutation in this context." This statement has been inserted in lines 278-279 (highlighted in yellow in the revised manuscript) to clearly delineate observation from causality. For transparency regarding the most frequent integrations, we have also included in the main text the following "Dominant integration sites and their frequencies varied across cancers: Hs2st1 in C1 (>25% across all regions), Cep78 in C2 (2.1%), Arfgap2 in C3.1 (17.3%), and Cdh18 in C3.2 (8.46%)." (lines 145-147 – highlighted in yellow).

In light of the reviewer's comments, we have also revised our conclusions regarding vector safety. The original statement, "The above data supports the safety of the LP1-VPS vector but highlights signs of insertional mutagenesis for the EF1-VPS vector," has been replaced with a more accurate and balanced interpretation: "Although the tumorigenic mechanism is not fully elucidated, EF1-VPS treatment showed signs of insertional oncogenesis. In contrast, the absence of tumours in LP1-VPS-treated animals, with or without clodronate, together with a Shannon equitability index close to 1, supports the safety of this vector for in vivo use." (now line 172-175 - highlighted in yellow)

In addition, we have replaced the term "insertional mutagenesis" with "insertional oncogenesis" throughout the manuscript to better reflect the biological interpretation of the data. We thank the reviewer for this constructive feedback, which has helped us refine both the interpretation and precision of our conclusions.

- If viral integration was responsible for insertional oncogenesis, different parts of the same tumour should present the same integration profile (as only observed for C1), integration profile should suggest mono or oligoclonality (as for C1) and the dominant integration(s) should occur in a certified cancer associated gene (as observed in none of the tumours, as Hs2st1 is not a cancer associated gene). A clear statement that insertional oncogenesis does not seem to be the driver of tumour transformation should be added.

Author comments:

We thank the reviewer for this comment and welcome the opportunity to clarify the tumour sampling strategy and interpretation of the integration site analysis. As detailed in Supplementary Table S1, the cancerous tissues analysed were collected from distinct anatomical regions of the liver. While tumour C1 is likely to originate from a single mutational event, tumours C3.1 and C3.2 were harvested from different liver lobes and are therefore likely to result from independent oncogenic events. Accordingly, their lentiviral integration patterns would not be expected to be identical. Importantly, as shown in Fig. 4D and 4E, all cancer samples subjected to further analysis displayed clear evidence of oligoclonal expansion, characterised by low Shannon diversity scores and dominant clones.

We would also like to clarify that, contrary to the reviewer's statement, dominant lentiviral integrations are not required to occur within a recognised cancer-associated gene to contribute to oncogenic events.

Although self-inactivating lentiviral vectors are generally considered a safe option for gene therapy[8], vectors containing strong internal promoters, such as MNDU3[9], can dysregulate gene expression and activate neighbouring promoters at distances of up to at least 100 kb from the integration sites[10]. Thus, insertional oncogenesis can occur independently of direct integration into a certified cancer-associated gene.

To ensure this interpretation is clearly conveyed, we have added the following clarifying statements to the main text:

"Although self-inactivating lentiviral vectors are generally considered safe, vectors containing strong internal promoters (e.g. MNDU3 (32)) can dysregulate gene expression and activate neighbouring promoters to at least 100 kb from the integration site, thereby enabling insertional oncogenesis independently of the targeted gene." (line 281-284 – highlighted in yellow)

"Although the precise mechanism underlying the genotoxicity observed with EF1α promoter containing vectors remains unclear, insertional oncogenesis, likely driven by promoter mediated dysregulation of nearby gene expression, remains the most plausible explanation. Whilst EF1α has proven safe in ex vivo gene therapy (7), its oncogenic potential may be enhanced in a cancer-predisposed liver, such as in Vps33b$^{Liver+/-}$ mice with reduced Vps33b expression (18)." (line 292-297 – highlighted in yellow)

"Together with our safety data, these findings support a two-hit model of cancer development (38), where reduced VPS33B expression acts as a predisposing event requiring additional secondary alterations, possibly from vector integration combined with the stronger EF1α promoter-driven gene expression changes, to result in tumour formation." (line 308-311 – highlighted in yellow)

We hope these clarifications address the reviewer's concern and improve the clarity and accuracy of the manuscript's interpretation.

- Other mechanisms related to viral transduction may be responsible and need to be added. If possible and material is available, authors should investigate genetic profile of the tumour samples to identify mutations that may have been responsible for tumour transformation.

We thank the reviewer for this thoughtful suggestion and agree that mechanisms other than insertional oncogenesis should be considered. In response, we have expanded the discussion to explicitly address additional oncogenic mechanisms potentially associated with viral transduction. Specifically, we considered whether expression of the VPS33B transgene driven by the ubiquitous EF1α promoter in antigen-presenting cells, following lentiviral uptake by macrophages or dendritic cells, could trigger immune-mediated liver injury analogous to autoimmune hepatitis. However, such a mechanism would

be expected to result in reduced VCNs in EF1α-treated groups due to immune clearance of transgene-expressing hepatocytes, which was not observed (Fig. 4B).

We have also now included in the manuscript consideration of additional potential mechanisms, including vector integration into non-parenchymal or progenitor cell populations with higher proliferative capacity[11], which may contribute to tumour development and warrants further investigation. Accordingly, we have added the following clarifying statement to the main text: "Among other oncogenic mechanisms involving the ubiquitous EF1α promoter, we considered the possible expression of the VPS33B transgene in antigen-presenting cells, or insertion of the vector in other parenchymal cells such as hepatocyte progenitors which have higher proliferative potential (33). While we believe the first mechanism to be unlikely as this would lead to immune responses mounted against VPS33B positive hepatocytes and hence a lower VCN in tumour cells, the second warrants further investigation." (line 287-292 – highlighted in yellow). Unfortunately, lack of material and financial constrains have limited our ability to delve deeper into the second option, but further investigation could be conducted in follow up projects.

Although the precise mechanism underlying the genotoxicity observed with EF1α promoter–containing vectors remains to be fully elucidated, we believe that insertional oncogenesis, likely driven by EF1α promoter mediated dysregulation of nearby gene expression remains the most plausible explanation based on the current data.

- Integration profile from liver of animals transduced with the safe LP1 construct should have been added to show similarity/differences between the two viruses.

We thank the reviewer for this helpful suggestion and appreciate the opportunity to clarify this point. Integration profile analyses from livers of animals treated with the LP1 promoter constructs are already included in Fig. 4C-E. In these panels, data from an LP1-GFP–treated animal are shown in magenta (LG) and data from an LP1-VPS–treated animal are shown in teal (LV). Both samples were highly polyclonal and showed a high Shannon diversity index close to 1 so they were included in the healthy group for the next steps of the analysis. We acknowledge that this distinction was not sufficiently clear in the original version of the figure.

To improve clarity, we have revised Figures 4C–E to more explicitly indicate the inclusion of healthy samples from both LP1-GFP and LP1-VPS–treated animals. In addition, we made it more clear in the figure legend that the healthy group used for further analysis in (Fig. 4 F-I) contains samples originating from LG and LV treated mice as well. We would also provide the complete integration site datasets for all 11 samples analysed in Fig. 4 C-E to enable further independent evaluation by interested readers. To ensure the methodology is clearly conveyed, we have added the following clarifying statements to the main text: "samples collected from LG, LV, EG and EV animals" (line 142 – highlighted in yellow)

- Figure 4b) please change vcn to "VCN" and add the unit to the graph

Author comments:

We thank the reviewer for this helpful comment. The acronym has now been corrected to VCN, and the appropriate unit of measurement has been added to the graph in Fig. 4B

- Figure 7 d-f) graphs representing a quantification of the data shown should be provided to strengthen the results.

Author comments:

We thank the reviewer for this constructive suggestion and agree that quantitative analyses strengthen the conclusions. In response, we have now performed a comprehensive quantification of the data shown in Figure 7. Specifically, liver fibrosis has been quantified using PicroSirius red staining (Fig. 7B), and the bile canaliculi network has been analysed by quantifying the surface area covered by canaliculi (Fig. 7D–E). In addition, we characterised bile canaliculi network architecture in wild-type and LV-treated animals by measuring average branch length (Fig. 7F), branch number (Fig. 7G), and junction number (Fig. 7H). We also quantified apical membrane length based on electron microscopy images (Fig. 7J).

The figure legend has been updated accordingly, and the main text now includes a detailed quantitative description of these findings (lines 222-235 – highlighted in yellow)

- Line 217

Statement "we evaluated the in vitro and in vivo safety and efficacy of two lentiviral vector" is incorrect as safety was not evaluated for any of them. Safety includes survival, liver function, etc.

Author comments:

We thank the reviewer for their careful consideration of this point. While we appreciate and value most of the reviewer's comments, we respectfully suggest an alternative interpretation of this point. In the present study, we have addressed several key preclinical requirements that support the regulatory evaluation of gene therapy candidates[12]. These include demonstration of pharmacological proof of concept in relevant disease models (Fig. 6 and 7), assessment of vector biodistribution (Fig. 4A–B),

identification of potential on target organs toxicity (Fig. 3D–G), and evaluation of insertional genotoxicity risk through comprehensive integration site analyses (Fig. 4C–I and Fig. 5).

In addition, we have shown that animal growth and body weight remained comparable across all treatment groups and heterozygous controls throughout the study period (Fig. 3B–C), further supporting the safety profile of the therapy at this stage. Importantly, in the revised manuscript we now include new long-term safety data from VPS33B knockout animals preconditioned with clodronate liposomes and treated neonatally with the LP1-VPS vector. These data include analyses of overall survival, liver-to-body weight ratio, and tumour incidence (Fig. 3H–J), providing further evidence of sustained safety in the disease model. Of note, we have added this new data also in the main text body in lines 123-130 highlighted in yellow.

We fully acknowledge that a comprehensive regulatory safety package will require additional biodistribution and toxicology studies, which are appropriately performed at later stages of the translational pathway as part of formal regulatory toxicology programmes. However, we believe that the breadth of efficacy and safety data presented here, now strengthened by long-term follow-up in the disease model, is well aligned with the scope and objectives of a robust preclinical proof-of-concept study.

- Line 260-265

Although a few relevant papers have been cited, VSP33B is not classified as cancer-associated gene, hence the weight of evidence that VSP33B may be an oncosuppressor is missing. The statement that “the Vps33bLiver+/- mouse model, which has a single functional Vps33b allele in hepatocytes, may be prone to developing hepatocellular carcinoma” is only speculative.

Author comments:

Canonical tumour suppressor genes such as TP53 or NF1 are characterised by high mutation rates across different cancers and direct role in controlling cell proliferation or apoptosis[13]*. There are however several genes that show non-traditional tumour suppression mechanisms, which increase the risk of tumour development when mutated. We believe that the evidence presented in the provided citations clearly supports at least that VPS33B can act like a tumour suppressor despite not being classified as a canonical tumour suppressor gene. Additionally, when searching for << “VPS33B” AND “ Tumour” >> in PubMed there are about 15 articles, including the ones cited by us, highlighting involvement of this gene in various tumours. We are happy however to slightly amend the statement to make this obvious to the readers.*

We agree that the statement emphasised by the reviewer is only speculative and we are mentioning this in the discussion.

- Line 279

Is treatment with clodronate liposome a therapeutic option for patients?

Author comments:

Clodronate (Cl_2MBP) is a non-toxic bisphosphonate drug that has been used in the treatment of osteolytic bone diseases such as osteoporosis[14]. Clodronate liposomes have also been used in neonatal mice to deplete liver macrophages prior to lentiviral administration, resulting in an improved therapeutic effect[15]. Clodronate liposomes have also been previously safely administered in patients to deplete synovial macrophage for improvement of rheumatoid arthritis outcomes[16]. Recently, new bisphosphonates became more popular than clodronate for liposomal administration in patients[17] The use of clodronate liposomes was the strategy of choice to deplete macrophages for our in vivo studies, but there are other strategies that can achieve similar outcomes should one decide against a systemic administration of clodronate liposomes. For instance, lentiviral vectors that have a "don't-eat-me" signal on their envelope have been used to avoid macrophage uptake leading to increased hepatocyte transduction[18].

To clarify this, we have added the following in the main text: "We believe the macrophage depletion with clodronate liposomes or the cholic acid supplementation used in this study will not pose a barrier to clinical translation. Bisphosphonates including clodronate have been safely used in patients with various modes of administration (40, 41). In addition to liposomes containing various types of bisphosphonates other strategies are available, such as lentiviral vectors incorporating "don't-eat-me" signals to reduce macrophage uptake (39)." (line 336-341 highlighted in yellow)

- Line 283-286

Could the authors elaborate more on the role of cholic acid if hypothetically treating patients? Would it be required? Or is it just a way to exacerbate the murine phenotype to make it more similar to the human one?

Author comments:

We thank the reviewer for this thoughtful question and appreciate the opportunity to clarify the role of cholic acid in our study. Cholic acid administration would not be required as part of a therapeutic approach in patients. In this work, the 0.25% cholic acid diet was used specifically to exacerbate the

cholestatic phenotype in the murine model, thereby accelerating disease progression and more closely recapitulating the severity observed in human ARC syndrome.

In the clinical setting, we anticipate that the natural course of ARC syndrome itself would provide sufficient selective pressure to allow LP1-VPS-corrected hepatocytes to gain a competitive advantage and progressively repopulate the liver parenchyma over time, without the need for exogenous bile acid supplementation.

We have included the following in the main text for clarity "The requirement for a cholic acid diet reflects a limitation of the ARC syndrome murine disease model, as mice have a hydrophilic, muricholic acid–rich bile acid pool that attenuates cholestatic injury (42). Such dietary intervention is not anticipated in patients, as human bile acids are naturally more hydrophobic and cytotoxic providing natural selective pressure." (Line 341-344 highlighted in yellow)

- Line 292-297

In principle, this reviewer agrees with the statements, even though in their opinion this is not a case of insertional oncogenesis and other mechanisms may have been responsible. However, in agreement with the statements in line 292-297, the reviewer would suggest considering integration site analysis in relevant human cell lines (e.g. primary hepatocyte) transduced in vitro to predict IS profile in patients.

Author comments:

Lentiviral vectors are known to integrate in the gene bodies of actively transcribed genes[8]*. As the genetic expression pattern is likely different between mouse and human hepatocytes, we agree with the reviewer that integration site analysis should also be performed in primary hepatocytes. Thus, integration site analysis in primary hepatocytes transduced with the LP1-VPS vectors will be performed during the next steps in the translational pathway of the vector.*

Reviewer #2 (Remarks to the Author):

In this manuscript, Cozmescu et al. have developed two recombinant lentivirus vectors. These vectors vary in their design: one utilizes a codon-optimized cDNA of VPS33B under the control of a liver-specific promoter (LP1 named LV), and the other uses a ubiquitous promoter (EF1a named EV), both intended for the treatment of ARC syndrome caused by VPS33B deficiency. They analyzed the safety of lentiviral vector administration in heterozygous animals and found that the administration of the lentiviral vector carrying the EF1a promoter, independently of the transgene (VPS33B or GFP), induced the formation of liver malignancies, while the Lentiviral vector with the LP1 promoter did not. They also found that the Lentivirus EF1a was associated with integration in genes potentially linked to liver tumor formation. Finally, they evaluated the therapeutic efficacy of the safer LV vector administered in combination with clodronate-loaded liposomes in a model of hepatic ARC showing a beneficial effect.

These findings are very relevant and raise concerns about the safety of systemic LV administration. Although the mice receiving the vector with the LP1 promoter showed no malignancies, the authors should indicate that the vector copy number in this group was lower.

Author comments:

We thank the reviewer for highlighting the relevance of these findings and for raising this important point regarding the safety of systemic lentiviral administration. However, as shown in Fig. 4B, the vector copy number was not significantly lower in the LP1-VPS group compared with the other experimental groups when DNA was extracted from non-tumour liver tissue. Notably, the only samples displaying a marked increase in VCN were those derived from tumour tissue. We interpret this increase as being most consistent with clonal expansion, which prompted us to further investigate the potential genotoxicity associated with the EFS-GFP and EFS-VPS vectors. We would also like to note that a lower VCN in healthy tissue did not necessarily correlate with improved safety for the EFS-GFP and EFS-VPS-treated mice, as several animals that developed tumours exhibited low VCN in non-tumour liver samples. Taken together, these observations suggest that vector design and promoter choice, rather than VCN alone, likely played a more prominent role in the observed genotoxic events.

Furthermore, there are a number of important issues and inconsistencies that need to be properly addressed by the authors.

- Major Comments

Efficacy evaluation was performed in Vps33b Liver-/- animals pretreated with clodronate-loaded liposomes and receiving the vector at P4, with sacrifice 12 weeks later. In contrast, safety testing was performed in Vps33b Liver+/- animals receiving the vector at P2 in the absence of clodronate

pretreatment, with sacrifice 36 weeks later. These differences might have a significant impact on safety and are not mentioned by the authors.

Author comments:

We thank the reviewer for raising this important point and for highlighting the potential impact of the differences in experimental design between the efficacy and safety studies. We fully agree that vector administration timing, genotype, duration of follow-up, and the use of clodronate pretreatment could influence safety outcomes and therefore warrant careful consideration.

In response, we have now performed an additional long-term safety study in Vps33b Liver$^{-/-}$ neonates pretreated with clodronate-loaded liposomes prior to receiving the LP1-VPS vector, thereby matching the disease-relevant and macrophage-depleted conditions used in the efficacy experiments. Neonatal mice were treated with 5 × 10^{10} TU/kg LP1-VPS (n=5) or with control PBS injections (n=5) and followed for 9 months. With the exception of one mouse, all LP1-VPS–treated animals survived to the end of the study (Fig. 3H), and no tumours were observed in this group. By contrast, one untreated animal developed a tumour (Fig. 3I-J).

These new data have now been incorporated into Fig. 3, providing direct evidence supporting the safety and lack of tumour development associated with LP1-VPS administration in the disease-relevant knockout model combined with clodronate-mediated macrophage depletion.

We have also included the relevant results section and discussion in the main body of text:

"The LP1-VPS safety was further evaluated in the Vps33bLiver-/- disease model following the above protocol. To enhance hepatocyte transduction (19), mice received intraperitoneal clodronate liposomes to transiently deplete liver and splenic macrophages (20, 21) prior to lentiviral vector (Clod.LV, n=5) or PBS (n=5) administration. Except for one mouse in the Clod.LV group, which was culled 2 days post-LV administration, all animals survived until the experimental endpoint (Fig. 3H) and displayed comparable median liver mass at harvest (Fig. 3I). No liver abnormalities were observed in Clod.LV-treated animals, in contrast to a single PBS-treated Vps33bLiver-/- mouse (Fig. 3J) which also had an increased liver mass (Fig. 3I)." (line 123-130 highlighted in yellow)

"LP1-VPS vectors with or without clodronate administration" (line 315 highlighted in yellow)

The authors should clearly indicate in the abstract that the treatment consisted of the administration of the viral vector together with macrophage depletion using clodronate.

Author comments:

We thank the reviewer for this helpful suggestion and have now updated the abstract to clearly indicate that the treatment consisted of viral vector administration in combination with macrophage depletion using clodronate. "combined with transient liver macrophage depletion" line 32-33 highlighted in yellow.

They should also discuss whether this protocol can be transferred to the clinic.

Author comments:

We thank Reviewer 2 for raising this important point, which echoes a concern also highlighted by Reviewer 1. Clodronate (Cl_2MBP) is a non-toxic bisphosphonate drug that has been used in the treatment of osteolytic bone diseases such as osteoporosis[14]. Clodronate liposomes have also been used in neonatal mice to deplete liver macrophages prior to lentiviral administration, resulting in an improved therapeutic effect[15]. Clodronate liposomes have also been previously safely administered in patients to deplete synovial macrophage for improvement of rheumatoid arthritis outcomes[16]. Recently, new bisphosphonates became more popular than clodronate for liposomal administration in patients[17] The use of clodronate liposomes was the strategy of choice to deplete macrophages for our in vivo studies, but there are other strategies that can achieve similar outcomes should one decide against a systemic administration of clodronate liposomes. For instance, lentiviral vectors that have a "don't-eat-me" signal on their envelope have been used to avoid macrophage uptake leading to increased hepatocyte transduction[18].

To clarify this, we have added the following in the main text: "We believe the macrophage depletion with clodronate liposomes or the cholic acid supplementation used in this study will not pose a barrier to clinical translation. Bisphosphonates including clodronate have been safely used in patients with various modes of administration (40, 41). In addition to liposomes containing various types of bisphosphonates other strategies are available, such as lentiviral vectors incorporating "don't-eat-me" signals to reduce macrophage uptake (39)." (line 336-341 highlighted in yellow)

Figure 6 presents very important issues.

To evaluate the therapeutic efficacy of the vector one month after treatment, LV animals showed an 80% survival rate, compared to 33.3% for LG-treated animals. If the experiment was performed for 12 weeks, why are the authors not representing the follow-up data to the end of the experiment?

Author comments:

We thank the reviewer for this helpful comment and for prompting us to clarify this point. In the original version of the manuscript, Figure 6B–E presented data collected during the first four weeks of cholic acid administration, as all mortality occurred within this initial period and no additional deaths were observed thereafter until the end of the 12-week experiment. We therefore considered the early time points sufficient to illustrate the therapeutic effect.

However, to improve clarity and fully address the reviewer's concern, we have now updated the survival analysis to include follow-up data for the entire duration of the experiment. The revised figure now shows animal survival through to the end of the 12-week study period, confirming that no further mortality occurred beyond the first month. We believe this revision provides a clearer and more complete representation of the long-term survival outcome.

More importantly, if the study started with 6 animals per group, by week 8 of age (4 weeks after CA diet), only 2 animals of the LG group would remain alive. However, in the ALP value at 12 weeks of age, data from 6 animals are represented, which is not possible. The same applies to the values in the rest of the graphs, which, according to the legend, were obtained at harvest in n = 6 in all the groups, in the LG group should be 2 and in the LV group 5. A potential explanation to this incongruency is that the values were obtained at sacrifice but this should be clearly explained.

Author comments:

We thank the reviewer for this careful observation and apologise for the lack of clarity in the original presentation. The reviewer is correct in noting that the apparent discrepancy arises from the timing of data collection. The data shown in panels F-L were collected at sacrifice/harvest, rather than at a fixed 12-week time point. Unfortunately, in panel F the label "12w" was mistakenly used instead of "at sacrifice," which contributed to this confusion.

We also acknowledge that we used the terms harvest and sacrifice interchangeably in the original manuscript, which may have further obscured this point. In the revised version, we have corrected the figure labelling and clarified in both the Figure 6 legend and the main text that these analyses were performed on samples collected from the animals at sacrifice regardless of the time of death. We have explicitly added the terms "the live" (lines 196 and 198 highlighted in yellow) and "at sacrifice" (line 199 highlighted in yellow) where appropriate to ensure that the timing and sample numbers are unambiguous and that the data are accurately interpreted for both the live and post-mortem analysis.

Supplementary Figures 2 and 3 deserve a better description, explanation, and discussion of the data. Why does the "therapeutic" efficacy of LV seem to be better in the absence of clodronate than when clodronate was administered and transduction improved?

Author comments:

We agree a better description and explanation could have been provided for the Supplementary Figures 2 and 3. However, we kindly disagree with the reviewer's statement that "the "therapeutic" efficacy of LV seem to be better in the absence of clodronate than when clodronate was administered and transduction improved". Supplementary Figure 3 presents the comparison of LV vs combined administration of clodronate and LV (Clod.LV) in restoring the ARC related liver phenotype. Clod.LV combination had a trend of improvement in all the parameters measured for the comparison.

We have however tweaked the main body of text to better describe the Fig. S2 and Fig. S3. "Initial experiments have revealed a phenotypic drift when using the established protocol of liver phenotype induction by 0.5% cholic acid (CA) feeds (3) (Fig. S5), which lead to acute liver injury and death, so the following experiments were conducted using a 0.25% CA diet. In addition, we conducted preliminary optimisation experiments to assess the impact of clodronate liposome administration on vector uptake (Fig. S6). Based on these findings, intraperitoneal clodronate treatment was incorporated into the final protocol to transiently deplete liver and splenic macrophages (20, 21), enhancing liver transduction (19)." (lines 179-185 highlighted in yellow)

Ha really LV provide a selective advantage) is really therapeutic?
In fact, the authors claim that the increase in VCN levels was due to a selective advantage of transduced hepatocytes. However, if this is the case, a significant difference should be observed between the LG group (which does not have a selective advantage) and the LV group. In fact, GFP expression levels are higher than VPS33B.

Author comments:

We thank the reviewer for this insightful comment and appreciate the opportunity to clarify our interpretation. Vector copy number (VCN) represents an average measure of integration across the analysed cell population. While we agree that no statistically significant difference in VCN was observed between the LG and LV groups, we believe there are plausible explanations for the observed values. First, LG vectors contain a smaller transgene cassette, which may confer higher transduction efficiency and result in cells harbouring, on average, a higher VCN. If such cells subsequently proliferate during liver growth or regeneration, this could contribute to an increased average VCN. Second, although DNA was consistently extracted from the same liver region across experiments, local variability in the proportion of transduced cells within the sampled area could also influence the measured average VCN.

With respect to the higher GFP expression compared with VPS33B following treatment, this observation is expected and does not reflect a competitive advantage of the GFP-expressing vector. GFP is substantially smaller than VPS33B, which may result in more efficient transcription and higher mRNA

abundance. In addition, the two transcripts are likely subject to distinct post-transcriptional regulatory mechanisms and mRNA stability profiles. To avoid distraction from the main biological message and given the multiple factors that could contribute to these differences, we have moved the GFP expression data to the Supplementary Information.

Taking these considerations into account, and in light of the observation that larger liver areas display functional bile canaliculi following prolonged treatment (Fig. 7C-E) compared with animals exposed to a shorter selective pressure (Fig. S2K), we believe the data remain consistent with amplification driven by a selective advantage. However, we recognise the importance of cautious interpretation and have therefore softened the language throughout the manuscript to more appropriately reflect the supportive, rather than definitive, nature of this conclusion by including "possibly" in line 201 and "a possible" in line 332.

Additional Comments:

- Introduction

"Lentiviral vectors (LV) have been used in ex vivo and in vivo clinical trials and received regulatory approvals for the treatment of monogenic disorders6–8" No clinical data are reported in those references for in vivo clinical trials; please modify this statement.

Author comments:

We thank the reviewer for noticing this. We have now revised the Introduction to accurately reflect the available clinical evidence and have included the following reference reporting in vivo clinical data: Palfi et al., Long-Term Follow-Up of a Phase I/II Study of ProSavin, a Lentiviral Vector Gene Therapy for Parkinson's Disease (Hum Gene Ther Clin Dev, 2018).

- Results

VPS33B protein analysis in the liver by immunohistochemistry should be included, and the levels of fibrosis and bile duct numbers should be quantified, plotted, and statistically analyzed.

Author comments:

We thank the reviewer for this constructive suggestion. In regard to VPS33B protein detection by immunohistochemistry, despite extensive optimisation efforts and prior experience, the available antibodies do not reliably detect VPS33B in liver tissue by immunohistochemistry. To address this

limitation, we have now included mass spectrometry–based proteomic data demonstrating the presence of human VPS33B protein in the livers of experimental animals following lentiviral treatment (Fig. 6P), providing independent and robust confirmation of transgene expression in vivo.

In response to the reviewer's request for quantitative analyses of fibrosis and bile duct architecture, we have now performed a comprehensive quantification of the data presented in Fig. 7. Specifically, liver fibrosis was quantified using PicroSirius red staining (Fig. 7B), and the bile canaliculi network was analysed by measuring the surface area covered by canaliculi (Fig. 7D–E). In addition, we characterised canalicular network architecture by quantifying average branch length (Fig. 7F), branch number (Fig. 7G), and junction number (Fig. 7H), and we also quantified apical membrane length from electron microscopy images (Fig. 7J).

The figure legends have been updated accordingly, and the main text now includes a detailed quantitative description of these findings (lines 222–235, highlighted in yellow). We believe these additions substantially strengthen the quantitative and mechanistic support for our conclusions.

- Discussion

The authors should highlight the limitation of the model, since liver pathology has to be induced by a cholic acid diet.

Author comments:

We thank the reviewer for this insightful suggestion and welcome the opportunity to further clarify the use of cholic acid in our study. Mice have a hydrophilic, muricholic acid dominated bile acid pool that make them more resistant to cholestatic liver damage[19]*. In this work, the 0.25% cholic acid diet was used solely to aggravate the cholestatic phenotype in the murine model, thereby accelerating disease progression to more closely replicate the severity of human ARC syndrome.*

In humans, the bile acid pool is more hydrophobic and damaging. Thus, we anticipate that the intrinsic progression of ARC syndrome would itself generate sufficient damage to allow LP1-VPS–corrected hepatocytes to potentially acquire a competitive advantage and gradually repopulate the liver parenchyma, without the need for extra interventions.

To ensure clarity, we have added the following statement to the main text: "The requirement for a cholic acid diet reflects a limitation of the ARC syndrome murine disease model, as mice have a hydrophilic, muricholic acid–rich bile acid pool that attenuates cholestatic injury (42). Such dietary intervention is not anticipated in patients, as human bile acids are naturally more hydrophobic and cytotoxic, thereby providing intrinsic selective pressure." (Lines 333–336, highlighted in yellow).

Supporting references:

1. Lindeboom, R. G. H., Vermeulen, M., Lehner, B. & Supek, F. The impact of nonsense-mediated mRNA decay on genetic disease, gene editing and cancer immunotherapy Europe PMC Funders Group. https://doi.org/10.6084/m9.figshare.7803398 (2020) doi:10.6084/m9.figshare.7803398.
2. Hanley, J. *et al.* Vps33b is crucial for structural and functional hepatocyte polarity. *J. Hepatol.* **66**, 1001–1011 (2017).
3. Van der Wouden, J. M., Van IJzendoorn, S. C. D. & Hoekstra, D. Oncostatin M regulates membrane traffic and stimulates bile canalicular membrane biogenesis in HepG2 cells. *EMBO Journal* **21**, 6409–6418 (2002).
4. Cullinane, A. R. *et al.* Mutations in VIPAR cause an arthrogryposis, renal dysfunction and cholestasis syndrome phenotype with defects in epithelial polarization. *Nat. Genet.* **42**, 303–312 (2010).
5. Rogerson, C. & Gissen, P. VPS33B and VIPAR are essential for epidermal lamellar body biogenesis and function. *Biochim. Biophys. Acta Mol. Basis Dis.* **1864**, 1609–1621 (2018).
6. Wang, C. *et al.* Vacuolar Protein Sorting 33B Is a Tumor Suppressor in Hepatocarcinogenesis. *Hepatology* **68**, 2239–2253 (2018).
7. Chen, Y. *et al.* VPS33B negatively modulated by nicotine functions as a tumor suppressor in colorectal cancer. *Int. J. Cancer* **146**, 496–509 (2020).
8. Cavazza, A., Moiani, A. & Mavilio, F. Mechanisms of retroviral integration and mutagenesis. *Human Gene Therapy* vol. 24 119–131 Preprint at https://doi.org/10.1089/hum.2012.203 (2013).
9. Duncan, C. N. *et al.* Hematologic Cancer after Gene Therapy for Cerebral Adrenoleukodystrophy. *New England Journal of Medicine* **391**, 1287–1301 (2024).
10. Modlich, U. *et al.* Insertional transformation of hematopoietic cells by self-inactivating lentiviral and gammaretroviral vectors. *Molecular Therapy* **17**, 1919–1928 (2009).
11. Gromowski, T., Lukacs-Kornek, V. & Cisowski, J. Current view of liver cancer cell-of-origin and proposed mechanisms precluding its proper determination. *Cancer Cell International* vol. 23 Preprint at https://doi.org/10.1186/s12935-022-02843-0 (2023).
12. White, M., Whittaker, R., Gándara, C. & Stoll, E. A. A Guide to Approaching Regulatory Considerations for Lentiviral-Mediated Gene Therapies. *Human Gene Therapy Methods* vol. 28 163–176 Preprint at https://doi.org/10.1089/hgtb.2017.096 (2017).
13. Swale, V. J. & Quinn, A. G. Tumour suppressor genes. *Clin. Exp. Dermatol.* **25**, 231–235 (2000).
14. Frediani, B., Baraldi, E. & Cremonesi, G. Effect of Clodronate Treatment on Risk of Fracture: A Systematic Review and Meta-analysis. *Calcified Tissue International* vol. 95 295–307 Preprint at https://doi.org/10.1007/s00223-014-9903-2 (2014).
15. Touramanidou, L. *et al.* Macrophage Inhibitor Clodronate Enhances Liver Transduction of Lentiviral but Not Adeno-Associated Viral Vectors or mRNA Lipid Nanoparticles in Neonatal and Juvenile Mice. *Cells* **13**, 1979 (2024).
16. Barrera, P. *et al.* Synovial macrophage depletion with clodronate-containing liposomes in rheumatoid arthritis. *Arthritis Rheum.* **43**, 1951–1959 (2000).
17. La-Beck, N. M., Liu, X., Shmeeda, H., Shudde, C. & Gabizon, A. A. Repurposing amino-bisphosphonates by liposome formulation for a new role in cancer treatment. *Seminars in Cancer Biology* vol. 68 175–185 Preprint at https://doi.org/10.1016/j.semcancer.2019.12.001 (2021).
18. Milani, M. *et al.* Phagocytosis-shielded lentiviral vectors improve liver gene therapy in nonhuman primates. *Sci. Transl. Med.* **11**, (2019).
19. Straniero, S. *et al.* Of mice and men: Murine bile acids explain species differences in the regulation of bile acid and cholesterol metabolism. *J. Lipid Res.* **61**, 480–491 (2020).

REVIEWERS' COMMENTS

Reviewer #1 (Remarks to the Author):

I commend the authors for addressing most of the concerns through the provision of additional data that now better support the in vitro statements. Regarding insertional oncogenesis, although I do not entirely agree with their interpretation, the data are adequate to enable readers to form their own opinion, and it is beneficial to have these findings reported.

Author comments:

We thank the reviewer for their constructive and thoughtful comments. We appreciate their recognition that the additional data strengthen the in vitro findings. We also acknowledge their perspective on insertional oncogenesis and agree that presenting these data allows readers to form their own informed conclusions.

Reviewer #2 (Remarks to the Author):

The authors have made a significant effort to address the reviewers' questions, which is greatly appreciated. However, two points still require attention.

Author comments:

We thank the reviewer for their careful evaluation and constructive comments. We appreciate the acknowledgment of our efforts to address the initial concerns. The points raised regarding the translatability of macrophage depletion strategies and the appropriate contextualization of lentiviral vector clinical use are well taken. Below, we detail how we have addressed each of these issues in the revised manuscript.

First, the clinical use of agents to deplete macrophages in the liver typically requires systemic administration, whereas the examples provided by the authors are based on local injection. The authors should therefore be cautious when discussing the translatability of this approach.

Author comments:

Included additional explanation and clarification in lines 339-348: 'Whilst clodronate liposome approach used here to selectively deplete liver macrophages is not currently an established clinical intervention our findings indicate its utility to enhance lentiviral delivery to hepatocytes. Clinically relevant bisphosphonates, including clodronate, have been safely administered in patients through multiple

routes[41,42]. Liposomal formulations of alendronate have demonstrated effective macrophage targeting and tolerability in non-human primate models[43]. Furthermore, in a double-blind clinical trial in diabetic patients undergoing percutaneous coronary intervention, liposomal alendronate was well tolerated without increased adverse events, supporting its clinical safety despite limited efficacy in this setting[44]. In addition to liposomes containing various types of bisphosphonates other strategies are available, such as lentiviral vectors incorporating "don't-eat-me" signals to reduce macrophage uptake[40]. These approaches suggest that overcoming the hepatic macrophage barrier may be clinically achievable.'

Second, the reference used to support the statement "Lentiviral vectors (LV) have been used in ex vivo and in vivo clinical trials and received regulatory approvals for the treatment of monogenic disorders" (Palfi et al., Long-Term Follow-Up of a Phase I/II Study of ProSavin, a Lentiviral Vector Gene Therapy for Parkinson's Disease, Human Gene Therapy Clinical Development, 2018) is not appropriate. Parkinson's disease is not a monogenic disorder, and the vector administration in this study was not systemic. In fact, one of the limitations of lentiviral vectors for systemic delivery is the lack of clinical experience, and the authors should clearly acknowledge this point.

Author comments:

Clarified and included recent clinical trial information in lines 67-72: 'Lentiviral vectors (LV) have received regulatory approvals in ex vivo gene therapies for monogenic disorders and have been successfully delivered in vivo through local administration in clinical trials for Parkinson's disease[7–10]. More recently, they have also showed favourable safety profiles in early-phase in vivo clinical trials, including emerging systemic delivery for direct immune-cell engineering such as in vivo CAR-T approaches[11].'

Reviewer #2 (Remarks on code availability):

Yes they provide enough information to run the application

Author comments:

We thank the reviewer for their positive assessment and for confirming that sufficient information has been provided to run the application.
